# Advancing the Identification of Bioactive Molecules and the Construction of a Synergistic Drug Delivery System in Combating Lung Injury

**DOI:** 10.1002/advs.202407802

**Published:** 2025-03-17

**Authors:** Jianhong Qi, Yanxia Wang, Huan Chen, Kaitian Wu, Pei Zhou, Yue Dou, Bingqi Xiong, Wei Zhou

**Affiliations:** ^1^ Department of Pharmaceutics China Pharmaceutical University #24 Tong Jia Xiang, Gulou District Nanjing 210009 China

**Keywords:** glabridin, lung injury, puerarin, Traditional Chinese Medicine

## Abstract

In recent years, pneumonia caused by multiple viruses has posed a significant threat to public health, particularly affecting vulnerable populations such as the elderly and immunocompromised individuals. Current treatments primarily focused on antiviral medications, lacking “miracle cure” and innovative approaches for the pathological damage caused by viruses. Since 2019, Traditional Chinese Medicine (TCM) has shown remarkable efficacy in treating coronavirus disease 2019 (COVID‐19). However, the application is hindered by intricate mechanisms, variable quality, and slow onset. Clinically, Ge‐Gen Decoction (GGD) effectively reduced the severity in patients with viral infections. Taking COVID‐19 as a case, the bioactive ingredients from GGD: glabridin (GLA) and puerarin (PUE) are identified. Interestingly, it was discovered that PUE can self‐assemble into a 3D hydrogel structure upon heating and cooling, namely PUE@gel. This process mirrored the formation of gel‐like precipitates in GGD post‐decoction. Motivated by this phenomenon, a decoction‐mimicking drug delivery system, glabridin─puerarin self‐assembled hydrogel (GLA‐PUE@gel) was constructed, which exhibits strong anti‐inflammatory and antioxidant properties, comparable to GGD at the same dosage. Additionally, PUE that has a high biosafety threshold can competitively bind to angiotensin converting enzyme 2 (ACE2) on host cells, preventing SARS‐CoV‐2 from invading. This study offered a promising approach for treating virus‐induced lung injury.

## Graphical abstract

Traditional Chinese Medicine (TCM), while holistic and historically esteemed, faces challenges in “miracle cures” due to slow onset, long cycles, and difficulty controlling quality. This study obtains the active ingredients, glabridin (GLA) and puerarin (PUE), from Ge‐Gen Decoction (GGD), developing a safe and effective drug delivery system, GLA‐PUE@gel, with significant potential to reduce severe lung injury in clinical settings.

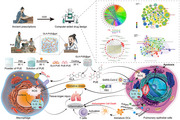

## 1 Introduction

Viral pneumonia is an interstitial lung inflammation caused by the invasion of the virus into pulmonary epithelial cells through the respiratory tract. Its pathogenesis is often related to inflammatory storms and oxidative stress. Under normal circumstances, symptoms of viral pneumonia are mild and self‐limiting, but severe cases can lead to sepsis, hypercapnia, hypoxemia, acute respiratory failure, circulatory system failure, and even pose a threat to life. In particular, highly contagious and fatal viral pneumonia, if not prevented and controlled promptly, can lead to significant public health incidents, causing long‐term and sustained negative impacts on social and economic operations. Since the discovery of coronavirus disease 2019 (COVID‐19) patients in December 2019, the outbreak of SARS‐CoV‐2 had spread to more than 200 countries, and the World Health Organization (WHO) had classified it as a global pandemic.^[^ ^1^ ^]^ In recent years, “three‐in‐one epidemic” (COVID‐19, influenza, and respiratory syncytial virus) of concurrent outbreaks had a significant impact on the elderly population. Due to the weakened immune systems, they were more susceptible to severe illness. For thousands of years, Traditional Chinese Medicine (TCM) had played an important role in the prevention and treatment of epidemic diseases, and showed the remarkable characteristic advantages.^[^ ^2^ ^]^ The National Health Commission of the People's Republic of China had launched a treatment plan with Chinese medicine and found that it had significant effects on anti‐virus, anti‐inflammatory, and alleviating the symptoms of COVID‐19 patients.^[^ ^3^ ^]^

Ge‐Gen Decoction (GGD) originated from “Treatise on Coldness‐induced Diseases”, which was a typical pungent‐warm relieving agent and was often used to treat exogenous wind‐cold exterior syndrome.^[^ ^4^ ^]^ GGD has demonstrated good clinical efficacy in treating upper respiratory tract diseases caused by viruses such as SARS‐CoV‐2,^[^ ^5^ ^]^ influenza viruses,^[^ ^4^, ^6^ ^]^ and respiratory syncytial virus.^[^ ^7^ ^]^ Referring to the ancient books of TCM such as “Yellow Emperor's Inner Classic” and “Treatise on Coldness‐induced Diseases”, it has been postulated that GGD may be efficacious in the clinical management of COVID‐19, with promising therapeutic outcomes reported.^[^ ^8^ ^]^ Furthermore, affirming its contemporary relevance and potential utility, the Health Commission of Shandong Province has formally integrated GGD into the strategic treatment guidelines for combating COVID‐19, as of December 2022.^[^ ^5^ ^]^ Significantly, GanCao (*Glycyrrhiza uralensis* Fisch) and GeGen (*Pueraria lobata* (Willd.) Ohwi) in GGD stood out as a pivotal medicinal combination, demonstrating the capacity to notably diminish the risk of severe outcomes in patients afflicted with COVID‐19.^[^ ^9^ ^]^ Puerarin (PUE), one of the main active ingredients in GeGen, had been reported to possess potent antiviral properties. It exhibited inhibitory effects against SARS‐CoV‐2 by interfering with binding of viral S‐protein and human angiotensin converting enzyme 2 (ACE2), thereby preventing the virus from entering host cells.^[^ ^10^ ^]^ Furthermore, it may alleviate inflammation and modulate the host's immune response to improve virus‐induced lung injury.^[^ ^11^ ^]^ In treating influenza, GGD exhibited notable efficacy by reducing H1N1 viral titers in vivo, demonstrating significant anti‐influenza A virus activity. It also reduced tumor necrosis factor‐alpha (TNF‐*α*) expression, improving T‐helper 1/T‐helper 2 (Th1/Th2) immune balance to regulate the body's immune response.^[^ ^4^ ^]^ Additionally, GGD promoted the nuclear translocation of nuclear factor erythroid 2‐related factor 2 (Nrf2), enhancing transcription and expression of downstream antioxidant enzymes, thereby displaying potent antioxidant effects.^[^ ^6^ ^]^ For the treatment of respiratory syncytial virus, GGD can inhibit plaque formation by promoting the secretion of interferon‐*β* and preventing the virus from adhering to respiratory epithelium, thereby reducing virus‐induced pathological damage.^[^ ^7^ ^]^ TCM, while holistic and historically esteemed, often grappled with the challenge of pinpointing a “miracle cure”, beset by factors such as slow onset, long cycle, and difficulty in controlling quality. Harnessing the potent active ingredients from Chinese medicines and refining them into a “miracle drug” could indeed represent an efficacious approach to counteracting the inflammation and oxidative stress triggered by virus.

We have sought and developed safe and efficient anti‐inflammatory and antioxidant agents from TCM through a multidisciplinary strategy encompassing bibliometrics, bioinformatics, pharmacology, pharmaceutics, and toxicology. This initiative is designed to offer valuable insights for the amelioration of viral‐induced lung injury.

## 2 Results

### 2.1 Frontier and Hot Topics of TCM in Treating COVID‐19

Web of Science (WOS) is a widely recognized comprehensive academic information platform. We obtained 681 records of TCM against COVID‐19 in WOS, and analyzed the data by CiteSpace 6.1.6 software.^[^ ^12^ ^]^

**Figure 1:**
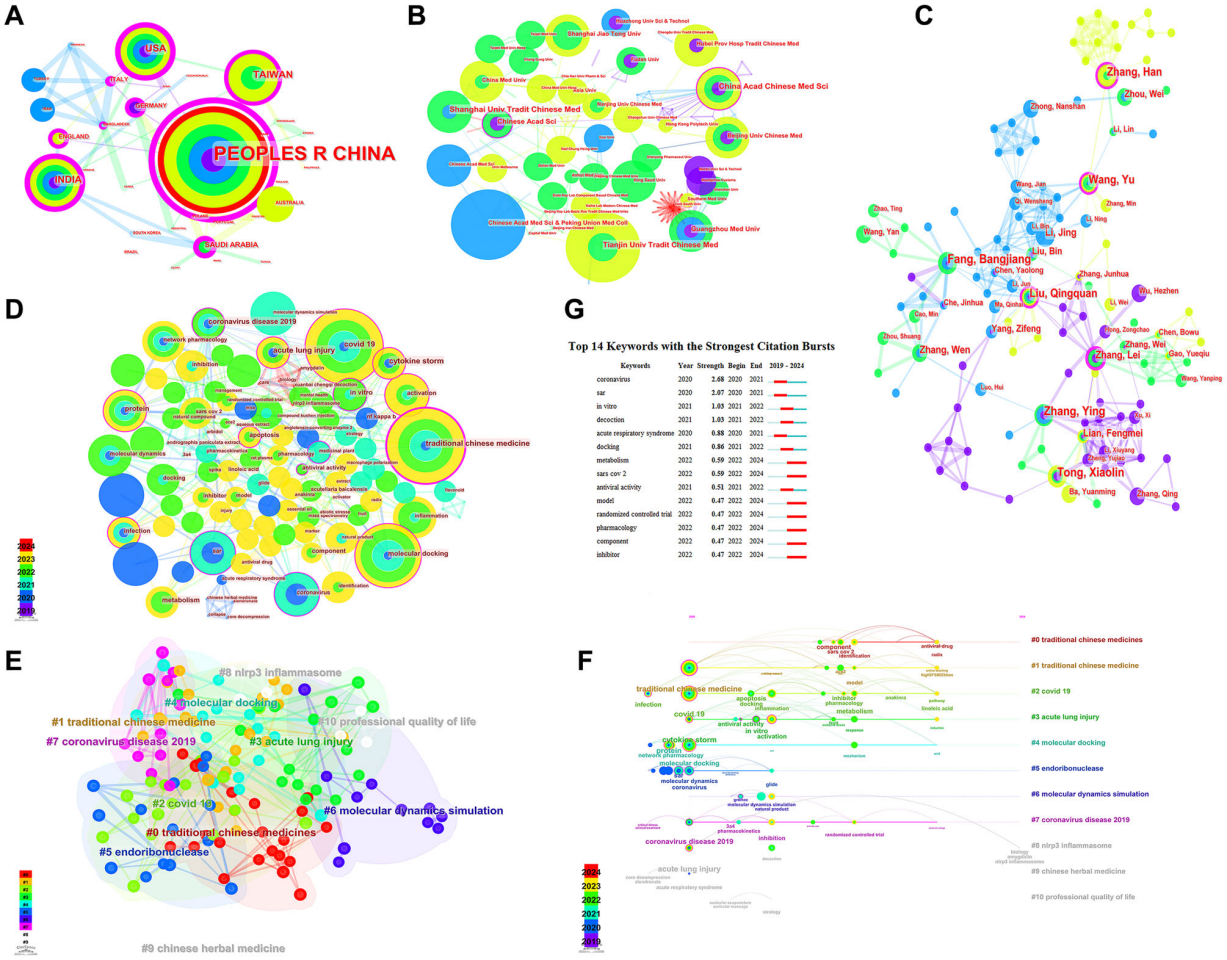
Frontier and hot topics of TCM in treating COVID‐19. A) Collaboration network of TCM anti‐COVID‐19 related articles publishing countries and regions. B) Collaboration network of TCM anti‐COVID‐19 related articles publishing institutions. C) Author cooperation network of TCM anti‐COVID‐19 related articles. D) Keywords co‐occurrence network of TCM anti‐COVID‐19 related articles. E) Keywords clustering themes of TCM anti‐COVID‐19 related articles. F) Keywords timelines of TCM anti‐COVID‐19 related articles. G) Keywords with the strongest citation bursts.

We conducted analyses of countries and regions, institutions, and author collaboration networks, as well as keyword co‐occurrence, cluster themes, timelines, and citation bursts to examine the research hotspots and trends in the treatment of COVID‐19 with TCM. The People's Republic of China showed the highest level of engagement, publishing 142 articles, with India and the USA also demonstrating significant interest. However, the scope of international collaboration was limited, exhibiting a certain degree of regionality, and there was a need for expanded cooperation among different countries (**Figure** **1**A; Table S1, Supporting Information). Furthermore, the China Academy of Chinese Medical Sciences, Shanghai University of Traditional Chinese Medicine, and Tianjin University of Traditional Chinese Medicine ranked at the forefront in terms of their focus on TCM for COVID‐19 treatment. Nevertheless, the collaboration among research institutions was narrow, with a regional bias, indicating a potential for enhanced partnerships (Figure 1B; Table S2, Supporting Information). We conducted a visual analysis of the authors (Figure 1C), and calculated the minimum number of articles published by the core authors, as calculated by Price Equation, was given by the Equation (1):

(1) $$M=0.749{\left(N_{max}\right)}^{1/2}$$

where *M* represented the minimum number of articles published by the authors, and *N_max_* was the number of articles published by the most prolific author.

The study found that the number of core authors in the field of TCM treatment of COVID‐19 was not less than 3, and 48 core authors were identified, accounting for 20.96% of the total number of authors. The top ten core authors, in terms of articles count, were as follows: Fang Bangjiang (11), Tong Xiaolin (11), Zhang Ying (11), Wang Yu (9), Liu Qingquan (9), Zhang Han (9), Zhang Wen (7), Li Jing (7), Lian Fengmei (7), and Zhang Lei (7). According to Table S3 (Supporting Information), several collaborative teams have been established, centered around Zhang Lei, Liu Qingquan, Wang Yu, and Fang Bangjiang, indicating close internal cooperation within these groups.

Pruning was set as pathfinder, pruning sliced networks and pruning the merged network, and the data was analyzed to construct the keyword co‐occurrence map (Figure 1D; Table S4, Supporting Information). Here, a total of 169 keywords were obtained, including 16 important keywords (centrality ≥ 0.1) (values in parentheses: the former represents centrality, and the latter represents frequency): Traditional Chinese Medicine (0.27, 29), protein (0.19, 10), acute lung injury (0.18, 12), cytokine storm (0.18, 6), SARS (0.17, 7), coronavirus disease 2019 (0.17, 6), in vitro (0.16, 6), molecular docking (0.15, 19), COVID‐19 (0.13, 23), activation (0.13, 9), apoptosis (0.12, 5), medicinal plant (0.12, 2), antiviral activity (0.11, 3), coronavirus (0.1, 9), infection (0.1, 7), and main protease (0.1, 2). All keywords can be summarized into four categories: research themes (2), research methods (2), diseases and symptoms (6), and outcome indicators (6). Cluster analysis of the keywords was performed (Figure 1E), retaining the top 11 clusters: Traditional Chinese Medicines, Traditional Chinese Medicine, COVID‐19, acute lung injury, molecular docking, endoribonuclease, molecular dynamics simulation, coronavirus disease 2019, NLRP3 inflammasome, Chinese herbal medicine, and professional quality of life. The cluster Q value was 0.6531, and the S value was 0.8409, indicating reliable clustering. In addition, a timeline analysis of the keywords was conducted, revealing that drug‐drug interaction, care, anti‐SARS‐CoV‐2 drug, Xuanbai Chengqi Decoction, biology, amygdalin, NLRP3 inflammasome, macrophage polarization, injury, and antiviral drugs were at the forefront of research keywords (Figure 1F). Finally, the burst words that increase sharply in a short period of time can be found in a specific year, so as to reveal the duration and trend of the hot topics. As shown in Figure 1G, terms such as “coronavirus”, “SARS”, “in vitro”, and “decoction” exhibit high burst strength, while terms like “metabolism”, “SARS‐CoV‐2”, “model”, “randomized controlled trial”, “pharmacology”, “component”, “inhibitor” remain as hot topics in the research on TCM's treatment of COVID‐19 in recent years. Therefore, it was imperative to elucidate the molecular mechanisms of TCM in treating COVID‐19 and other viral diseases.

### 2.2 Molecular Mechanism of GanCao─GeGen in Treating COVID‐19

GanCao─GeGen was identified as an effective Chinese herbal pair for the treatment of COVID‐19. However, its molecular mechanism was still unclear.

**Figure 2:**
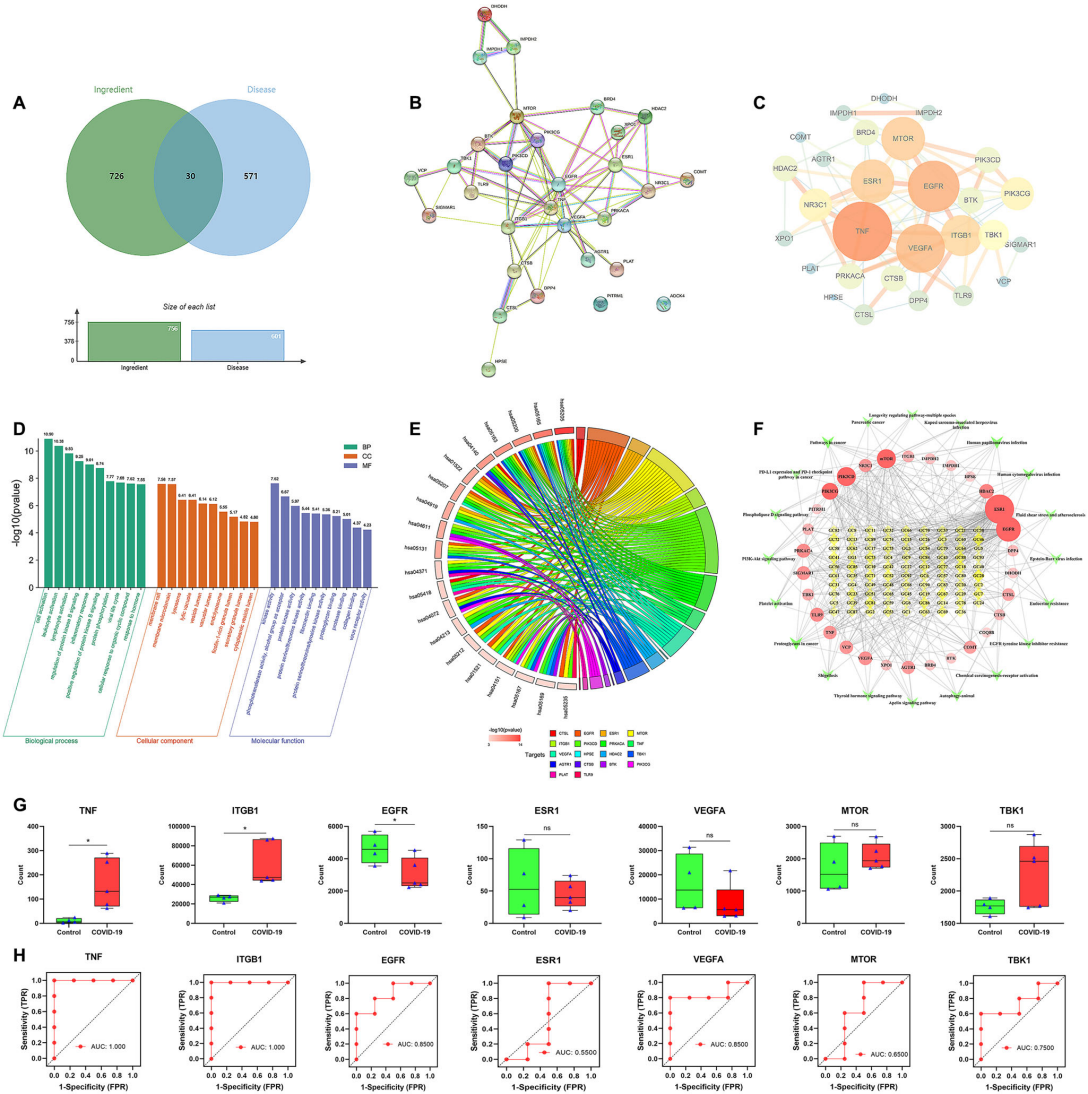
Molecular mechanism of GanCao─GeGen in treating COVID‐19. A) Venn diagram illustrating the overlap between the targets associated with ingredients in GanCao─GeGen and those related to COVID‐19. B) Original graph of PPI network: the nodes are proteins, and edges of different colors represent protein−protein relationships. Light blue and purple edges mean known interactions (database & experiment); green, red, and blue edges mean predicted interactions (gene neighborhood, gene fusions, gene co‐occurrence); yellow edges mean text mining; and black edges indicate co expression. C) Network diagram after Cytoscape processing: the size of nodes indicates the importance of targets (larger nodes denote more important targets). D) Enrichment analysis of GO function: biological process, cellular components, and molecular functions. E) Enrichment analysis of KEGG annotation: signaling pathways. F) Ingredient─target─disease network: yellow denotes the ingredients from GanCao─GeGen, red signifies the targets, and green indicates the signaling pathways. The size of each node corresponds to its connectivity, with larger nodes indicating higher importance due to the greater number of associated edges. G) mRNA level of core targets in COVID‐19 patients (*p < 0.05). H) Diagnostic ROC curve of core targets in COVID‐19 patients.

First, we collected 101 potential active ingredients from GanCao─GeGen, predicted and obtained 756 ingredient‐targets and 601 disease‐targets, of which 30 were the intersection targets of GanCao─GeGen in the treatment of COVID‐19 (**Figure** **2**A). Then, a PPI network between targets was constructed on the STRING 12.0 platform to further get core targets (Figure 2B,C): Epidermal growth factor receptor (EGFR), tumor necrosis factor (TNF), vascular endothelial growth factor A (VEGFA), mechanistic target of rapamycin (MTOR), estrogen receptor 1 (ESR1), integrin subunit beta 1 (ITGB1), and tank‐binding kinase 1 (TBK1). In addition, we conducted Gene Ontology (GO) and Kyoto Encyclopedia of Genes and Genomes (KEGG) analysis on the Metascape 3.5 platform (Figure 2D,E). All items were sorted from small to large according to *p* value, and the related biological process (BP) included cell activation, leukocyte activation, lymphocyte activation, regulation of protein kinase B signaling, inflammatory response, positive regulation of protein kinase B signaling, protein phosphorylation, viral life cycle, cellular response to organic cyclic compound, and response to hormone. The related cellular components (CC) included membrane raft, membrane microdomain, lysosome, lytic vacuole, vesicle lumen, vacuolar lumen, endolysosome, ficolin‐1‐rich granule lumen, secretory granule lumen, and cytoplasmic vesicle lumen. The related molecular functions (MF) included kinase activity, phosphotransferase activity, alcohol group as acceptor, protein kinase activity, protein serine/threonine kinase activity, fibronectin binding, protein serine/threonine/tyrosine kinase activity, proteoglycan binding, protease binding, collagen binding, virus receptor activity. The related signaling pathways included proteoglycans in cancer, human papillomavirus infection, pathways in cancer, human cytomegalovirus infection, autophagy‐animal, endocrine resistance, chemical carcinogenesis‐receptor activation, thyroid hormone signaling pathway, platelet activation, shigellosis, apelin signaling pathway, fluid shear stress and atherosclerosis, phospholipase D signaling pathway, longevity regulating pathway‐multiple species, pancreatic cancer, EGFR tyrosine kinase inhibitor resistance, PI3K‐Akt signaling pathway, Kaposi sarcoma‐associated herpesvirus infection, Epstein‐Barr virus infection, PD‐L1 expression and PD‐1 checkpoint pathway in cancer (Table S5, Supporting Information). It was evident that these signaling pathways were involved in a variety of viral diseases, suggesting that GGD may have a broad‐spectrum efficacy in treating viral infections. Additionally, we found that phosphoinositide 3‐kinase (PI3K) and protein kinase B (Akt) exhibited strong genetic correlations with the core targets, suggesting that the PI3K‐Akt signaling pathway may be a key mechanism in GGD's treatment of COVID‐19 (Figure S1A,B, Supporting Information). These signaling pathways were accompanied by changes in ROS levels, and since the Nrf2 signaling pathway was a classic pathway for oxidative stress, we also confirmed that Nrf2 and the antioxidant enzyme glutathione reductase (GR) have strong genetic correlations with the core targets, indicating a significant association with ROS (Figure S1C,D, Supporting Information). Finally, we constructed an ingredient─target─disease network, showing the “holistic view” of GanCao─GeGen in treating COVID‐19 (Figure 2F).

The eligible GSE160435 dataset was screened in the Gene Expression Omnibus (GEO) database, and the core target was input into the dataset for verification, and the receiver operating characteristic (ROC) curve was drawn (Figure 2G). ROC curve quantified the diagnostic efficacy of a model by measuring its sensitivity and specificity across a range of threshold values. The area under concentration‐time (AUC) served as a comprehensive metric for the diagnostic accuracy of models. AUC scores above 0.5 had predictive value, above 0.7 indicated good diagnostic value, and above 0.9 indicated high diagnostic value. As shown in Figure 2H, the AUC scores of EGFR, ITGB1, TBK1, TNF, and VEGFA were all above 0.7, and the AUC scores of ITGB1 and TNF were more than 0.9, indicating that the diagnostic value of these targets was higher in the process of COVID‐19. Additionally, the expression of ITGB1 and TNF in the disease group was significantly higher than that in the control group, and TNF, as a key indicator of inflammation, was closely related to COVID‐19.^[^ ^13^ ^]^ Thus, TNF may be the key targets of GanCao─GeGen in the treatment of COVID‐19.

### 2.3 Glabridin (GLA) and Puerarin (PUE) as Candidate Drugs in Treating COVID‐19

It was reported that ACE2,^[^ ^14^ ^]^ 3C‐likeprotease (3CL pro),^[^ ^15^ ^]^ Papain‐like protease (Plp),^[^ ^16^ ^]^ and TNF^[^ ^17^ ^]^ were the key targets in the treatment of COVID‐19, and the binding ability of the key targets to active ingredients in GanCao─GeGen was verified by computer modeling.

**Figure 3:**
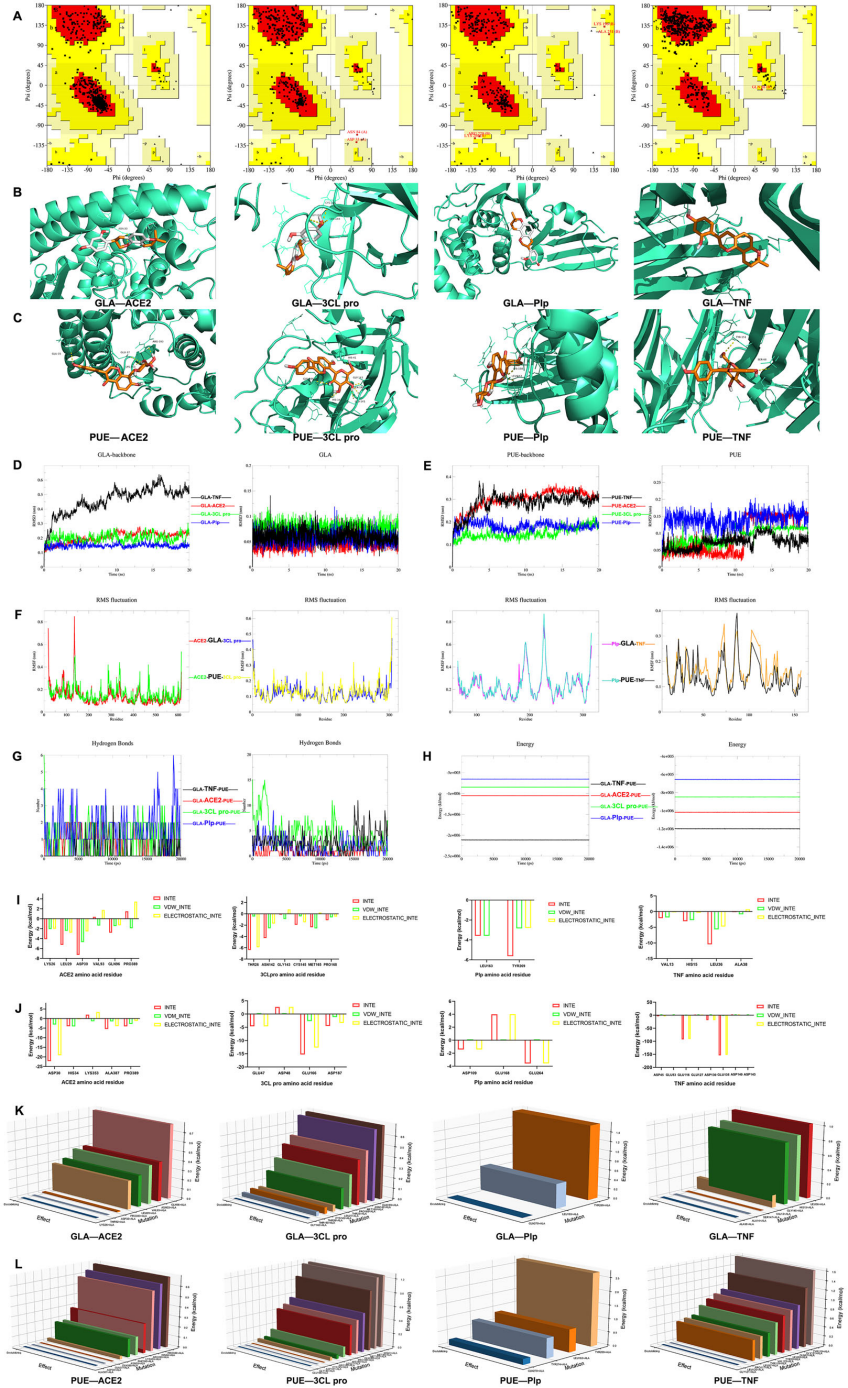
Computer modeling of GLA and PUE with the key targets in treating COVID‐19. A) Evaluation of 3D protein structure models. B) The binding mode of GLA with targets. C) The binding mode of PUE with targets. D) RMSD of system and GLA: the impact of GLA binding on the stability of protein structure in 20 ns simulation time. E) RMSD of system and PUE: the impact of PUE binding on the stability of protein structure in 20 ns simulation time. F) RMSF of protein amino residues: the functionality and dynamic properties of the proteins. G) The number of system's hydrogen bond changes was calculated using the Hbond program in 20 ns simulation time, which was based on geometric criteria for determining hydrogen bonds. It was considered to be a hydrogen bond when the donor‐acceptor distance was less than 3.5 Å and the donor‐acceptor angle was less than 30 degrees. H) System energy: the overall stability of system in 20 ns simulation time. I) GLA: Energy decomposition for amino acid residues of the proteins. J) PUE: Energy decomposition for amino acid residues of the proteins. K) GLA: The key binding sites between the proteins and ingredients. L) PUE: The key binding sites between the proteins and ingredients.

The accuracy of protein crystal structures determined the precision of computer modeling, and the modeling results were assessed through SAVES 6.0.^[^ ^18^ ^]^ The Ramachandran plot was a visualization method that described whether the dihedral angles ψ and φ of amino acid residues in the protein structure were within reasonable regions, and it can also reflect whether the conformation of the protein was rational.^[^ ^19^ ^]^ For ACE2, 93.3% of the amino acids were situated in the most favored regions, 6.7% were found in the allowed regions, with no amino acids present in the generously allowed or disallowed regions. Thus, 100% of the amino acids were within the rational regions. For 3CL pro, 90.6% of the amino acids were in the most favored regions, 8.7% were in the allowed regions, 0.4% were in the generously allowed regions, and 0.4% were in the disallowed regions, leading to 99.6% of the amino acids being within the rational regions. For Plp, 88.0% of the amino acids were in the most favored regions, 10.2% were in the allowed regions, 1.8% were in the generously allowed regions, and none were in the disallowed regions, which meant 100% of the amino acids were within the rational regions. For TNF, 90.2% of the amino acids were in the most favored regions, 9.6% were in the allowed regions, 0.2% were in the generously allowed regions, and again, none were in the disallowed regions, resulting in 100% of the amino acids being within the rational regions (**Figure** **3**A). In addition, we have identified the precise values of ACE2, 3CL pro, Plp, and TNF protein crystal structures to be 96.9336, 96.5517, 91.5612, and 96.4989, respectively, indicating that these 3D protein structure models were reliable (Figure S2, Supporting Information).

The smaller of docking binding value, the more stable of ligand‐receptor binding, and the greater possibility of ligand‐receptor interaction. When the calculated affinity score was less than or equal to −4.25 kcal mol^−1^, it was interpreted as a strong binding interaction between the key targets and the active ingredients. If the affinity score was further reduced to less than or equal to −5 kcal mol^−1^, it was indicated an even tighter binding. Moreover, when the score reached a threshold of less than or equal to −7 kcal mol^−1^, it was deemed that the key targets exhibited the strongest binding affinity with the active ingredients.^[^ ^20^ ^]^ GLA, which can inhibit viral RNA levels and infectious titers of SARS‐CoV‐2 in a dose‐dependent manner,^[^ ^21^ ^]^ and PUE can alleviate inflammation and modulate the host's immune response to improve virus‐induced lung injury.^[^ ^11^ ^]^ GLA and PUE exhibited strong binding affinity to key targets such as ACE2, 3CL pro, Plp, and TNF (**Table** **1**), and they can be well embedded in the active pocket of the targets, indicating that the affinity between the receptor and the ligand was strong (Figure 3B,C).

**Table 1:** Docking scores of GLA and PUE with the key targets.

Ingredient Name	Targets Name (PDB ID)	Scores (kcal mol^−1^)
GLA	ACE2 (6M0J)^[^ ^14^ ^]^	−6.3
	3CL pro (6LU7)^[^ ^23^ ^]^	−8.0
	Plp (4OVZ)^[^ ^24^ ^]^	−7.9
	TNF (2AZ5)^[^ ^25^ ^]^	−7.8
PUE	ACE2 (6M0J)	−6.7
	3CL pro (6LU7)	−7.8
	Plp (4OVZ)	−7.4
	TNF (2AZ5)	−7.6

Given that TNF‐*α* was identified in the preceding systemic network analysis, the HipHop algorithm was employed to valid the binding affinity of GLA and PUE to TNF‐*α*. We identified six active compounds with strong TNF‐*α* inhibitory activity: Japonicone A, AP‐906/41640035, SPD‐304, EJMC‐1, Erythrosine B, and Quercetin,^[^ ^22^ ^]^ which constituted the training set (Figure S3, Supporting Information). This resulted in the generation of ten pharmacophore models, each comprising three feature elements (Table S6, Supporting Information). Evaluation of compounds alignment with pharmacophore models revealed that the FitValue ranking of pharmacophore model 05 closely correlated with the IC_50_ values of active compounds. The compatibility of GLA and PUE with pharmacophore model 05, and their FitValue ranked just below that of AP‐906/41640035, suggesting their potential strong inhibitory activity against TNF‐*α* (Figure S4, Supporting Information).

Considering the molecular docking results, the molecular dynamics simulation of ingredient─key target composite systems was investigated, and the preprocessing procedure was shown in Figures S5 and S6 (Supporting Information). The stability of the system was tested by calculating the RMSD value within 20 ns simulation time, and the results were shown in Figure 3D,E. The results showed that the backbone RMSD of the GLA─key target composite system tended to stabilize after 10 ns, and that of the PUE─key target composite system stabilized after 15 ns, which had good stability without obvious conformational fluctuation. Then, the RMSF value of amino acid skeleton atoms of the key targets were further calculated during the molecular dynamics simulation process, and the results were shown in Figure 3F. The RMSF value indicated that the key targets had greater flexibility and movement intensity during the simulation process. Furthermore, we analyzed the hydrogen bonds of the systems, and found that the main bonding modes of GLA─ACE2, GLA─3CL pro, and GLA─Plp were hydrogen bonds, whereas the binding mode of GLA─TNF does not primarily rely on hydrogen bonds (Figure 3G), which was consistent with the molecular docking results. Also, we found that the main bonding modes of PUE─ACE2, PUE─3CL pro, PUE─Plp, and GLA─TNF were hydrogen bonds, especially for PUE─3CL pro and PUE─TNF. The total energy change of ingredient─key target composite systems was calculated, and the total energy was stable in the simulation process. The stability order of the composite systems for GLA and PUE was as follows: TNF composite systems > ACE2 composite systems > 3CL pro composite systems > Plp composite systems (Figure 3H). Importantly, we also performed energy decomposition analysis on the amino acid residues of key targets, and found that ASP^30^ (ACE2), THR^26^ (3CL pro), TYR^269^ (Plp), and LEU^36^ (TNF) contributed the most affinity to GLA, respectively (Figure 3I). Additionally, ASP^30^ (ACE2), GLU^166^ (3CL pro), GLU^264^ (Plp), and GLU^135^ (TNF) contributed the most affinity to PUE, respectively (Figure 3J). To further identify the key binding sites between the proteins and ingredients, we employed alanine scanning mutagenesis to investigate and mutate the amino acids within the active sites. The results revealed that the key binding sites for GLA with 3CL pro were ASN^142^ and GLN^189^, with ACE2 was GLN^96^, with Plp were LEU^163^ and TYR^269^, and with TNF were GLY^148^, HIS^15^, and LEU^36^ (Figure 3K; Table S7, Supporting Information). For PUE, the key binding sites with 3CL pro were GLN^189^, MET^165^, MET^49^, and HIS^41^, with ACE2 were ARG^393^, HIS^34^, and PRO^389^, with Plp were TYR^274^, LEU^163^, and TYR^269^, and with TNF were TYR^119^, VAL^123^, TYR^59^, LEU^55^, GLN^61^, GLY^121^, and TYR^119^ (Figure 3L; Table S8, Supporting Information).

### 2.4 Study on Anti‐Inflammatory and Antioxidant Effects of GLA

Considering the established anti‐inflammatory and antioxidant properties of PUE from early research, this study did not deem further replication necessary.^[^ ^26^ ^]^ Morphological examination of RAW264.7 cells under normal conditions depicted them as small, round, plump, and well‐defined. In contrast, LPS‐induced inflammation led to a transformation in cellular morphology, characterized by the protrusion of pseudopodia and an enlargement in cell size with blurred contours. After GLA treatment on LPS‐induced M1 macrophages, the number of irregularly shaped cells decreased. (Figure S7A, Supporting Information). We revealed that GLA effectively inhibited the cellular expression of inflammatory cytokines IL‐1*β* and TNF‐*α* by Western blot (Figure S7B‐D, Supporting Information). Also, we used Griess assay to determine the inhibitory effect of GLA on the secretion of nitric oxide (NO) by M1 macrophages. Optical density (OD)value was converted into NO content by the standard curve of sodium nitrite (NaNO_2_), and *R* ^2^ >  0.99 indicated a good linear relationship (2):

(2) $$Y=0.01038X+0.05021\left(R^{2}=0.9986\right)$$

where *X* was OD value, and *Y* was NO content.

NO inhibition rate of GLA was calculated according to the Equation (3):

(3) $$\mathrm{NO}\;\mathrm{inhibition}\;\%\;=\frac{OD_{\mathrm{model}}-OD_{\mathrm{sample}}}{OD_{\mathrm{model}}-OD_{\mathrm{blank}}}\times 100\%$$

The anti‐inflammatory effect of GLA was investigated with the NO inhibition rate as the index. The results showed that the NO content of LPS group was 4.1 µM, and the NO content of 10 µM and 20 µM GLA was 2.7 µM. We found that NO inhibition rate reached 60%, demonstrating that GLA can significantly suppress the secretion of NO by macrophages (Figure S7E‐G, Supporting Information). These findings substantiated the successful construction of an inflammatory cell model and underscored the anti‐inflammatory properties of GLA.

It was reported that the lungs of COVID‐19 patients exhibited severe oxidative stress reactions.^[^ ^27^ ^]^ We employed the DPPH assay to investigate the ability of GLA to scavenge oxygen free radicals in vitro, aiming to elucidate the molecular mechanism of GanCao─GeGen in the prevention and treatment of COVID‐19 from the perspective of oxidative stress. The scavenging effect of GLA on DPPH was expressed as the scavenging rate (*SR*%).

Scavenging rate calculation Equation (4):

(4) $$SR\%\;=\left(A_{0}-\left(A_{i}-A_{j}\right)\right)/A_{0}\times 100\%$$

Sample group (*A_i_*): 100 µL test solution + 100 µL DPPH solution;
Control group (*A_j_*): 100 µL test solution + 100 µL 70% ethanol solution;
Blank group (*A*_0_): 100 µL 70% ethanol solution + 100 µL DPPH solution.

We attempted to determine whether low‐dose GLA could demonstrate free radical scavenging ability. The results showed that GLA at concentrations of 3.125, 6.25 and 12.5 µM exhibited free radical scavenging abilities greater than 20% (Figure S7H, Supporting Information). This indicated that GLA possessed significant free radical scavenging ability in vitro, which appeared to be dose‐dependent.

### 2.5 Preparation and Characterization of GLA‐PUE@gel

**Figure 4:**
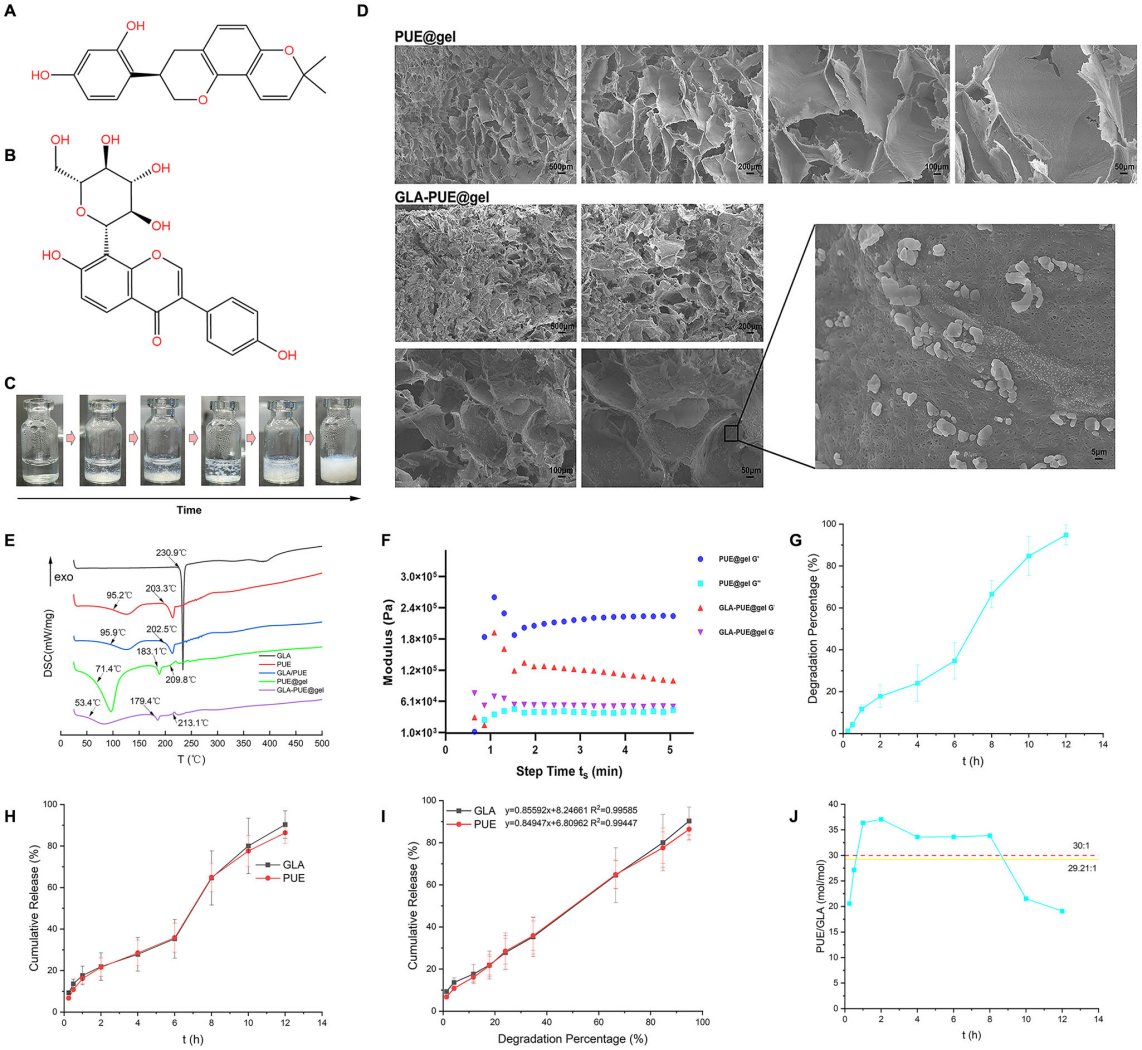
Preparation and characterization of GLA‐PUE@gel. A) Molecular structure of GLA. B) Molecular structure of PUE. C) Actual formation process of GLA‐PUE@gel. D) SEM images of PUE@gel and GLA‐PUE@gel at different magnifications, scale bar: 500, 200, 100, 50 µm. The lower right showed the magnified image, scale bar: 5 µm. E) DSC curves of free GLA, PUE, GLA/PUE, PUE@gel, and GLA‐PUE@gel. F) Dynamic time sweep of PUE@gel and GLA‐PUE@gel at a strain of 1% and a frequency of 1 rad s^−1^. The concentration of GLA in the hydrogel was 3.08 mM, and the concentration of PUE was 92.46 mM, T = 25 °C. G) In vitro degradation profiles of GLA‐PUE@gel, with a concentration of 3.08 mM for GLA and 92.46 mM for PUE in the hydrogel, were conducted by shaking the hydrogel in PBS (pH 7.4) at 37 °C. H) In vitro release profiles of GLA‐PUE@gel, with a concentration of 3.08 mM for GLA and 92.46 mM for PUE in the hydrogel, were conducted by shaking the hydrogel in PBS (pH 7.4) at 37 °C. I) Cumulative release of GLA and PUE was linearly fitted to hydrogel degradation, respectively. J) The variation of the molar ratio of PUE:GLA over time, with the initial molar ratio of PUE:GLA shown in red (30:1) and the actual mean molar ratio of PUE:GLA shown in yellow (29.21:1).

GLA exhibited good antioxidant and anti‐inflammatory properties. However, its application was limited by the strong hydrophobicity (**Figure** **4**A). PUE contained hydrophilic glycosyl groups and hydrophobic aromatic groups (Figure 4B), which enabled a balance between molecular solubility in water and the ability to self‐assemble. A decoction‐mimicking hydrogel of PUE can be synthesized via a heating‐cooling method. Under high supersaturation conditions, the gelation process of PUE began with nucleation, followed by rapid fiber growth radiating from the nucleation centers. As supersaturation increased, the type of fiber growth shifts from a “fibrillar” pattern to a “non‐linear” one, resulting in a denser structure with higher fractal dimensions and enhanced mechanical properties.

In this study, we developed a glabridin‐puerarin self‐assembled hydrogel (GLA‐PUE@gel) with a decoction‐mimicking preparation method. Figure 4C illustrated the macroscopic morphology of the hydrogel, demonstrating its integrity and uniformity. Additionally, scanning electron microscopy (SEM) was employed to characterize PUE@gel and GLA‐PUE@gel, revealing the highly interconnected porous network at different magnifications (Figure 4D). The smoothness of the pore walls and the interconnectivity between pores can be observed, particularly at higher magnifications. Compared to PUE@gel, GLA‐PUE@gel exhibited smaller pore sizes and thicker pore walls under the same magnification. DSC curve was shown in Figure 4E. GLA exhibited an endothermic peak at ≈230.9 °C.^[^ ^28^ ^]^ PUE showed an endothermic peak at ≈95.2 °C due to the loss of crystalline water, and its melting point appeared at 203.3 °C.^[^ ^29^ ^]^ The physical mixture of GLA/PUE displayed an endothermic peak at ≈95.9 °C due to the loss of crystalline water, with a melting point at 202.5 °C, possibly indicating a lower GLA content. The glass transition temperature (*Tg*) of PUE@gel was 71.4 °C, indicating that PUE may exist in an amorphous form after being prepared into a hydrogel.^[^ ^29a^ ^]^ GLA‐PUE@gel exhibited an endothermic peak at ≈53.4 °C, which may be associated with a new amorphous form upon the incorporation of GLA. As reported, the characteristic peaks of GLA in X‐ray Diffraction (XRD) were identified at 2θ values of 16.15° and 17.73°.^[^ ^30^ ^]^ From Figure S8 (Supporting Information), it was found that the characteristic peaks of GLA were present in PUE@gel+GLA, whereas they were absent in GLA‐PUE@gel, indicating the formation of a new co‐gel system. Furthermore, dynamic time sweep tests were performed on PUE@gel and GLA‐PUE@gel at 1% strain and 1 rad s^−1^ frequency. The storage modulus (G′) was found to be significantly higher than the loss modulus (G″), as shown in Figure 4F, confirming the formation of the hydrogel.

Subsequently, we simulated the in vivo degradation process of GLA‐PUE@gel, revealing complete degradation at ≈12 h (Figure 4G). Furthermore, we simulated the in vivo release behaviors of GLA and PUE in GLA‐PUE@gel, showing consistent cumulative release percentages at different time points (Figure 4H). Model fitting indicated that the release behaviors of GLA and PUE followed a Zero‐order model, with 0.45 < *n* < 0.89, and *n* values approaching 0.89 (**Table** **2**). The degradation percentage was linearly fitted with the cumulative GLA and PUE release (*R* ^2^ > 0.99), indicating a strong linear relationship (Figure 4I). This suggested that the release of GLA and PUE was primarily controlled by the degradation of the hydrogel matrix, with drug diffusion playing a secondary role. Importantly, we obtained the concentration ratios of PUE:GLA at different times during the release process and calculated an average of 29.21:1 (mol mol^−1^), closely matching the initial preparation ratio of 30:1 (mol mol^−1^) (Figure 4J). According to DSC and XRD results, it was found that GLA and PUE interacted with each other in GLA‐PUE@gel, forming a new co‐gel system that enabled nearly synchronous release of both compounds. This indicated that the decoction‐mimicking drug delivery system may maintain a stable release ratio in vivo, ensuring therapeutic efficacy.

**Table 2:** Model fitting of GLA and PUE release profiles.

Drug	Release Kinetics model	Equation	*R* ^2^ ^a)^	*n* ^c)^
GLA	Zero‐order	*M_t_*/ *M*_∞_ = *kt* ^b)^	0.96492	0.88468
	First‐order	$\ln(1-M_{t}/M_{\infty })=-kt$	0.94018	
	Higuchi	*M_t_*/ *M*_∞_ = kt^1/2^	0.87179	
	Weibull	$\begin{array}{c} M_{t}/\;M_{\infty }=1-exp.\\ \left(-kt^{b}\right) \end{array}$	*NA*	
	Ritger‐Peppas	*M_t_*/ *M*_∞_ = *kt^n^*	0.94426	
PUE	Zero‐order	*M_t_*/ *M*_∞_ = *kt*	0.97296	0.8674
	First‐order	$\ln(1-M_{t}/M_{\infty })=-kt$	0.95647	
	Higuchi	*M_t_*/ *M*_∞_ = kt^1/2^	0.91847	
	Weibull	$\begin{array}{c} M_{t}/\;M_{\infty }=1-exp.\\ \left(-kt^{b}\right) \end{array}$	*NA*	
	Ritger‐Peppas	*M_t_*/ *M*_∞_ = *kt^n^*	0.96874	

The equations implemented in Origin 2022,

^a)^ the value of *R* ^2^ and *n* obtained after the fitting of models;

^b)^ *M_t_*/*M*_∞_ is the percentage of GLA or PUE released at time *t*;

^c)^ When *n* < 0.45, drug release follows the Fickian diffusion mechanism, indicating that the behavior is controlled by PUE diffusion. When 0.45 < *n* < 0.89, it suggests that the behavior is synergistically controlled by the degradation of the hydrogel matrix and PUE diffusion. When *n* > 0.89, it indicates that the behavior is controlled by the degradation of the hydrogel matrix.

In PBS solution, it was observed that GLA could not dissolve upon heating, whereas GLA‐PUE@gel formed a clear, transparent liquid upon heating (Figure S9A, Supporting Information). This indicated that the hydrogel enhanced the solubility of GLA in water. In a PBS solution containing 0.25% Tween‐80, GLA‐PUE@gel exhibited better solubility compared to PUE@gel (Figure S9B, Supporting Information), indicating that GLA also significantly improved the solubility of PUE@gel. Therefore, this delivery system may have the potential to increase the bioavailability of GLA and PUE@gel. Additionally, the drug release from GLA‐PUE@gel could be sustained for 12 h, demonstrating a good sustained‐release effect.

### 2.6 The Mechanistic Understanding of Self‐Assembled Hydrogel Formation

**Figure 5:**
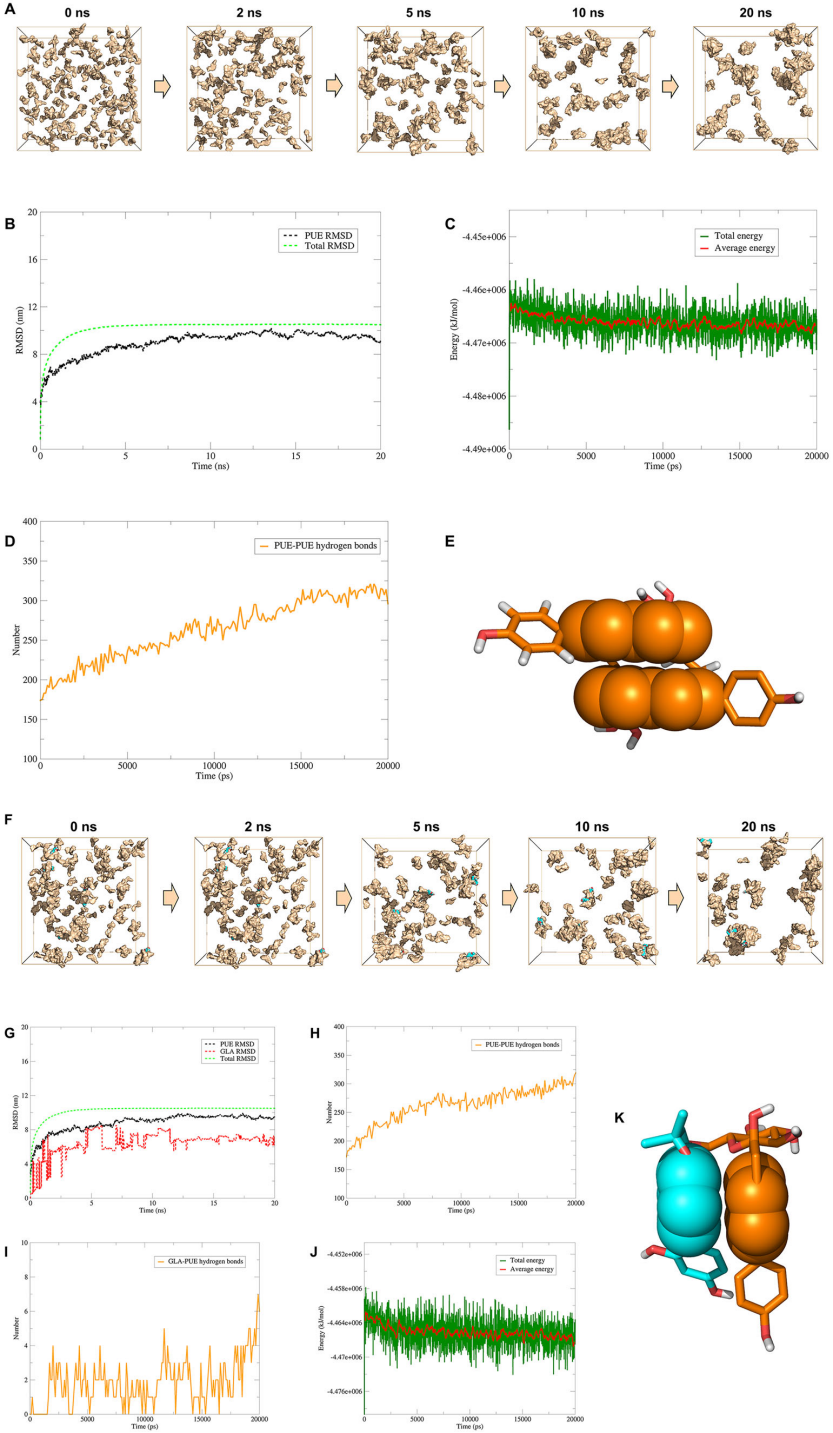
Self‐assembly simulation of PUE@gel and GLA‐PUE@gel. A) Self‐assembly simulation of 180 PUE in aqueous solution, in which wheat is the molecular structure of PUE. Data analysis after simulation: RMSD B), energy C), and hydrogen bonds D). E) The interaction mode between PUE and PUE molecules, in which wheat is the molecular structure of PUE, and the sphere is the site of π‐π stacking. F) Self‐assembly simulation of 180 PUE and 6 GLA in aqueous solution, in which wheat is the molecular structure of PUE, blue is the molecular structure of GLA. Data analysis after simulation: RMSD G), PUE─PUE hydrogen bonds H), GLA─PUE hydrogen bonds I), and energy J). K) The interaction mode between PUE and GLA molecules, in which wheat is the molecular structure of PUE, blue is the molecular structure of GLA, and the sphere is the site of π‐π stacking.

In this section, we followed the preparation concentration of PUE@gel: 92.46 mM (PUE). We constructed a 15 × 15 × 15 nm^3^ SPC water model box and added 180 PUE molecules to investigate the self‐assembly capability under specific conditions (Figure S10, Supporting Information). Through self‐assembly simulation experiments, we gained an understanding of the mechanism behind the formation of PUE@gel: 180 PUE gradually aggregated within 20 ns, exhibiting a porous structure (**Figure** **5**A). Furthermore, the RMSD and energy of PUE and its system stabilized during the formation of the self‐assembled hydrogel (Figure 5B,C), with hydrogen bonding and π‐π stacking being among the primary driving forces (Figure 5D,E).

Building upon previous findings, it was established that GLA and PUE were key active ingredients in GGD, displaying significant synergistic anti‐inflammatory and antioxidant effects. However, the mechanism of action remained unclear. Following the preparation ratio of GLA‐PUE@gel: 3.08 mM (GLA) and 92.46 mM (PUE), we constructed a cubic box with a side length of 15 nm and formed an SPC water model system. We then added 180 PUE and 6 GLA molecules to the system (Figure S11, Supporting Information), calculating the quantities of ingredients based on the following Equation (5):

(5) $$N=C\times V\times N_{A}$$

where *N* is the amount of substance in moles, *C* is the concentration of the substance in solution, *V* is the volume of the solution, *N_A_* is Avogadro constant.

Through self‐assembly simulation experiments, we gained an understanding of the mechanism behind the formation of GLA‐PUE@gel: 6 GLA and 180 PUE gradually aggregated within 20 ns, exhibiting a porous structure (Figure 5F). RMSD of the system, PUE, and GLA reached a steady state after ≈12 ns (Figure 5G). After 10 ns, the amount of hydrogen bonds between PUE─PUE tended to be in a stable state, which may be related to the participation of GLA (Figure 5H). GLA was enveloped within PUE@gel through hydrogen bonding and π‐π stacking, forming GLA‐PUE@gel (Figure 5I,K). Additionally, the energy of the system ranged from −4.46 to −4.47 × 10^−6^ kJ mol^−1^, indicating that GLA cannot significantly affect the overall energy change of the system (Figure 5J). Docking results revealed that the binding affinity of PUE─PUE was slightly stronger than that of GLA─PUE (Figure 5E,K; Tables S9 and S10, Supporting Information), both being less than −5 kcal mol^−1^, indicating that the interaction force between individual molecules was weak, while the spatial structure of multiple molecules was the main contributor for the stability of the system.

### 2.7 Study on the Anti‐Inflammatory and Antioxidant Effects of GLA‐PUE@gel

**Figure 6:**
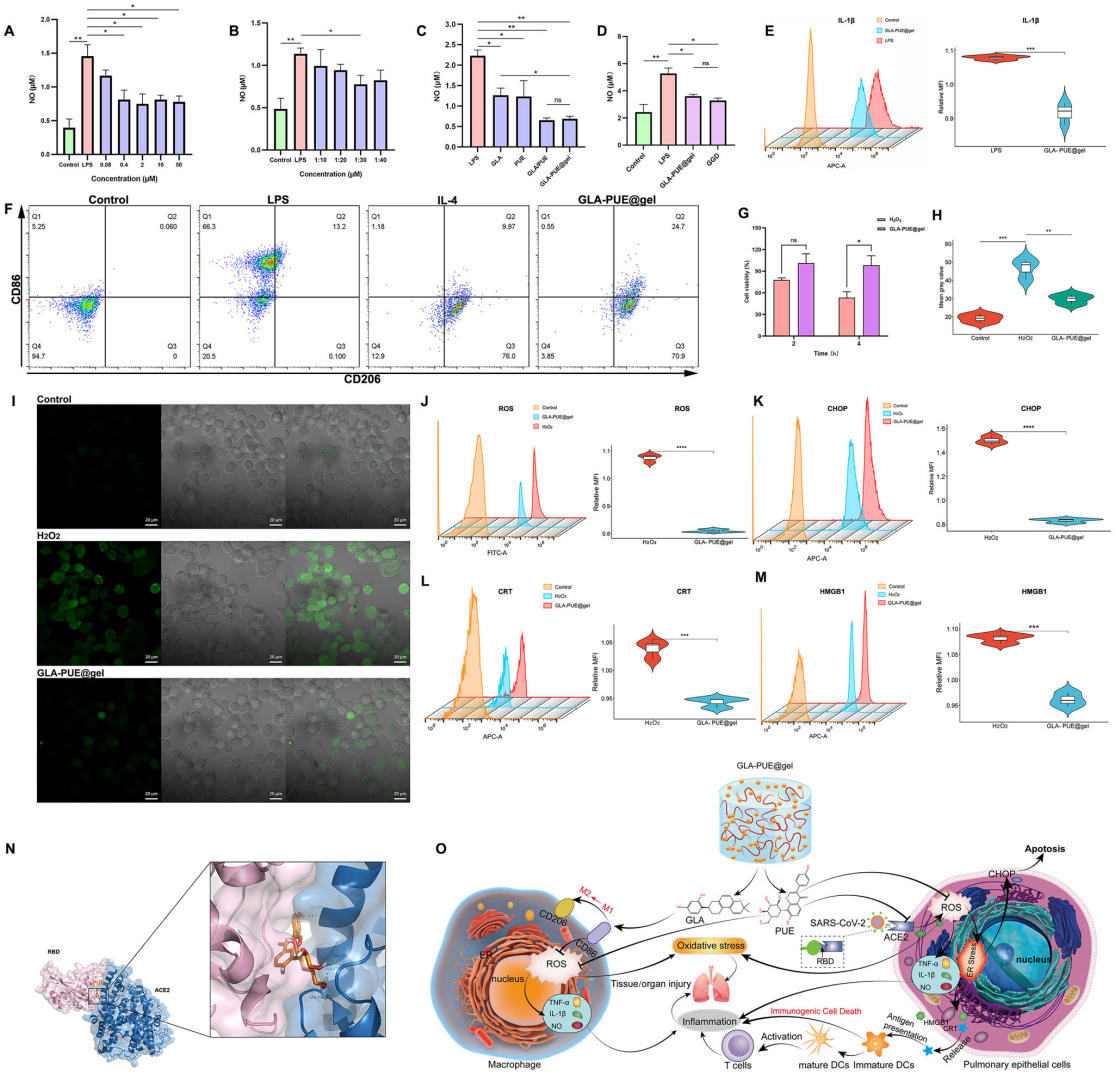
GLA‐PUE@gel alleviated virus‐induced inflammation and oxidative stress. A) Effective concentration of GLA in inhibiting NO was explored in the supernatant of RAW264.7 cells. B) The synergistic proportion of GLA/PUE in NO inhibition was explored in the supernatant of RAW264.7 cells. C) The inhibitory effect of GLA‐PUE@gel on NO in the supernatant of RAW264.7 cells was compared to that of free drugs. D) The inhibitory effect of GLA‐PUE@gel on NO in the supernatant of RAW264.7 cells was compared to that of GGD. E) Flow cytometry was used to evaluate the inhibitory effect of GLA‐PUE@gel on IL‐1*β* concentration in RAW264.7 cells compared to the LPS group. F) GLA‐PUE@gel altered the ratio of the surface markers CD86/CD206 on THP‐1 cells. G) A549 cells viability was assessed after exposure to 400 µM H_2_O_2_ for 2 and 4 h. H) The average fluorescence intensity corresponding to ROS in each group of A549 cells was measured by CLSM. I) CLSM was utilized to assess the inhibitory effect of GLA‐PUE@gel on ROS in A549 cells compared to the H_2_O_2_ group, scale bar: 20 µm. Flow cytometry was used to detect the inhibitory effect of GLA‐PUE@gel on ROS J), CHOP K), CRT L), and HMGB1 M) in A549 cells compared to the H_2_O_2_ group. N) Molecular model of competitive interference of PUE with ACE2 binding to RBD. O) GLA‐PUE@gel prevented SARS‐CoV‐2 from entering host cells, inhibited intracellular endoplasmic reticulum stress, and reprogramed macrophages to alleviate inflammation and oxidative stress for COVID‐19 therapy. n = 3, *p < 0.05, **p < 0.01, ***p < 0.001, and ****p < 0.0001.

To determine the synergistic anti‐inflammatory proportion of GLA/PUE, we employed the Griess assay to measure the changes in NO levels in macrophage culture supernatants. First, we confirmed that GLA exhibited significant anti‐inflammatory effects at doses above 0.4 µM (**Figure** **6**A). Subsequently, we established concentration gradients of GLA:PUE ratios at 1:10, 1:20, 1:30, and 1:40, while ensuring that GLA and PUE remained within safe dosage ranges. Notably, the ratio of GLA to PUE at 1:30 was found to be particularly effective in eliciting a pronounced anti‐inflammatory response (Figure 6B). Based on this finding, we prepared GLA‐PUE@gel and compared its anti‐inflammatory activity with free drugs and GGD. We found that GLA‐PUE@gel showed significant differences in anti‐inflammatory activity compared to GLA, PUE, and GGD, but no significant difference compared to the GLA/PUE, indicating a rational synergistic proportion (Figure 6C,D). Furthermore, we investigated the impact of GLA‐PUE@gel on the levels of IL‐1*β* in macrophages and observed a significant reduction in IL‐1*β* levels compared to the LPS group (Figure 6E). Changes in surface markers on macrophages were important indicators for assessing inflammatory and non‐inflammatory states. We induced an inflammatory model (M1) using LPS and an anti‐inflammatory model (M2) using IL‐4. After treating M1 macrophages with GLA‐PUE@gel for 24 h, we observed a significant decrease in the proportion of CD86‐positive macrophages and an increase in the proportion of CD206‐positive macrophages (Figure 6F). This indicated that GLA‐PUE@gel can reprogram the inflammatory state of macrophages into a non‐inflammatory state, thereby inhibiting the release of inflammatory factors.

To determine the antioxidant effect of GLA‐PUE@gel, we constructed a cellular oxidative stress model using H_2_O_2_. Initially, we treated human alveolar basal epithelial cells (A549) with 400 µM H_2_O_2_ and measured cell viability after 2 h and 4 h. It was observed that cell viability was reduced to ≈50% after a 4‐h exposure to 400 µM H_2_O_2_, indicating successful induction of oxidative stress model. However, GLA‐PUE@gel significantly attenuated oxidative stress damage (Figure 6G). Furthermore, we assessed the impact of GLA‐PUE@gel on reactive oxygen species (ROS) levels using an ELISA assay kit. H_2_DCFDA was inherently non‐fluorescent and can freely penetrate cell membranes. Once inside the cell, it was hydrolyzed by cellular esterases to form DCFH, which was not membrane‐permeable, thus allowing the probe to be easily loaded into the cells. Subsequently, non‐fluorescent DCFH can be oxidized by intracellular ROS to form fluorescent DCF. Immunofluorescence (Figure 6H,I) and flow cytometry (Figure 6J) were utilized to detect the fluorescence intensity of DCF and assess intracellular ROS levels. The results demonstrated that GLA‐PUE@gel significantly reduced intracellular ROS levels, indicating remarkable antioxidant activity.

Excessive ROS can disrupt the normal function of the endoplasmic reticulum (ER), such as protein folding and quality control processes, thereby inducing ER stress.^[^ ^31^ ^]^ Further investigations revealed that GLA‐PUE@gel activated the expression of C/EBP homologous protein (CHOP), which in turn inhibited apoptosis of cells (Figure 6K). Under ER stress conditions, calreticulin (CRT) can translocate from the ER to the cell membrane, thereby inducing immunogenic cell death (ICD), promoting T cells to produce large amounts of cytokines, and exacerbating inflammation. GLA‐PUE@gel effectively suppressed this process, suggesting its potential in alleviating oxidative stress damage through inhibition of the ER stress pathway (Figure 6L). Moreover, overexpression of high mobility group box 1 protein (HMGB1) can induce ER stress in A549 cells, and GLA‐PUE@gel can significantly reduce the production of HMGB1 (Figure 6M).^[^ ^32^ ^]^

In previous studies, it was found that PUE can inhibit the production of inflammatory factors to reduce inflammation and oxidative stress damage,^[^ ^33^ ^]^ while GLA can reprogram M1 macrophages into M2 macrophages, thereby exerting anti‐inflammatory and antioxidant effects. ACE2 was a zinc metalloproteinase, which belonged to type I transmembrane protein.^[^ ^34^ ^]^ It was originally thought to be expressed in the heart, kidney and testis, and later found to be widely expressed in the lung, brain and digestive tract.^[^ ^35^ ^]^ Studies have found that ACE2 was a key membrane receptor for SARS‐CoV‐2 entering host cells and had a strong binding ability to SARS‐CoV‐2 spike protein receptor binding domain (RBD).^[^ ^8^ ^]^ It has been reported that PUE can competitively inhibit the binding of ACE2 and RBD, thereby preventing SARS‐CoV‐2 from entering host cells.^[^ ^10^ ^]^ Using computer simulation technology, we discovered that PUE can competitively occupy the active binding sites of ACE2 and RBD (GLU^35^, GLU^37^, LYS^353^, ARG^393^), thus preventing SARS‐CoV‐2 from binding to host cells (Figure 6N; Table S11, Supporting Information). The synergistic anti‐inflammatory and antioxidant effects of GLA and PUE have been demonstrated to markedly reduce the pathological damage induced by SARS‐CoV‐2. This insight shed light on the mechanism by which GLA‐PUE@gel exerted its therapeutic efficacy, highlighting its potential as a novel treatment approach (Figure 6O).

### 2.8 GLA‐PUE@gel Alleviates Acute Lung Injury (ALI) in Mice

**Figure 7:**
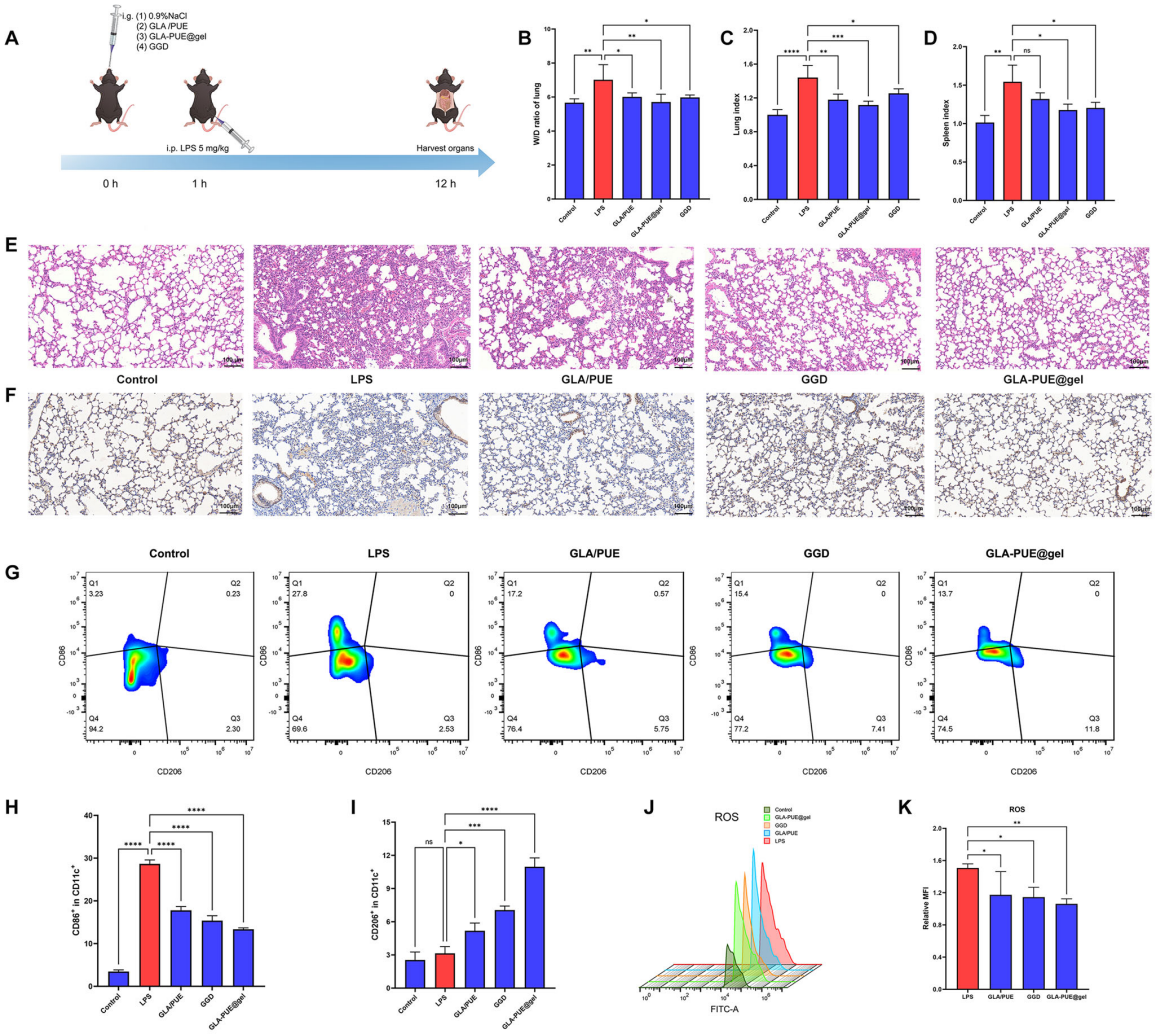
GLA‐PUE@gel ameliorates ALI through anti‐inflammatory and antioxidant. A) Administration protocol for in vivo experiments. B) Pulmonary edema index (lung wet weight/lung dry weight). C) Lung index (lung wet weight/body weight). D) Spleen index (spleen weight/body weight). E) H&E staining of lung tissue (scale bar: 100 µm). F) Immunohistochemical analysis of TNF‐*α* in lung tissues (scale bar: 100 µm). Flow cytometry was used to evaluate changes in the proportions of CD86^+^/CD206^+^ macrophages G) and ROS levels J) in lung tissues, with quantitative analysis shown in H), I), and K). n = 6, *p < 0.05, **p < 0.01, ***p < 0.001, and ****p < 0.0001.

To evaluate the anti‐inflammatory and antioxidant stress capabilities of GLA‐PUE@gel, we employed a mouse model of ALI, as illustrated in **Figure** **7**A (the details are provided in the Experimental Section). Macroscopic examination revealed that the lungs of the ALI group exhibited significant swelling with a red‐and‐white mottled appearance (Figure S12A, Supporting Information). Additionally, the spleens of the ALI group showed marked enlargement and darkening in color, whereas GLA‐PUE@gel treatment significantly ameliorated these pathological changes (Figure S12B, Supporting Information). To quantitatively assess pulmonary edema, lungs were dried at 80 °C for 48 h to obtain dry weights, and the wet‐to‐dry weight ratio was calculated. Furthermore, lung and spleen indices were determined to evaluate the degree of lung consolidation and systemic immune function, respectively. The results demonstrated that GLA‐PUE@gel significantly alleviated pulmonary edema and reduced both lung and spleen indices (Figure 7B–D), indicating its protective effects against lung consolidation and its ability to modulate immune function. Notably, the therapeutic efficacy of 50 mg kg^−1^ GLA‐PUE@gel surpassed that of a clinically equivalent dose of GGD, particularly in reducing the lung index, where a statistically significant difference was observed. Histopathological analysis via H&E staining revealed that normal lung tissues exhibited thin and intact alveolar walls, clear alveolar spaces without exudate, uniform interstitium without thickening or inflammatory cell infiltration, and no significant pathological alterations. In contrast, ALI lung tissues displayed markedly thickened alveolar walls, collapsed or fused alveolar spaces, and abundant pink proteinaceous exudate and red blood cells within the alveolar cavities. Additionally, extensive infiltration of inflammatory cells, such as neutrophils and macrophages, was observed in both the alveolar spaces and interstitium. The interstitium was significantly thickened, accompanied by edema and inflammatory cell aggregation, resulting in disrupted lung architecture and characteristic features of acute inflammation and injury. However, GLA‐PUE@gel effectively ameliorated these histopathological changes in ALI (Figure 7E).

To further validate the observed experimental phenomena, we assessed inflammation and oxidative stress‐related markers in lung tissues from each group. Immunohistochemical analysis of TNF‐*α* expression revealed low levels in normal lung tissues, whereas ALI lungs exhibited significantly elevated TNF‐*α* expression, reflecting a robust inflammatory response and the pivotal role of TNF‐*α* in lung injury. Notably, GLA‐PUE@gel treatment markedly reduced TNF‐*α* levels in lung tissues (Figure 7F). Additionally, flow cytometry was employed to evaluate changes in the proportions of CD86^+^ and CD206^+^ macrophages in lung tissues. The percentage of CD86^+^ macrophages in ALI lungs was significantly higher than in the normal group, reaching 27.8%. In contrast, treatment groups, particularly the GLA‐PUE@gel group, significantly reduced the proportion of CD86^+^ macrophages while increasing the proportion of CD206^+^ macrophages (Figure 7G–I). Finally, flow cytometry was used to assess changes in ROS levels in lung tissues. The results demonstrated that GLA‐PUE@gel treatment significantly reduced ROS levels in lung tissues (Figure 7J,K).

### 2.9 Preliminary Assessment of Drug‐Like Properties and Biological Safety

**Figure 8:**
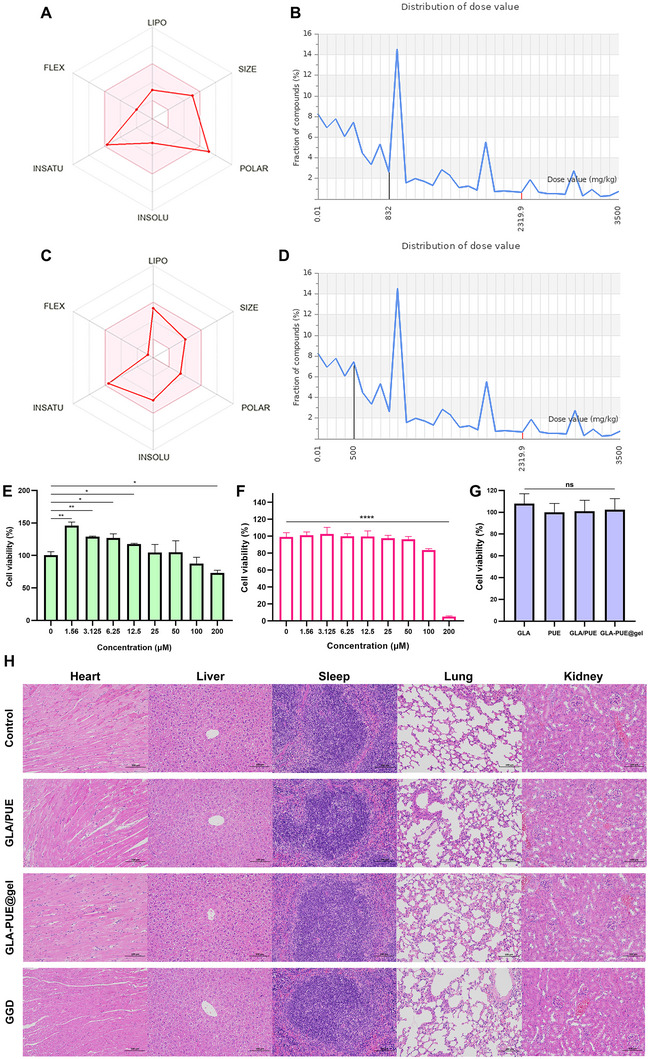
Preliminary assessment of drug‐like properties and biological safety. A) Drug‐like properties of PUE. B) Toxicity prediction of PUE in animals (LD_50_ = 832 mg kg^−1^). C) Drug‐like properties of GLA. D) Toxicity prediction of GLA in animals (LD_50_ = 500 mg kg^−1^). Cytotoxicity of PUE E), GLA F), and GLA‐PUE@gel G). H) H&E staining of various organs across different treatment groups: Evaluation of drug safety in vivo (scale bar: 100 µm). n = 6, *p < 0.05, **p < 0.01, and ****p < 0.0001.

In the drug‐likeness evaluation of GLA and PUE, we found that PUE had a low GI absorption, indicating that the free drug may not be suitable for direct oral administration; PUE had poor BBB permeability, suggesting that the free drug may not be suitable for central nervous system diseases; PUE was not a P‐gp substrate, indicating that it may not be recognized and pumped out of cells by P‐gp; PUE was not a CYP1A2 inhibitor, CYP2C19 inhibitor, CYP2C9 inhibitor, CYP2D6 inhibitor, or CYP3A4 inhibitor, suggesting that it may not affect drug metabolism inside cells; PUE complied with Lipinski and Ghose rules, indicating a certain degree of drug‐likeness; PUE had an LD_50_ of 832 mg kg^−1^ in animals with no significant toxicity to the body, suggesting high safety (**Figure** **8**A,B; Table S12, Supporting Information).

GLA had high GI absorption, indicating that the free drug may be suitable for oral administration; GLA had high BBB permeability, suggesting that the free drug may be suitable for central nervous system diseases; GLA was a P‐gp substrate, indicating that it may be recognized and pumped out of cells by P‐gp; GLA was a CYP1A2 inhibitor, CYP2C19 inhibitor, CYP2C9 inhibitor, CYP2D6 inhibitor, and CYP3A4 inhibitor, suggesting that it may affect drug metabolism inside cells; GLA complied with Lipinski, Ghose, Veber, Egan, and Muegge rules, indicating good drug‐likeness; GLA had an LD_50_ of 500 mg kg^−1^ in animals with no significant toxicity to the body, indicating high safety (Figure 8C,D; Table S13, Supporting Information).

Safety is a necessary prerequisite for drug development, and we used CCK‐8 assay to assess the cytotoxicity of GLA, PUE, GLA/PUE, and GLA‐PUE@gel on macrophage proliferation. When PUE was below 100 µM, it exhibited negligible toxicity to RAW264.7 cells, indicating a high level of biosafety for PUE. Conversely, PUE significantly promoted the proliferation of RAW264.7 cells at concentrations below 12.5 µM and demonstrated a positive effect on counteracting viral invasion (Figure 8E). Furthermore, GLA showed no significant cytotoxicity to RAW264.7 cells at concentrations below 100 µM, confirming its high biological safety (Figure 8F). Finally, we evaluated the cell safety of 20 µM GLA‐PUE@gel (based on GLA) and found no significant difference compared to free drugs, indicating the high biological safety of this drug delivery system (Figure 8G).

Finally, we conducted an in vivo safety evaluation of GLA‐PUE@gel and found no significant pathological changes in the heart, liver, spleen, lungs, or kidneys of mice in the treatment groups compared to the Control group (Figure 8H). These results indicate that GLA/PUE, GGD, and GLA‐PUE@gel exhibit favorable safety at the effective doses.

## 3 Discussion

Through a bibliometric analysis of recent literature, it has been found that TCM has made significant contributions to treat COVID‐19, with keywords such as “NLRP3 inflammasome”, “macrophage polarization”, “injury”, and “antiviral drug” being prominent (Figure 1F). Specifically, GGD has shown good efficacy against various pandemic respiratory diseases, but the molecular mechanism remains unclear.^[^ ^6^, ^7^ ^]^ While GGD has been reported to show antiviral effects, its primary pharmacological role was not as an antiviral agent but rather as a mitigator of severe disease progression.^[^ ^6^ ^]^ Therefore, this study focused on inflammation and oxidative stress to reveal the mechanisms of GGD in the application of respiratory pandemics, and develop common therapeutic agents capable of reducing pathological damage.

Clinically, we have found that GGD can significantly reduce the severity and mortality rates of viral diseases.^[^ ^4^, ^5^, ^6^, ^7^ ^]^ Since GGD was traditionally prepared by decoction to create a medicinal soup, it was observed that a gel‐like precipitate formed upon cooling after decoction. This suggested that there may be some special ingredients in GGD that can form a hydrogel after heating and cooling. Due to the complex composition of GGD, coupled with challenges such as slow onset, long cycle, and difficulty in controlling quality and unclear active ingredients and mechanisms, we aimed to identify the main effective ingredients from GGD based on the theory of TCM compatibility. We focused on the pivotal medicinal combination from GGD, GanCao─GeGen, to obtain mechanistic understanding. We used systems pharmacology to predict the molecular mechanisms of GanCao─GeGen, followed by molecular docking and transcriptome sequencing to identify the main active ingredients GLA and PUE, and the key target TNF. TNF was known as a critical marker of inflammation and was highly expressed in patients with COVID‐19, supporting the clinical application of GGD in reducing the pathological damage. Pharmacological evaluations revealed that GLA and PUE, derived from GanCao and GeGen, exhibited significant anti‐inflammatory and antioxidant properties. Additionally, PUE, one of the main active ingredients in GeGen, has shown unique advantages in the treatment of COVID‐19. It can inhibit the self‐replication of SARS‐CoV‐2 by suppressing the activity of 3CL pro,^[^ ^15^ ^]^ and intervene in the invasion of SARS‐CoV‐2 into host cells by preventing the binding of the virus's RBD to the ACE2 on host cell membranes.^[^ ^10^ ^]^ Interestingly, we found that PUE can self‐assemble into a 3D hydrogel structure, PUE@gel, after being heated to 85 °C and then cooled to 25 °C (Figure 4C).^[^ ^36^ ^]^ Notably, GGD tended to form a gel‐like precipitate following decoction. Therefore, we speculated that GLA and PUE may be an effective medicinal combination for treating SARS‐CoV‐2‐induced inflammation and oxidative stress.

Initially, we determined the optimal synergistic anti‐inflammatory ratio of GLA to PUE, discovering that a molar ratio of 1:30 significantly amplified their combined anti‐inflammatory efficacy. Moreover, GLA at doses above 0.4 µM exhibited significant anti‐inflammatory effects, with 2 µM GLA showing comparable anti‐inflammatory effects to 60 µM PUE, indicating the potent anti‐inflammatory efficacy of GLA. Using this ratio as a foundation, we constructed a decoction‐mimicking drug delivery system, GLA‐PUE@gel, and found that the degradation profile of the hydrogel matrix aligned closely with the drug release profile (Figure 4H), suggesting that drug release was primarily driven by the degradation of the hydrogel matrix. Throughout the release period, the ratio of GLA:PUE remained consistently ≈1:30 (mol mol^−1^) (Figure 4J), confirming the rational design of this delivery system. Furthermore, we constructed a computer model of the self‐assembled hydrogel and found that hydrogen bonding and π‐π stacking interactions of PUE─PUE and GLA─PUE were the main driving forces for hydrogel formation (Figure 5). Through DSC and XRD experiments, it was discovered that GLA interacted with PUE, forming a new co‐gel system for stable drug release (Figure 4E; Figure S8, Supporting Information). Pharmacological evaluations demonstrated that GLA‐PUE@gel exhibited strong anti‐inflammatory and antioxidant properties, comparable to GGD at the same dose (based on GLA), and showed promising potential in alleviating virus‐induced pathological damage (Figure 6). It was capable of converting pro‐inflammatory M1 macrophages into anti‐inflammatory M2 macrophages, which may be an important mechanism for countering oxidative stress and inflammatory responses. We also found that an important reason why GGD was applied to treat COVID‐19, was that PUE can competitively occupy the active sites of ACE2 on host cells, thereby preventing the virus from invading the host cells. Additionally, we have discovered that GLA‐PUE@gel's ability to prevent exacerbated inflammation may be related to inhibiting ER stress. It inhibited CHOP expression to prevent cell apoptosis and suppressed CRT expression to interfere with ICD, thereby inhibiting the release of inflammatory cytokines from T cells.

Furthermore, we investigated the anti‐inflammatory and antioxidant stress capabilities of GLA‐PUE@gel in an ALI mouse model. In fact, the lungs of ALI mice exhibited significant swelling and a red‐and‐white mottled appearance, attributed to increased vascular permeability induced by LPS, leading to the leakage of fluid and proteins into alveolar spaces and interstitium, resulting in pulmonary edema.^[^ ^37^ ^]^ In severe cases, LPS‐induced lung injury was accompanied by alveolar hemorrhage and fibrin deposition, contributing to the heterogeneous pale or red‐and‐white appearance of the lungs (Figure S12A, Supporting Information).^[^ ^38^ ^]^ Additionally, the spleens of ALI mice showed marked enlargement and darkening in color. However, treatment with GLA‐PUE@gel prevented lung and spleen swelling, restoring the normal appearance (Figure S12B, Supporting Information), indicating that GLA‐PUE@gel mitigates LPS‐induced severe lung injury. This aligns with the clinical efficacy of GGD in reducing the incidence of severe conditions in patients. Notably, 50 mg kg^−1^ GLA‐PUE@gel demonstrated superior efficacy in improving the lung index compared to a clinically equivalent dose of GGD, likely due to the complex composition, lower active ingredient content, and poor bioavailability of GGD. Pharmacological analysis of lung tissues revealed that GLA‐PUE@gel treatment suppressed both LPS‐induced inflammatory and oxidative stress responses, consistent with in vitro findings. Moreover, the proportion of CD206^+^ macrophages significantly increased in the GLA‐PUE@gel group, indicating a rise in M2 macrophage polarization, which aligns with its anti‐inflammatory effects.

Here, we found that PUE had a high biosafety threshold, making it suitable as a carrier material in drug delivery (Figure 8). Leveraging the synergistic anti‐inflammatory and antioxidant properties of GLA and PUE, we utilized PUE as a carrier material to construct a hydrogel encapsulating GLA, namely GLA‐PUE@gel. This study successfully simplified the complex composition of GGD by isolating its active ingredients, PUE and GLA, and developed a safe and effective drug delivery system. This approach holds significant potential for reducing the incidence of severe lung injury in clinical settings.

## 4 Experimental Section

### Materials

Glabridin (GLA, ≥ 98%, Herbpurify, China), puerarin (PUE, ≥ 98%, Aladdin, China), Dulbecco's Modified Eagle's Medium (DMEM, Keygen BioTECN, China), Roswell Park Memorial Institute 1640 Medium (RPMI‐1640, Keygen BioTECN, China), Fetal Bovine Serum (FBS, Sigma, Germany), H_2_DCFDA (Biosharp, China), confocal dishes (Biosharp, China), LPS (Solarbio, China), IL‐4 (Novoprotein, China), PMA (Solarbio, China), DPPH (Macklin, China), Triton X‐100 (Sangon Biotech, China), H_2_O_2_ (Nanjing Chemical Reagent, China), IL‐1 beta Polyclonal antibody (Proteintech, China), HMGB1 Polyclonal antibody (Proteintech, China), GADD153 Polyclonal antibody (CHOP, Proteintech, China), calreticulin Polyclonal antibody (Proteintech, China), CoraLite647‐conjugated Mouse Anti‐Heavy Chain of Rabbit IgG (Proteintech, China), PE Anti‐Mouse CD86 Antibody (Elabscience, China), APC Anti‐Mouse CD206/MMR Antibody (Elabscience, China), Protease Inhibitor (Beyotime, China), SDS‐PAGE Protein Sample Loading Buffer (5×) (Beyotime, China), Micro BCA Protein Assay Kit (Beyotime, China), MeilunGel Precast PAGE Gel (Meilun, China), Colour Mixed Protein Marker (Meilun, China).

### Cell Culture

Human monocytic leukemia cell line THP‐1, Human alveolar basal epithelial cell line A549, and the murine stable cell line RAW264.7 were purchased from the National Collection of Authenticated Cell Cultures, China. Cells were cultured in RPMI‐1640 (THP‐1) or DMEM (A549 & RAW264.7) medium containing 10% (v/v) FBS and 1% (v/v) penicillin‐streptomycin mixture at 37 °C, 5% CO_2_. The culture medium was replaced with fresh medium every other day.

### Animals

Male C57BL/6J mice (8 weeks, 22 ± 2 g) were purchased from Hangzhou Ziyuan Biological Technology Co., Itd. All mice were housed in the specific pathogen‐free, temperature‐controlled facility at the Animal Experimental Center of China Pharmaceutical University, Nanjing, P. R. China. The animal experiments were carried out according to the protocol approved by the Ministry of Health in the People's Republic of P. R. China. Minimization of experimental animal numbers and experimental protocols were approved by the Ethics Committee of China Pharmaceutical University, Nanjing, P. R. China (approval number: 2025‐01‐044).

### Construction and Therapeutic Interventions of ALI Models

To evaluate the preventive and therapeutic effects of GLA‐PUE@gel on ALI, an LPS‐induced ALI model was established in C57BL/6J mice. The mice were randomly divided into five groups (n = 6): Control, LPS, GLA/PUE, GGD, and GLA‐PUE@gel. All treatment groups (50 mg kg^−1^ GLA/PUE, 50 mg kg^−1^ GLA‐PUE@gel, and a clinically equivalent dose of GGD) were administered via intragastric (i.g.) gavage. One hour later, ALI was induced by intraperitoneal (i.p.) injection of 5 mg kg^−1^ LPS. After 12 h, the mice were euthanized, and lung tissues were collected for the analysis of relevant histological indicators.

### CiteSpace Bibliometric Analysis

The relevant literature of TCM against COVID‐19 was searched in WOS. To guarantee the authenticity and reliability of the data, the literature found within the Web of Science Core Collection database was meticulously analyzed. The search query used was (TS = (COVID‐19)) AND TS = (Traditional Chinese Medicine), indexed in SCI‐EXPANDED, CPCI‐S, CPCI‐SSH, BKCI‐S, BKCI‐SSH, ESCI, CCR‐ EXPANDED, IC. The literature with the time span of 2019.12‐2024.1 was searched. In addition, the languages and types of these documents were limited to ENGLISH and ARTICLE, respectively. After searching with the above methods, a bibliometric analysis of the obtained literature was conducted.

CiteSpace 6.1.6 software was used to convert WOS data and data processing. First, the selection time was from 2019 to 2024, and the time slice was 1 year. Then, Keyword, Author, Institution, Country, Topic, Burstness of the documents were analyzed respectively, and the others were selected as defaults.

### System Biological Network Analysis

System biological network was an interdisciplinary science that studies the molecular mechanisms of drugs. It employs interactive networks and applied algorithms to construct biological information networks, aiming to elucidate the complex interaction between drugs and targets and signaling pathways from a comprehensive perspective.

First, all compounds in GanCao─GeGen were collected and screened from the TCMSP 2.3 database (https://tcmsp‐e.com/)^[^ ^39^ ^]^ to identify potential active ingredients (OB > 30%, DL > 0.18). Then, the ingredient‐targets were predicted by the Swiss Target Prediction database (http://old.swisstargetprediction.ch/),^[^ ^40^ ^]^ and the ingredient‐targets with possibility greater than 0 were retained. In addition, disease‐targets related to COVID‐19 were searched in Therapeutic Target Database (TTD) (http://db.idrblab.net/ttd/),^[^ ^41^ ^]^ GeneCards 5.20 (https://www.genecards.org/),^[^ ^42^ ^]^ OMIM (https://omim.org/)^[^ ^43^ ^]^ databases. Finally, the symbols of targets were standardized through the Uniprot database (https://www.uniprot.org/).^[^ ^44^ ^]^ Using the Jevnn online platform (http://jvenn.toulouse.inra.fr/)^[^ ^45^ ^]^ to intersect and visualize the ingredient‐targets and disease‐targets, the potential targets of GanCao─GeGen against COVID‐19 were obtained. The STRING 12.0 platform (https://www.string‐db.org/)^[^ ^46^ ^]^ was used to analyze the interaction between potential targets, the species was set as “Homo sapiens”, and the results were visualized by Cytoscape 3.10.2 software.^[^ ^47^ ^]^ The potential targets were uploaded to the Metascape 3.5 (https://metascape.org/)^[^ ^48^ ^]^ platform to obtain biological function and signaling pathways, and the parameters were set as follows: analysis as species: Homo sapiens, min overlap: 3, *p* value cutoff: 0.01, min enrichment: 1.5.

### GEO Microarray Data Processing

GEO datasets (http://www.ncbi.nlm.nih.gov/geo/)^[^ ^49^ ^]^ were used to find the gene chips related to COVID‐19, and Graphpad Prism 8.0.2 software was used for gene expression differential analysis. ROC curve was constructed for the core targets, and AUC curve was used to evaluate the diagnostic value of the core targets on COVID‐19.

### Computer Modeling and High‐Throughput Screening

First, the crystal structures of core targets were downloaded from the RCSB Protein Data Bank (https://www.rcsb.org/),^[^ ^50^ ^]^ SAVES 6.0 (https://saves.mbi.ucla.edu/)^[^ ^18^ ^]^ was used to evaluate the accuracy of protein crystal structure, and the center coordinates of grid box were set according to the original ligand. The MOL2 structure files of the active ingredients in Gancao─Gegen were downloaded from the TCMSP database based on the python script. In addition, Polar hydrogen was added, charge assigned, non‐polar hydrogen merged, and rotatable chemical bonds configured using Raccoon software, the PDBQT structure files of compounds were saved. Eventually, the high‐throughput screening of active ingredients and core targets were performed by AutoDock Vina software and python script. Also, HipHop algorithm was employed to construct pharmacophore models with common features for the core target, predicting the biological activity of candidate compounds.^[^ ^51^ ^]^ The steps were as follows: selection and preparation of the training set, generation of compound 3D structures, incorporation of compound activity parameters, definition of pharmacophore feature elements, construction of the common feature pharmacophore, and analysis of an external test set.

Molecular dynamics simulation was conducted by GROMACS 2018.8, and the simulation processes were carried out by the GROMOS96 43a1 force field.^[^ ^52^ ^]^ During the simulation, the box was solvated with SPC water models, and the box type was set to a dodecahedron,^[^ ^52^, ^53^ ^]^ the distance between the edge of the box and the protein edge was set to 1 nm, and the ions were added to maintain the electrical neutrality of the system. The results of molecular docking were taken as the initial structure, and the energy minimization was performed by using steepest descent minimization method.^[^ ^52^ ^]^ Then, the system was carried out heat bath (NVT, Number of particles, Volume, and Temperature) and pressure bath (NPT, Number of particles, Pressure, and Temperature).^[^ ^54^ ^]^ V‐rescale methods were chosen for temperature coupling, the reference temperature was set to 300 K, the step length was set to 2 fs, and the duration was set to 100 ps.^[^ ^55^ ^]^ Parrinello–Rahman methods were chosen for pressure coupling, and the duration was set to 100 ps.^[^ ^55^ ^]^ Finally, the molecular dynamics simulation duration was set to 20 ns and the simulation trajectories were saved. The short‐range electrostatics interaction was calculated by cut‐off method, and the cut‐off radius was set to 1.2 nm. The Particle Mesh Ewald (PME) method was used to calculate the long‐range electrostatics interaction.^[^ ^56^ ^]^

### Preparation of GLA‐PUE@gel

GLA remained stable after being heated at 120 °C for 2 h, frozen at −20 °C for 8 h, and then thawed at 4 °C for 4 h, indicating good thermal stability.^[^ ^30^, ^57^ ^]^ First, 38.5 mg of PUE was added to 1 mL of PBS (pH 7.4), and heated to 85 °C to completely dissolve PUE. Then, 50 µL of GLA (20 mg mL^−1^) ethanol solution was added and the mixture was heated for an additional 5 minutes. Afterward, the solution was allowed to cool naturally to 25 °C, resulting in the formation of a jelly‐like uniform stable hydrogel GLA‐PUE@gel.

### Establishing an HPLC Methodology for Content Determination

The amount of released GLA and PUE was measured by HPLC (LC‐20A, Shimadzu, Japan), and the separation was carried out on InertSustain C18 (250 mm × 4.6 mm, 5 µm). PUE chromatographic conditions: mobile phase was methanol:water (28:72, v/v), flow rate was 1.0 mL mi^−1^n, detection wavelength was 242 nm, column temperature was 30 °C, injection volume was 10 µL. GLA chromatographic conditions: mobile phase was methanol:1% acetic acid (75:25, v/v), flow rate was 1.0 mL mi^−1^n, detection wavelength was 280 nm, column temperature was 25 °C, injection volume was 10 µL. The above chromatographic conditions complied with the validation of HPLC methodology.

### In Vitro Hydrogels Degradation and Drug Release Studies

Degradability test of hydrogels was determined via a gravimetric method.^[^ ^58^ ^]^ 1 mL of hydrogels was prepared in a 2.5 mL centrifuge tube, whereafter, 1 mL of PBST solution (pH 7.4, 0.25% Tween 80) as degradation solution was added to hydrogel. The mixture was cultured at 37 °C (100 rpm). At the predetermined time interval, the degradation solution was taken away and the remaining hydrogels were weighted. All samples were tested in triplicates. The degradation percentage of the hydrogels was determined by the following Equation (6):

(6) $$\mathrm{Degradation}\;\mathrm{percentage}\;=\left[\left(M_{1}-M_{t}\right)/\left(M_{1}-M_{0}\right)\right]\times 100\%$$

where *M*_1_ represents the initial weight of the centrifuge tube and hydrogels, *M*_0_ refers to the weight of the empty centrifuge tube, and *M_t_* indicates the weight of the centrifuge tube and remaining hydrogels at time *t*.

Subsequently, the concentration of GLA was obtained in the range of 1–200 µg mL^−1^ according to the standard curve (7):

(7) $$A=24778.26C+5230.26\left(R^{2}=0.9999\right)$$

where *A* was peak area, and C was the concentration of GLA.

Also, the concentration of PUE was obtained in the range of 1–200 µg mL^−1^ according to the standard curve (8):

(8) $$A=43790.74C-15411.11\left(R^{2}=0.9999\right)$$

where *A* was peak area, and C was the concentration of PUE.

The release profiles of GLA and PUE were fitted by the zero order, first order, Higuchi model, Wei‐bull model and Ritger‐Peppas model, respectively.^[^ ^59^ ^]^

### Cytotoxicity Measurement

Cell viability was assessed by the CCK‐8 assay. RAW264.7 cells were plated into 96‐well plates at a density of 5000 cells/well and cultured overnight. The spent culture medium was carefully removed and replaced with fresh medium supplemented with varying concentrations of GLA, PUE, GLA/PUE, and GLA‐PUE@gel solution. Additionally, a comparative analysis of the impact of H_2_O_2_ exposure for 2 and 4 h on A549 cell viability was conducted, to determine the optimal duration for establishing the oxidative stress model. Finally, the absorbance at 450 nm was measured by microplate reader (Molecular Devices ID5, USA), and the percentage of alive cells was calculated using Graphpad Prism 8.0.2 software.

### Scanning Electron Microscopy

PUE@gel and GLA‐PUE@gel were fractured using liquid nitrogen to expose the interface, and directly affixed to a conductive adhesive. Subsequently, sputter coating with gold was performed using a Quorum SC7620 sputter coater at a current of 10 mA. The morphology of PUE@gel and GLA‐PUE@gel was then captured using scanning electron microscope (Hitachi Regulus 8100, Japan), with an acceleration voltage of 5 kV and an SE2 secondary electron detector.

### Differential Scanning Calorimeter

Using a differential scanning calorimeter (DSC 500, Germany), the temperature‐induced phase transition processes of GLA, PUE, GLA/PUE, and GLA‐PUE@gel were investigated to characterize *Tg*. Alumina was used as the blank reference, and a nitrogen purge rate of 40 mL mi^−1^n was applied. The temperature program was set to ramp from 25 to 500 °C at a rate of 10 °C/min.

### X‐Ray Diffraction

GlA, PUE, GLA/PUE, PUE@gel, GLA‐PUE@gel, and PUE@gel+GLA were taken ≈50 mg of each sample for analysis. The sample was measured using an X‐ray diffractometer (SmartLab SE, RIGAKU, Japan) at room temperature, scanning in a 2θ rotation range of 3–40° at a speed of 10°/min, with a step size of 0.02°. Data analysis was performed using Origin 2022.

### Rotational Rheometry Measurement

Using the rotational rheometer (HR10 CED‐D‐LBY‐001, TA, USA), the rheological properties of PUE@gel and GLA‐PUE@gel were studied with the small amplitude oscillatory time sweep module. The parameters were as follows: the measuring clamp was a 40 mm parallel plate, temperature was 25 °C, soak time was 0 s, duration was 300 s, strain was 1%, frequency was 1 rad s^−1^, and the measured indices were the elastic storage modulus (G′) and the loss modulus (G″).

### Confocal Laser Scanning Microscope

Confocal laser scanning microscope (CLSM, Carl Zeiss LSM 700, Germany) was utilized to detect intracellular ROS levels in A549 cells, with groups including: Control, H_2_O_2_, and GLA‐PUE@gel. Cells were plated at a density of 10^6^ cells/well in confocal dishes and incubated for 24 h. H_2_O_2_ was added to induce oxidative stress, followed by treatment with GLA‐PUE@gel to investigate its antioxidant activity. Sample processing was involved in discarding the spent culture medium and washing once with serum‐free culture medium. Cells were then treated with a 10 µM fluorescent probe H_2_DCFDA solution and incubated at 37 °C in the dark for 30 minutes, and the free H_2_DCFDA was removed by serum‐free culture medium. Ultimately, CLSM was employed to quantify the fluorescence intensities of the samples and conducted data analysis with ZEN microscopy software.

### Griess Reagent Nitrite Measurement

Griess assay was used to detect the change of NO content in the cell supernatant after administration. First, Griess B solution was prepared: 1 g of sulfonamide was weighed, 5.88 mL of phosphoric acid solution was added, and ultrapure water was supplemented. The volume was fixed in a 100 mL volumetric flask and kept away from light. Preparation of Griess B solution: 0.1 g of *α*‐naphthylamine hydrochloride was weighed and added with ultrapure water to a 100 mL volumetric flask for constant volume and stored in dark. The standard curve of NaNO_2_ was drawn: 17.0 mg NaNO_2_ was accurately weighed, 1.23 mL of distilled water was added, and it was dissolved by vortex to obtain 200 mM mother liquor. NaNO_2_ solutions with concentrations of 100, 50, 25, 12.5, 6.25, and 0 µM (100 µL/well, n = 3) were added to 96‐well plates, and then the mixed solution of Griess A and Griess B (1:1) was added in the dark for 10 min. OD value was measured at 540 nm of a microplate reader. The standard curve of NaNO_2_ was drawn with the concentration as horizontal axis and OD values as the vertical axis.

RAW264.7 cells were prepared into a cell suspension with a concentration of 5 × 10^4^ cells/mL, inoculated in 96‐well plates (100 µL/well) and cultured in a CO_2_ incubator at 37 °C with 5% CO_2_. Groups were set up, including blank, LPS, and experimental group. After 24 h, the experimental group was treated with varying concentrations of GLA, while the LPS group and the blank group were supplemented with an equal volume of DMEM culture medium. After another 6 h of incubation, the experimental and LPS groups were received 10 µg mL^−1^ LPS solution, and the blank group received an equal volume of DMEM culture medium. Following an 18‐h incubation, the 96‐well plate was removed from the incubator and shaken for 10 minutes to ensure uniform mixing of NO produced in the supernatant. The culture supernatant from the 96‐well plate (100 µL/well) was then transferred to a new 96‐well plate, and Griess reagent (Griess A: Griess B = 1:1, 100 µL/well) was added. The plate was incubated in the dark for 10 minutes, after which OD value was measured at 540 nm of a microplate reader. The content of NO in the supernatant of each group was calculated using the standard curve of NaNO_2_.

### DPPH Assay

DPPH, also known as 1,1‐diphenyl‐2‐picrylhydrazyl, was a very stable nitrogen‐centered free radical. Its anhydrous ethanol solution appears purple and has a maximum absorption at a wavelength of 517 nm, where the absorbance was linearly related to the concentration. When a free radical scavenger was added to it, DPPH· can be quenched or replaced, leading to a reduction in the number of free radicals, a decrease in absorbance, and a lightening of the solution's color. Thus, this change can be used to evaluate the ability to scavenge free radicals. In this experiment, the DPPH method was used to detect the free radical scavenging ability of GLA. DPPH reagent was accurately weighed 6 mg, dissolved in 70% ethanol and diluted to a 50 mL volumetric flask to obtain a concentration of 0.3 mM DPPH solution, which was placed in a refrigerator in the dark and refrigerated for later use. Subsequently, GLA solutions with concentrations of 12.5, 6.25, 3.125, and 1.56 µM were prepared using 70% ethanol, the reaction was kept away from light for 30 min at 25 °C, and the absorbance value was measured at 517 nm. The parallel operation was performed three times, and the mean value was taken.

### Western Blot

The protein expression of IL‐1*β* and TNF‐*α* was assessed using Western blot. RAW264.7 cells treated were harvested to extract total protein, which was quantified by the Micro BCA Protein Assay Kit. The proteins were combined with 5×loading buffer and boiled at 100 °C for 10 minutes to fully denature them, and the prepared samples underwent Polyacrylamide Gel Electrophoresis at a constant voltage for 60 minutes. Subsequently, the separated proteins were transferred onto PVDF membranes, which were then blocked at 25 °C using a 5% bovine serum albumin solution for 2 h. Then, the PVDF membranes were incubated overnight at 4 °C with primary antibodies, washed with TBST, and incubated at 25 °C with secondary antibodies for 2 h. After another round of TBST washes, the protein expression was visualized using the ChemiDoc MP Imaging System (BioRad, USA), and the grayscale values of the bands were quantitatively analyzed using Image‐J.

### Flow Cytometry

Flow cytometry was employed to detect the expression of IL‐1*β* in RAW264.7 cells, with the following groups set up: Control, LPS, and GLA‐PUE@gel. Cells were plated at a density of 10^6^ cells/well in 6‐well plates and incubated for 24 h. LPS was added to induce an inflammatory model, followed by the addition of GLA‐PUE@gel for 24 h to explore its anti‐inflammatory activity. Sample processing involved removal of spent culture medium, washing once with pre‐cooled PBS (centrifuged at 3200 rpm for 5 minutes). Cells were fixed with 1 mL of 4% paraformaldehyde at 25 °C for 20 min, followed by centrifugation and removal of the supernatant. Membrane permeabilization was carried out with 3 mL/L Triton X‐100 incubation for 10 min (except Control group), followed by washing once with pre‐cooled PBS. IL‐1 beta Polyclonal antibody (5 µL/100 uL) was added and incubated at 25 °C for 20 min, followed by addition of CoraLite647‐conjugated Mouse Anti‐Heavy Chain of Rabbit IgG (5 µL/100 uL) at 25 °C for 15 min. Subsequently, cells were washed with 1 mL PBS and kept in the dark throughout. Finally, 1 mL PBS was added for detection using flow cytometer (BD Accuri C6, USA), and the results were processed using Flowjo 10.10 software.

Furthermore, flow cytometry was employed to detect the surface markers CD86 and CD206 on THP‐1 cells, with groups including: Control, LPS, IL‐4, and GLA‐PUE@gel. Cells were plated at a density of 10^6^ cells/well in 6‐well plates and incubated for 24 h. PMA was used to induce the transition of THP‐1 cells from a suspended state to an adherent state for 4 h, followed by the addition of LPS to induce an inflammatory M1 model and IL‐4 to induce an anti‐inflammatory M2 model. GLA‐PUE@gel was added for 24 h to explore its anti‐inflammatory molecular mechanism. Sample processing involved removal of spent culture medium, washing once with pre‐cooled PBS. PE Anti‐Mouse CD86 Antibody (5 µL/100 uL) was added and incubated at 25 °C for 30 min in the dark. Cells were then fixed with 1 mL of 4% paraformaldehyde for 20 min, followed by centrifugation and removal of the supernatant. Membrane permeabilization was carried out with Triton X‐100 (3 mL/L) incubation for 10 min (except Control and LPS groups), followed by washing once with pre‐cooled PBS. APC Anti‐Mouse CD206/MMR Antibody (5 µL/100 uL) was added and incubated at 25 °C for 30 min, followed by washing with 1 mL PBS and keeping the samples in the dark throughout. Finally, 1 mL PBS was added for detection using flow cytometer, and the results were processed using Flowjo 10.10 software.

Also, flow cytometry was utilized to detect intracellular ROS levels in A549 cells, with groups including: Control, H_2_O_2_, and GLA‐PUE@gel. Cells were plated at a density of 10^6^ cells/well in 6‐well plates and incubated for 24 h. H_2_O_2_ was added to induce oxidative stress, followed by treatment with GLA‐PUE@gel to investigate its antioxidant activity. Sample processing was involved in discarding the spent culture medium and washing once with serum‐free culture medium. Cells were then treated with a 10 µM fluorescent probe H_2_DCFDA solution and incubated at 37 °C in the dark for 30 minutes, and the free H_2_DCFDA was removed by serum‐free culture medium. In addition, there were also assessments of indicators related to endoplasmic reticulum stress: CHOP, CRT, and HMGB1. Finally, 1 mL PBS was added for detection using flow cytometer, and the results were processed using Flowjo 10.10 software.

### Drug‐Like Properties and Toxicity Prediction

SwissADME platform (http://www.swissadme.ch/) was utilized to predict the drug‐like properties of GLA and PUE, encompassing a comprehensive analysis of molecular weight, MlogP values, hydrogen bond donor and acceptor counts, rotatable bond counts, and atom counts. The compounds' suitability as drug candidates was assessed against a battery of well‐established pharmacokinetic and pharmacodynamic benchmarks, such as Lipinski's Rule of Five for general drug‐likeness, Ghose's criteria for distinguishing between lead‐like and drug‐like properties, Veber's guidelines for predicting oral bioavailability, Egan's metrics for molecular complexity, and Muegge's principles for optimizing lead compounds. This multifaceted evaluation ensures a thorough assessment of the drug‐like potential of GLA and PUE. Additionally, toxicity analysis, as an essential aspect of understanding compound toxicity, was part of the pharmacokinetic properties of drugs. ProTox 3.0 Web‐server (https://tox.charite.de/protox3/)^[^ ^60^ ^]^ was utilized to test the toxicity categories of GLA and PUE, and calculate the LD_50_ values.

### In Vivo Safety Evaluation of Drugs

C57BL/6J mice were randomly divided into four groups (n = 6): Control, GLA/PUE, GGD, and GLA‐PUE@gel. All treatment groups (50 mg kg^−1^ GLA/PUE, 50 mg kg^−1^ GLA‐PUE@gel, and a clinically equivalent dose of GGD) were administered via intragastric gavage. After 12 h, mice were euthanized, and the heart, liver, spleen, lungs, and kidneys were collected. The tissues were fixed in a universal fixative, followed by paraffin embedding, sectioning, and H&E staining.

### Data Statistics

Unpaired t‐tests and analysis of variance (ANOVA) tests with multiple comparisons were conducted, as appropriate, to establish statistical significance between groups (where *p* < 0.05 was deemed significantly different) using Graphpad Prism 8.0.2 software. All data were representative of two to three independent experiments and were presented as means ± SD, unless otherwise specified. The significance levels were denoted as follows: *p < 0.05, **p < 0.01, ***p < 0.001, and ****p < 0.0001, while “ns” indicates non‐significance.

## Conflict of Interest

The authors declare no conflict of interest.

## Author Contributions

J.Q., Y.W., and H.C. contributed equally to this work. W.Z. conceived and directed the project. J.Q., Y.W., H.C., K.W., P. Z., Y.D., and B.X. conducted experiments and data analysis. J.Q. and W.Z. wrote the manuscript.

## Supporting information



Supporting Information

## Data Availability

The data that support the findings of this study are available in the supplementary material of this article.
