# Supplementary material for: Advancing the Identification of Bioactive Molecules and the Construction of a Synergistic Drug Delivery System in Combating Lung Injury
Source: Adv Sci (Weinh). 2025 Mar 17;12(18):2407802. doi: 10.1002/advs.202407802 (PMC12079430; doi:10.1002/advs.202407802)
Supplement: Supplementary file 1 — Supporting Information [file ADVS-12-2407802-s001.docx]

***Supplementary Material***

**Advancing the Identification of Bioactive Molecules and the Construction of a Synergistic Drug Delivery System in Combating Lung Injury**

*Jianhong Qi†, Yanxia Wang†, Huan Chen†, Kaitian Wu, Pei Zhou, Yue Dou, Bingqi Xiong, Wei Zhou**

Department of Pharmaceutics, China Pharmaceutical University, Nanjing 210009, China.

**† These authors have contributed equally to this work.**

*** Corresponding authors**:

Wei Zhou, professor

Department of Pharmaceutics, China Pharmaceutical University

#24 Tong Jia Xiang, Gulou District, Nanjing 210009, China

E-mail: zw_why@cpu.edu.cn

**
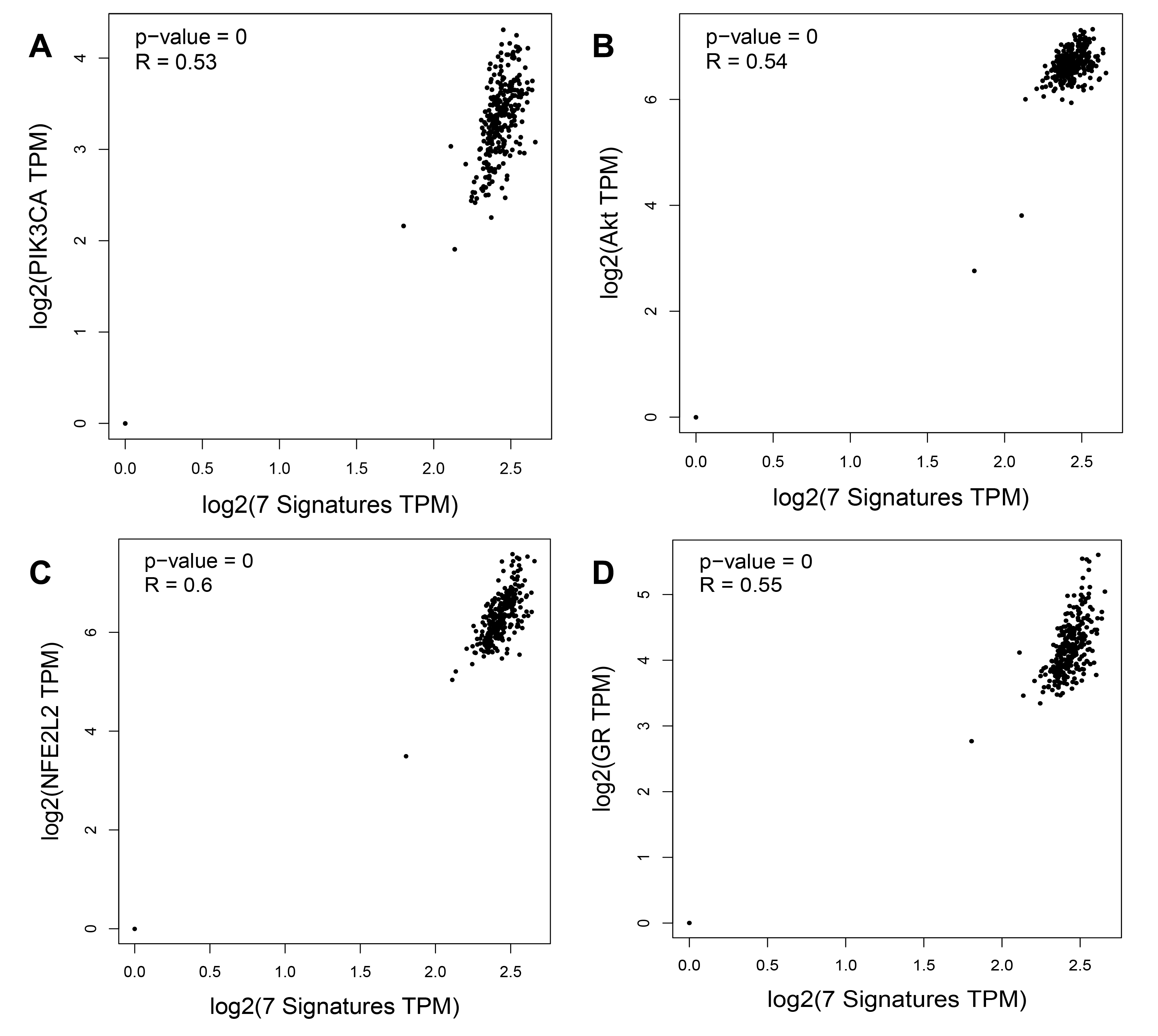
**

**Figure S1. Genetic correlations with 7 core targets.** (A) PI3K. (B) Akt. (C) NFE2L2. (D) GR.

**
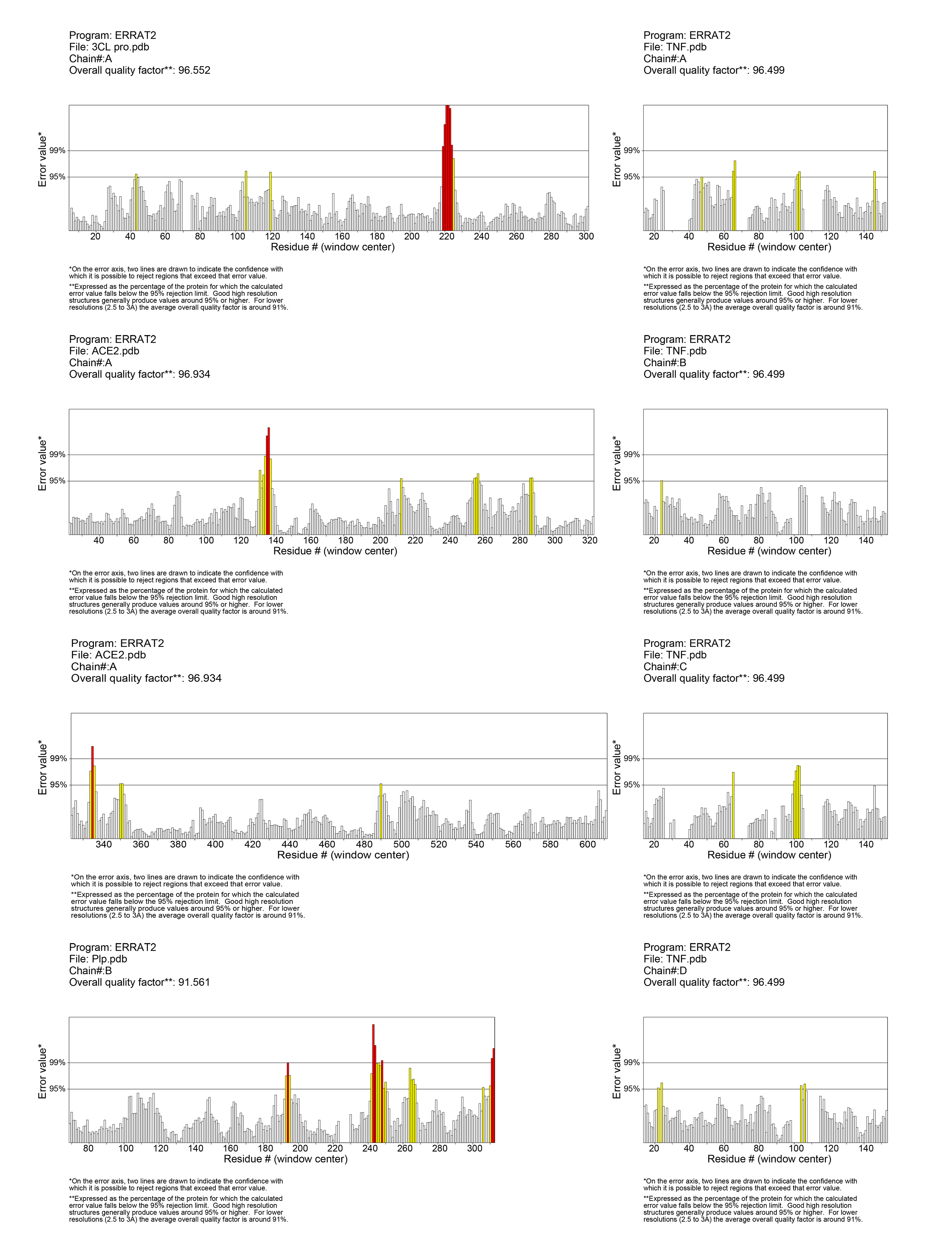
**

**Figure S2. Calculation of the precise values of ACE2, 3CLpro, Plp, and TNF protein crystal structures.**

**
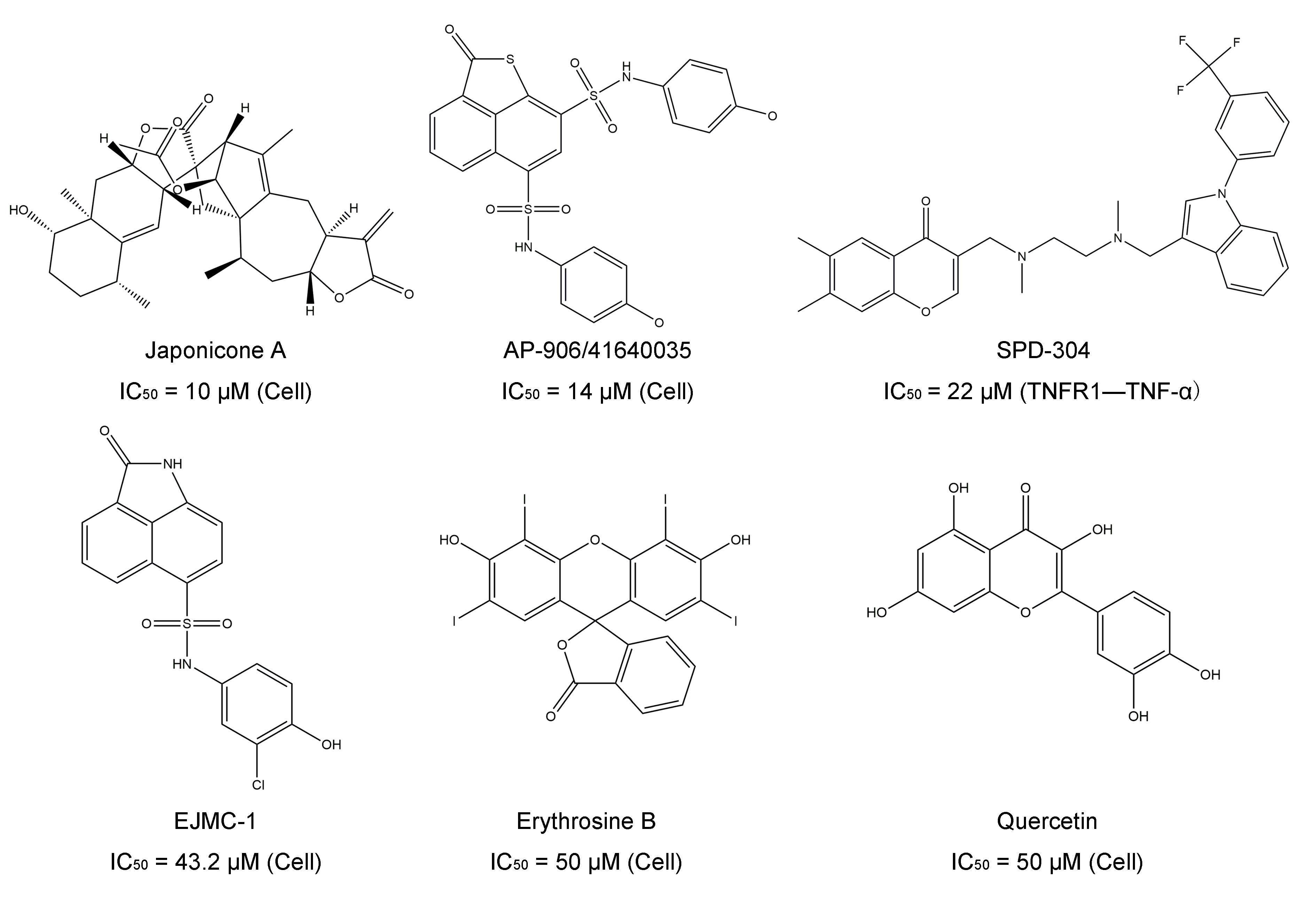
**

**Figure S3. Structures and IC50 of TNF-α inhibitors.**

**
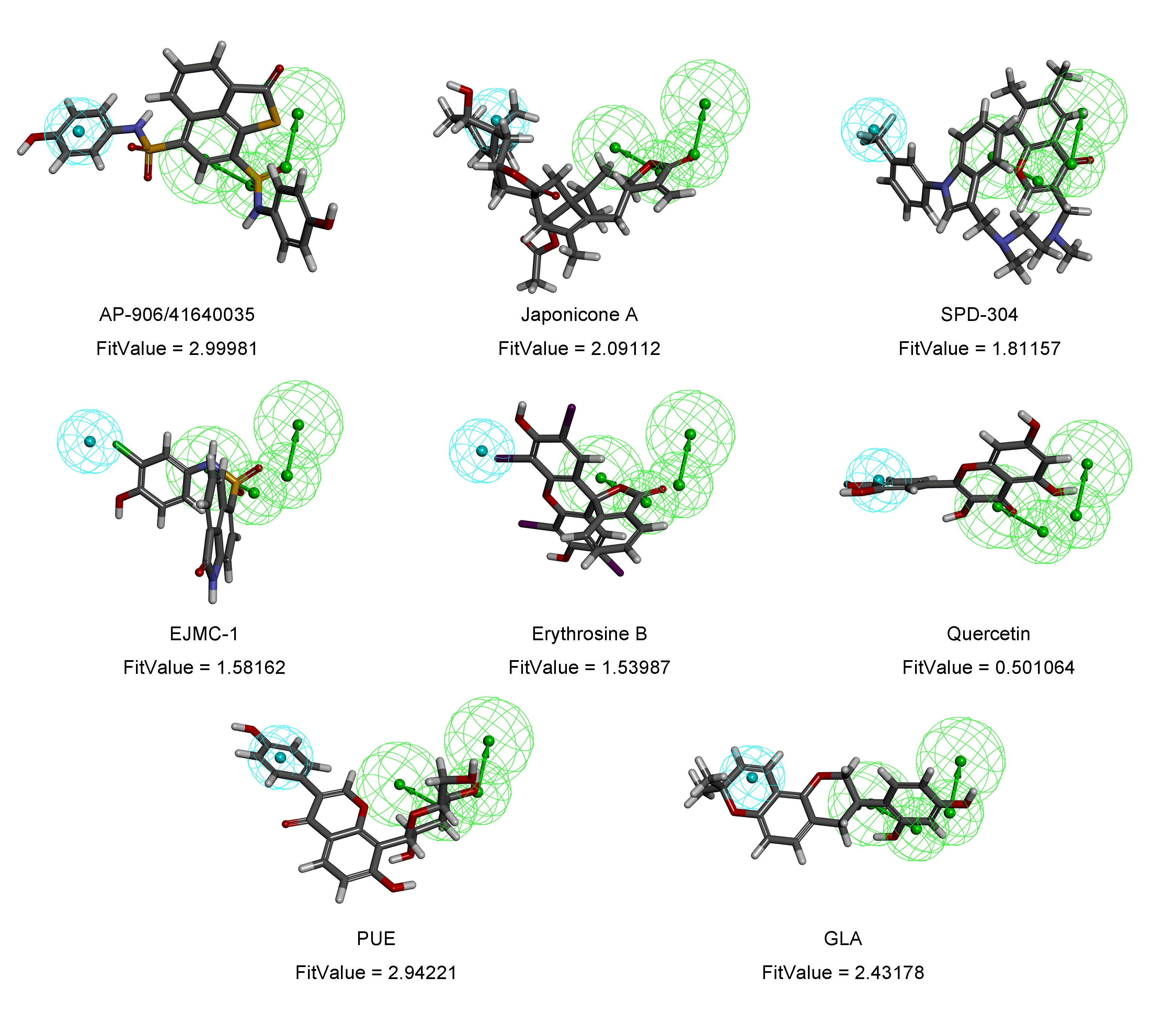
**

**Figure S4. The matching diagram of pharmacophore model 05 with active compounds.**


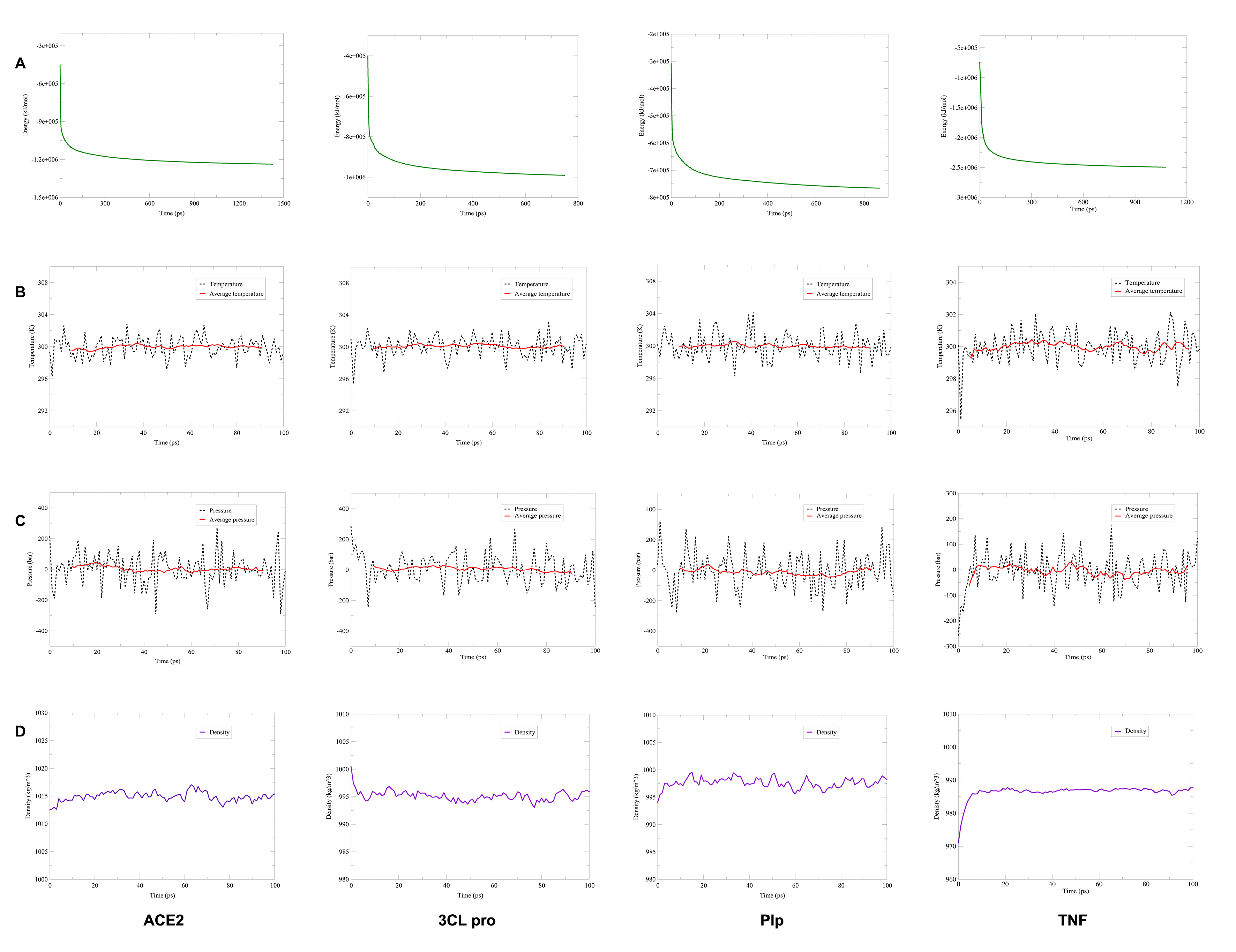


**Figure S5. Pre-processing dynamics simulation of GLA with the key targets against COVID-19.** (A) System energy minimization processing. (B) System temperature stabilization processing. (C) System pressure stabilization processing. (D) System density stabilization processing.


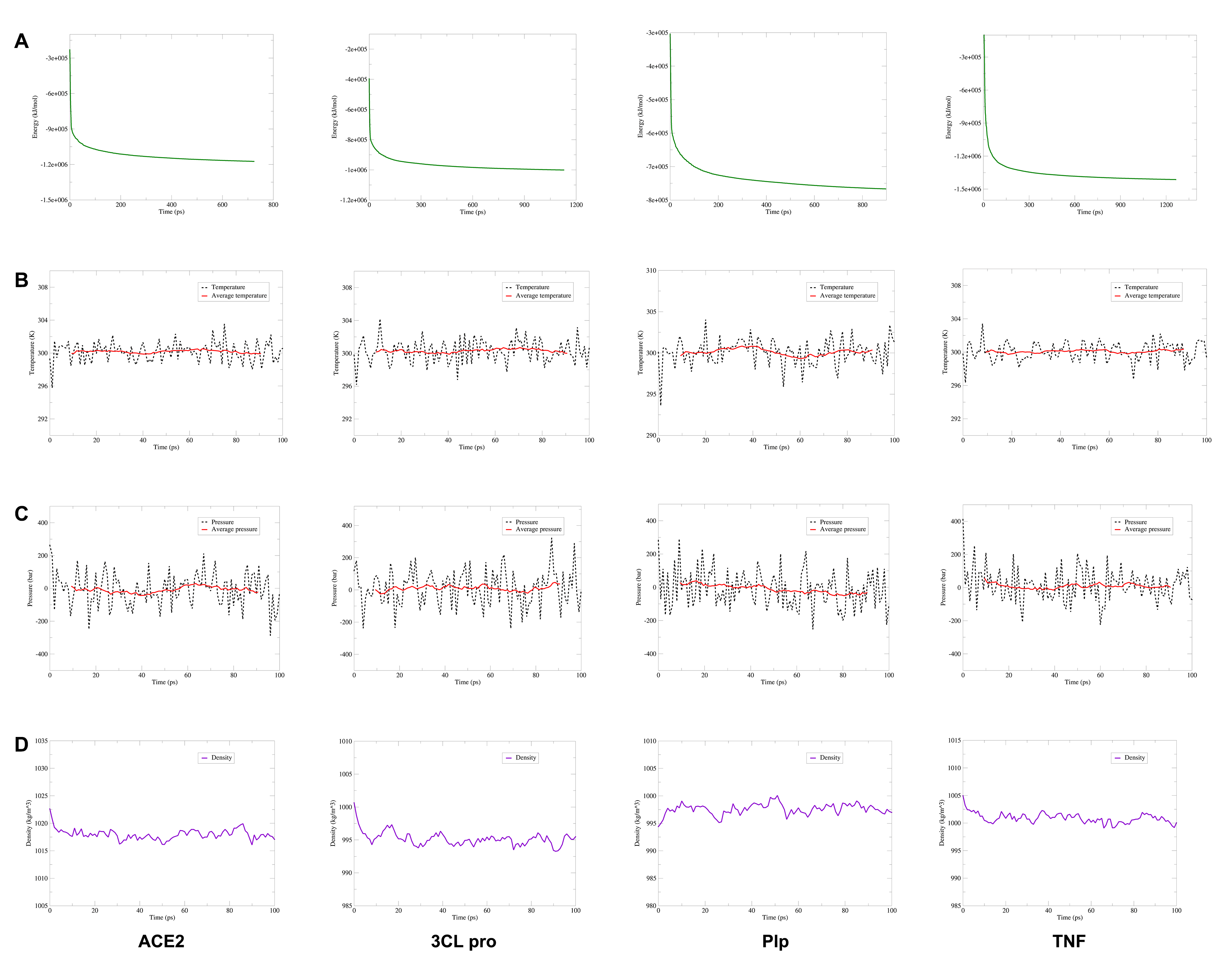


**Figure S6. Pre-processing dynamics simulation of PUE with the key targets against COVID-19.** (A) System energy minimization processing. (B) System temperature stabilization processing. (C) System pressure stabilization processing. (D) System density stabilization processing.


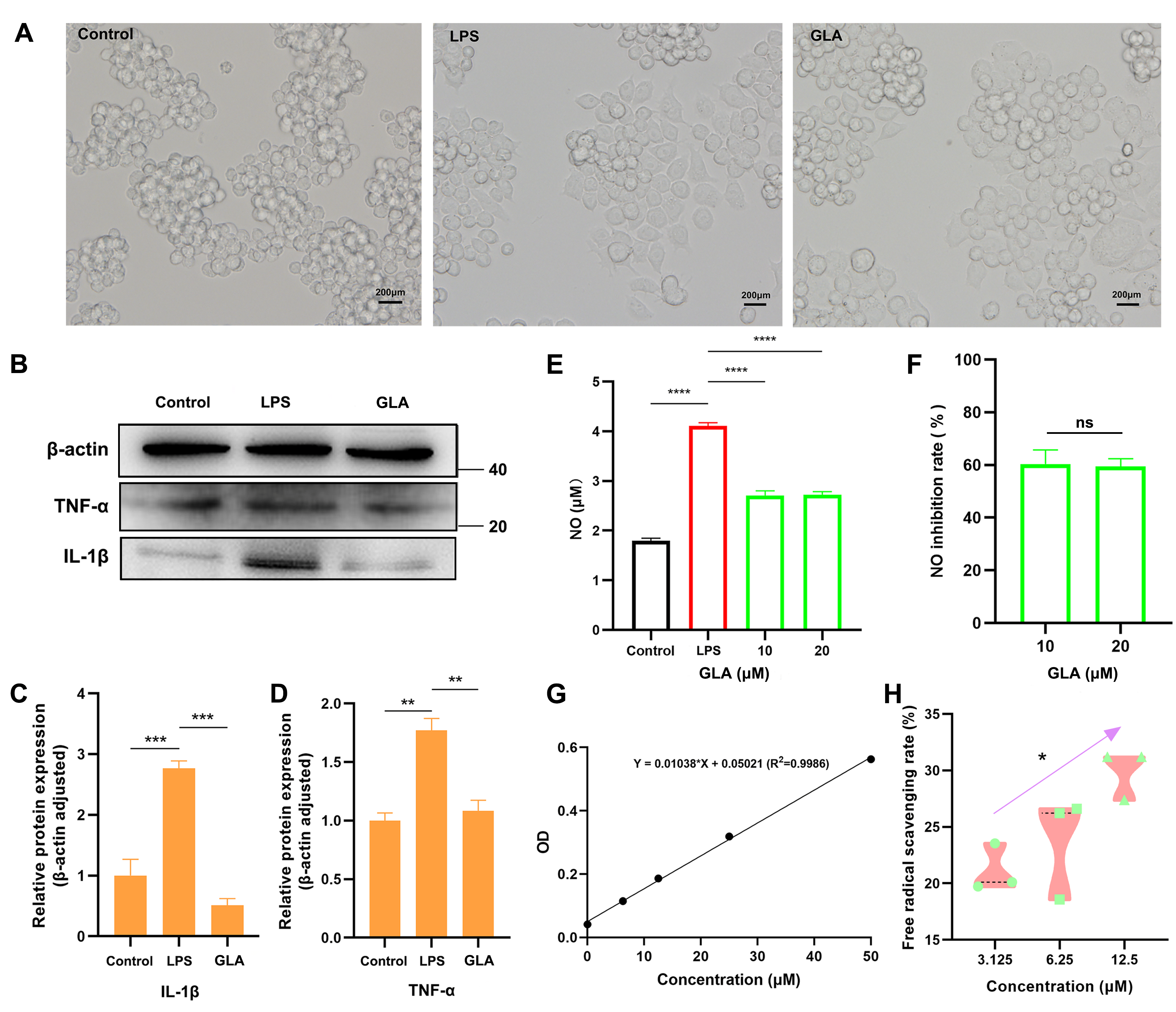


**Figure S7. Anti-inflammatory and antioxidant effects of GLA.** (A) Cell morphology in different groups, scale bar: 200 μm. (B) Protein bands of TNF-α and IL-1β. (C) Regulatory effect of GLA on the expression of IL-1β in macrophages. (D) Regulatory effect of GLA on the expression of TNF-α in macrophages. (E) The impact of GLA on the secretion of NO content in M1 macrophages. (F) The impact of GLA on NO inhibition rate in M1 macrophages. (G) The standard curve of NaNO_2_. (H) Free radical scavenging capacity of GLA at different concentrations. **p* < 0.05, ***p* < 0.01, ****p* < 0.001, and *****p* < 0.0001.


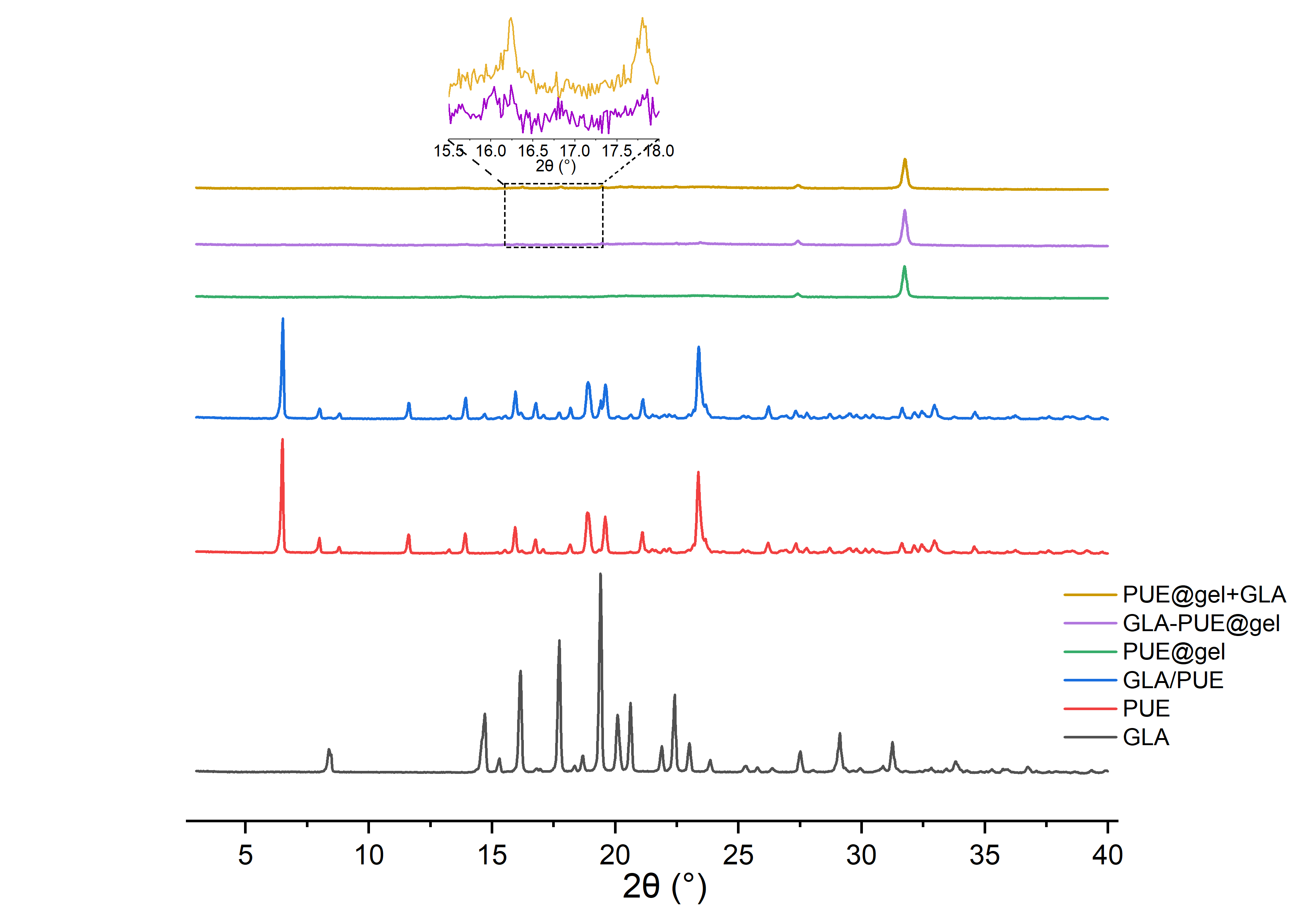


**Figure S8. XRD of free GLA, PUE, GLA/PUE, PUE@gel, GLA-PUE@gel, and PUE@gel+GLA.**

**
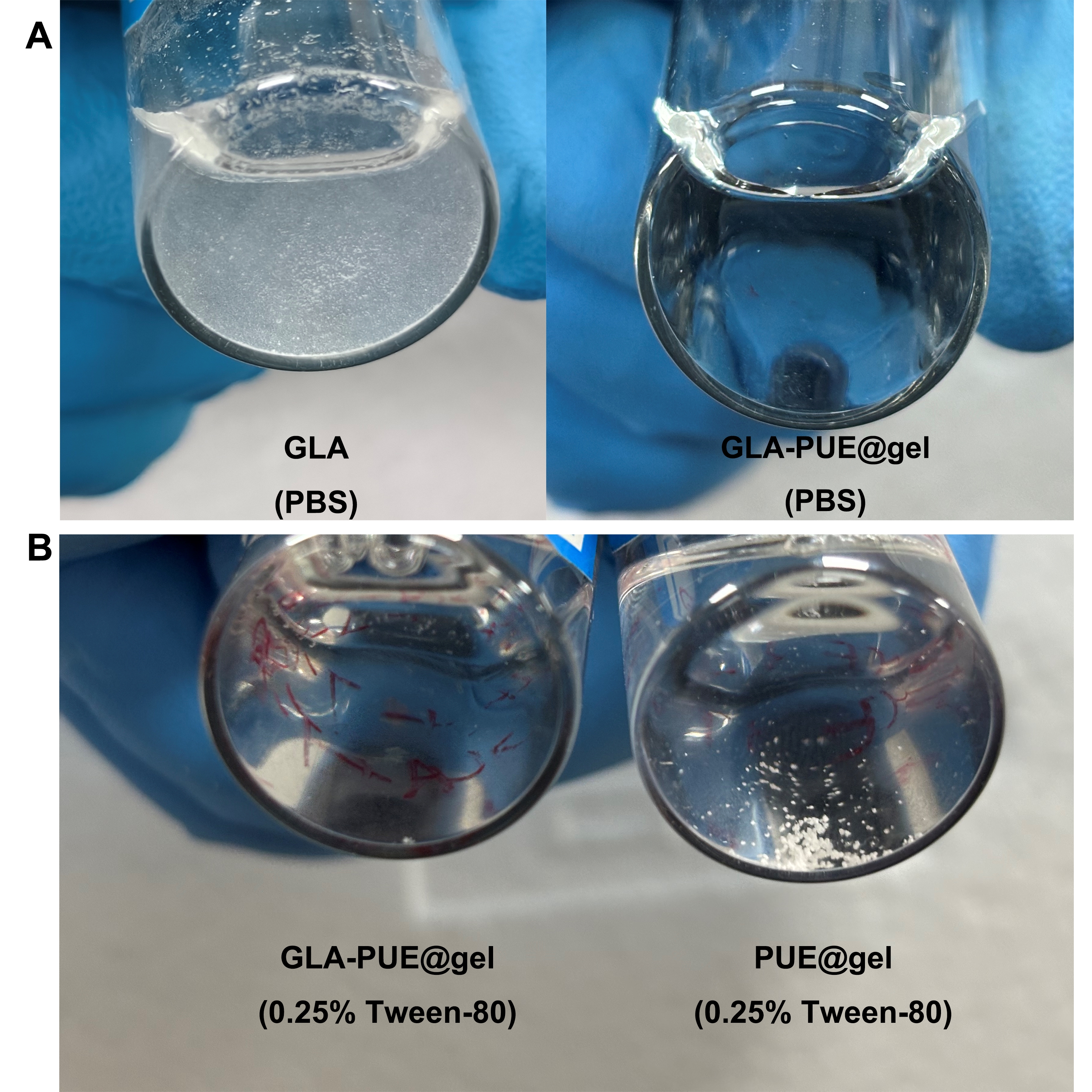
**

**Figure S9.** **The solubility of GLA, GLA-PUE@gel, and PUE@gel under different conditions.**


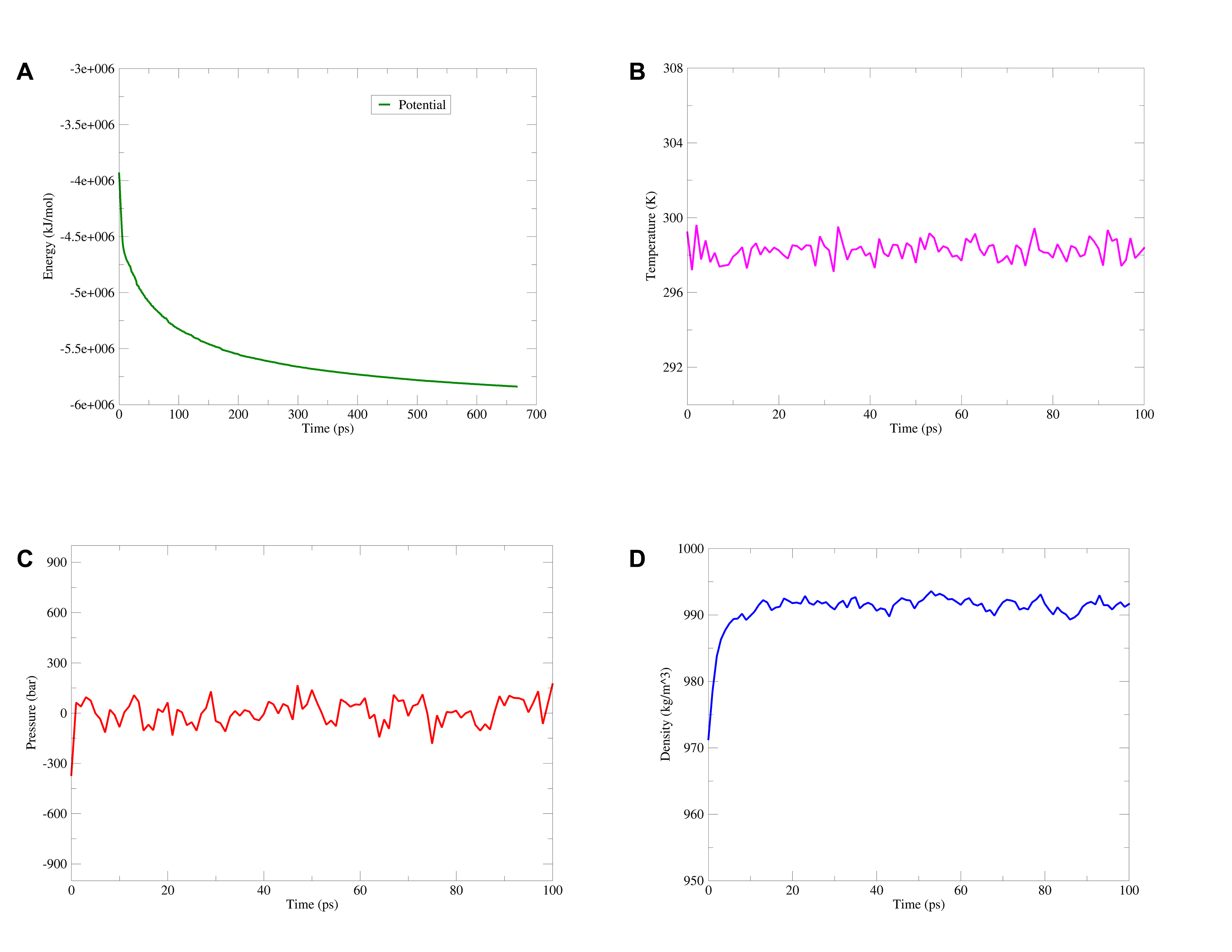


**Figure S10. Pre-processing self-assembly simulation of PUE.** (A) System energy minimization processing. (B) System temperature stabilization processing. (C) System pressure stabilization processing. (D) System density stabilization processing.


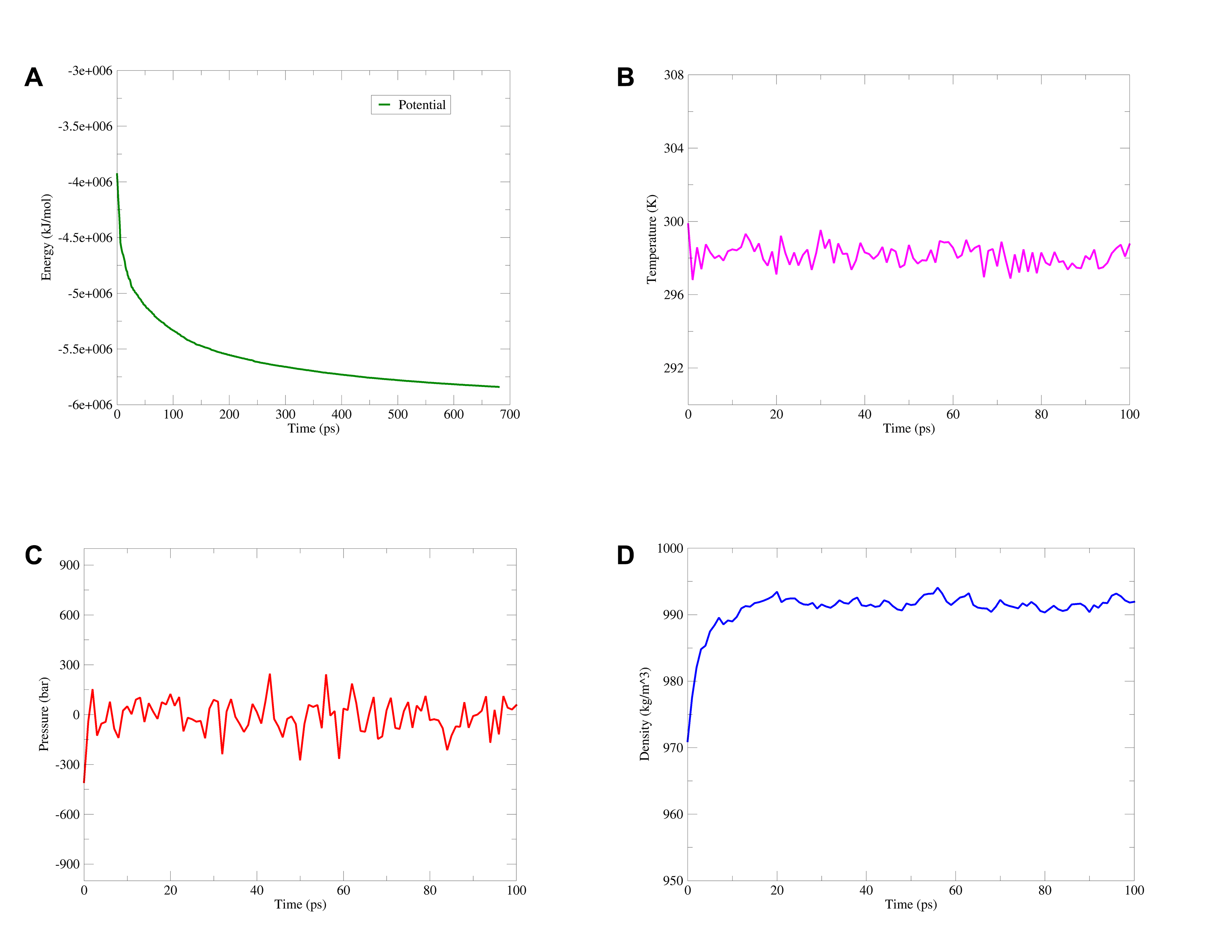


**Figure S11. Pre-processing self-assembly simulation of GLA-PUE@gel.** (A) System energy minimization processing. (B) System temperature stabilization processing. (C) System pressure stabilization processing. (D) System density stabilization processing.


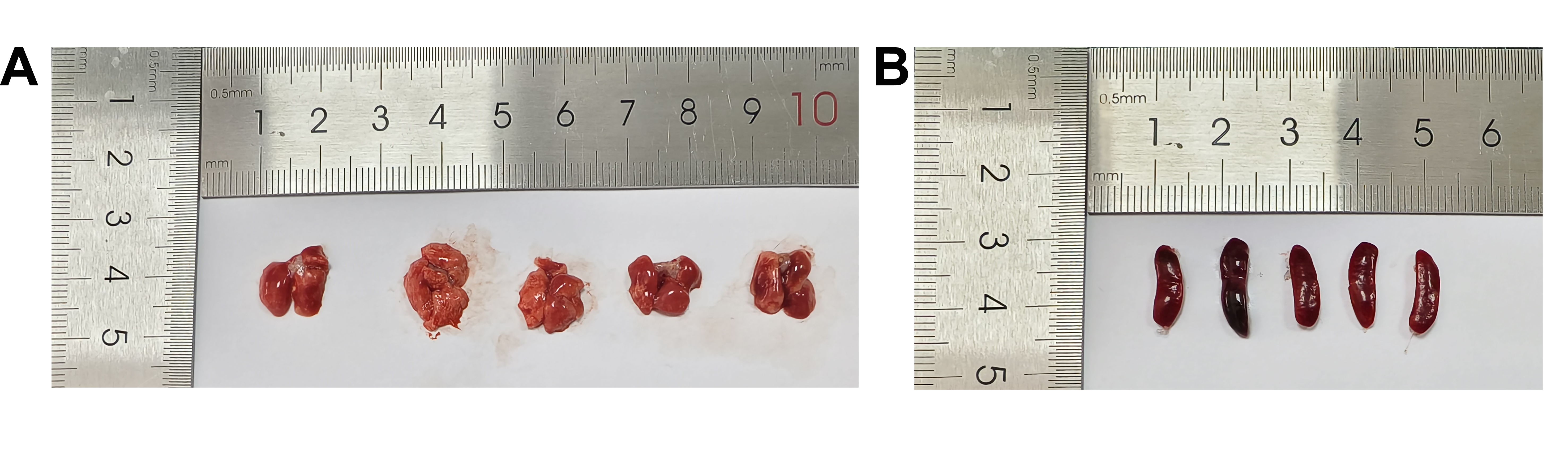


**Figure S12.** **Macroscopic improvement of lung and spleen morphology in ALI mice treated with GLA-PUE@gel.** (A) Lung; (B) Spleen. From left to right: Control, LPS, GLA/PUE, GLA-PUE@gel, and GGD.

**Table S1.** The relationship parameters of countries and regions that published literature on the TCM treatment of COVID-19.

| Count | Centrality | Year | Keywords |
| --- | --- | --- | --- |
| 142 | 0.93 | 2020 | PEOPLES R CHINA |
| 12 | 0.21 | 2020 | INDIA |
| 11 | 0.31 | 2020 | USA |
| 10 | 0.15 | 2022 | TAIWAN |
| 5 | 0.37 | 2021 | SAUDI ARABIA |
| 4 | 0.12 | 2020 | ENGLAND |
| 4 | 0.17 | 2020 | GERMANY |
| 4 | 0.22 | 2020 | ITALY |
| 3 | 0 | 2023 | AUSTRALIA |
| 2 | 0 | 2021 | TURKEY |
| 2 | 0 | 2021 | SOUTH KOREA |
| 2 | 0 | 2021 | POLAND |
| 2 | 0 | 2021 | IRAN |
| 2 | 0.18 | 2021 | BANGLADESH |
| 2 | 0 | 2022 | PORTUGAL |
| 2 | 0 | 2022 | BRAZIL |
| 1 | 0.01 | 2020 | SPAIN |
| 1 | 0 | 2021 | CZECH REPUBLIC |
| 1 | 0 | 2021 | INDONESIA |
| 1 | 0 | 2021 | OMAN |
| 1 | 0 | 2022 | ARGENTINA |
| 1 | 0 | 2022 | SERBIA |
| 1 | 0 | 2022 | UKRAINE |
| 1 | 0 | 2022 | EGYPT |
| 1 | 0 | 2022 | SWITZERLAND |
| 1 | 0 | 2022 | SWEDEN |
| 1 | 0 | 2022 | TUNISIA |
| 1 | 0 | 2022 | THAILAND |
| 1 | 0 | 2022 | IRAQ |
| 1 | 0 | 2023 | PHILIPPINES |
| 1 | 0 | 2023 | PAKISTAN |

**Table S2.** The relationship parameters of research institutions that published literature on the TCM treatment of COVID-19.

| Count | Centrality | Year | Keywords |
| --- | --- | --- | --- |
| 11 | 0.1 | 2020 | China Acad Chinese Med Sci |
| 11 | 0.08 | 2021 | Shanghai Univ Tradit Chinese Med |
| 10 | 0 | 2022 | Tianjin Univ Tradit Chinese Med |
| 8 | 0.12 | 2020 | Chinese Acad Sci |
| 7 | 0.03 | 2020 | Guangzhou Med Univ |
| 6 | 0.02 | 2022 | Shanghai Jiao Tong Univ |
| 6 | 0.03 | 2021 | Chinese Acad Med Sci & Peking Union Med Coll |
| 6 | 0.02 | 2020 | Beijing Univ Chinese Med |
| 5 | 0.01 | 2020 | Hubei Prov Hosp Tradit Chinese Med |
| 5 | 0 | 2022 | China Med Univ |
| 4 | 0.03 | 2022 | Nanjing Univ Chinese Med |
| 4 | 0 | 2022 | Asia Univ |
| 4 | 0 | 2020 | Huazhong Univ Sci & Technol |
| 4 | 0.02 | 2020 | Fudan Univ |
| 3 | 0 | 2022 | Anhui Med Univ |
| 3 | 0.02 | 2021 | Chinese Acad Med Sci |
| 3 | 0 | 2020 | Hong Kong Polytech Univ |
| 3 | 0 | 2022 | King Saud Univ |
| 3 | 0 | 2023 | Southern Med Univ |
| 2 | 0.01 | 2023 | Changchun Univ Chinese Med |
| 2 | 0 | 2023 | Univ Melbourne |
| 2 | 0 | 2022 | Shenyang Pharmaceut Univ |
| 2 | 0 | 2022 | Taipei Med Univ |
| 2 | 0 | 2023 | Natl Chung Hsing Univ |
| 2 | 0.01 | 2022 | Dalian Med Univ |
| 2 | 0 | 2022 | Shenzhen Univ |
| 2 | 0 | 2023 | China Med Univ Hosp |
| 2 | 0 | 2022 | State Key Lab Component Based Chinese Med |
| 2 | 0 | 2022 | Chang Gung Univ |
| 2 | 0 | 2022 | Capital Med Univ |
| 2 | 0 | 2023 | Haihe Lab Modern Chinese Med |
| 2 | 0 | 2022 | Beijing Inst Chinese Med |
| 2 | 0 | 2021 | Gazi Univ |
| 2 | 0.02 | 2022 | Zhejiang Chinese Med Univ |
| 2 | 0 | 2020 | Guangzhou Customs |
| 2 | 0 | 2022 | Taipei Med Univ Hosp |
| 2 | 0 | 2020 | Macau Univ Sci & Technol |
| 2 | 0 | 2022 | Chia Nan Univ Pharm & Sci |
| 2 | 0 | 2022 | Beijing Key Lab Basic Res Tradit Chinese Med Infec |
| 2 | 0 | 2021 | Chengdu Univ Tradit Chinese Med |
| 2 | 0.01 | 2023 | Cent South Univ |
| 1 | 0 | 2021 | Chongqing Med Univ |
| 1 | 0.01 | 2024 | First Affiliated Hosp Guangzhou Med Univ |
| 1 | 0.01 | 2024 | Dongguan Peoples Hosp |
| 1 | 0 | 2020 | Anhui Univ Chinese Med |
| 1 | 0 | 2021 | China Pharmaceut Univ |
| 1 | 0 | 2023 | Buddhist Tzu Chi Fdn Hosp |
| 1 | 0 | 2021 | First Hosp Qiqiha |
| 1 | 0.04 | 2021 | China Reg Res Ctr |
| 1 | 0 | 2021 | China Stand Med Informat Res Ctr |
| 1 | 0 | 2023 | Chongqing Tradit Chinese Med Hosp |
| 1 | 0 | 2021 | Guangdong Prov Key Lab Computat Sci & Mat Design |
| 1 | 0 | 2024 | Sanya Cent Hosp |
| 1 | 0 | 2021 | Du Jiang Yan Med Ctr |
| 1 | 0 | 2023 | Fifth Peoples Hosp Suzhou |
| 1 | 0 | 2023 | 924 Hosp Joint Logist Support Force PLA |
| 1 | 0 | 2021 | Cent Electrochem Res Inst |
| 1 | 0 | 2024 | Pingxiang Peoples Hosp |
| 1 | 0 | 2020 | Gansu Prov Hosp |
| 1 | 0 | 2022 | Al Azhar Univ |
| 1 | 0 | 2023 | Buddhist Compass Relief Tzu Chi Fdn |
| 1 | 0 | 2023 | Affiliated Hosp Nanjing Univ Chinese Med |
| 1 | 0 | 2024 | Sun Yat Sen Univ |
| 1 | 0.01 | 2022 | Anhui Univ Tradit Chinese Med |
| 1 | 0 | 2022 | Chang Gung Univ Sci & Technol |
| 1 | 0 | 2021 | Chinese Acad Tradit Chinese Med |
| 1 | 0 | 2022 | China Agr Univ |
| 1 | 0 | 2024 | Guangzhou Univ Chinese Med |
| 1 | 0 | 2021 | Guangdong Prov Key Lab Cell Microenviroment & Dis |
| 1 | 0 | 2020 | Huangshi Hosp Tradit Chinese Med |
| 1 | 0 | 2023 | Arjil Pharmaceut LLC |
| 1 | 0 | 2020 | Hubei Univ Tradit Chinese Med |
| 1 | 0.01 | 2024 | Dongguan Ninth Peoples Hosp |
| 1 | 0 | 2021 | Harbin Infect Dis Hosp |
| 1 | 0 | 2022 | Anhui Chest Hosp |
| 1 | 0 | 2021 | Bharathidasan Univ |
| 1 | 0 | 2022 | Beijing Univ Posts & Telecommun |
| 1 | 0 | 2023 | CBSin |
| 1 | 0 | 2020 | Beijing Polytech |
| 1 | 0 | 2021 | Childrens Hosp Orange Cty |
| 1 | 0 | 2020 | Berlin Inst Hlth |
| 1 | 0 | 2020 | Hubei Prov Hosp Tradit Chinese & Western Med |
| 1 | 0 | 2020 | Hong Kong Baptist Univ |
| 1 | 0 | 2022 | Beijing Forestry Univ |
| 1 | 0 | 2021 | BGC Trust Univ Bangladesh |
| 1 | 0 | 2022 | Brion Res Inst Taiwan |
| 1 | 0 | 2021 | Guangzhou Women & Childrens Med Ctr |
| 1 | 0 | 2022 | ABS Atlantico Business Sch |
| 1 | 0 | 2022 | Art Museum |
| 1 | 0 | 2024 | Nanyang First Peoples Hosp |
| 1 | 0 | 2020 | Guang Dong Second Tradit Chinese Med Hosp |
| 1 | 0 | 2020 | Guangzhou Univ Tradit Chinese Med |
| 1 | 0 | 2020 | Hubei Univ Chinese Med |
| 1 | 0 | 2024 | Jinyintan Hosp |
| 1 | 0 | 2023 | Chang Jung Christian Univ |
| 1 | 0 | 2021 | Dazhou Cent Hosp |
| 1 | 0 | 2020 | AFMC |
| 1 | 0 | 2023 | Ezhou Hosp Tradit Chinese Med |
| 1 | 0 | 2024 | Shanghai Inst Infect Dis & Biosecur |
| 1 | 0 | 2020 | Free Univ Berlin |
| 1 | 0 | 2022 | Baoan Authent TCM Therapy Hosp |
| 1 | 0 | 2020 | Heilongjiang Univ Chinese Med |
| 1 | 0 | 2022 | Al Mustaqbal Univ Coll |
| 1 | 0 | 2021 | Beijing Union Genius Pharmaceut Technol Dev Co Lt |
| 1 | 0 | 2020 | Chinese Univ Hong Kong |
| 1 | 0 | 2020 | Anhui Prov Cardiovasc Inst |
| 1 | 0 | 2022 | Beijing Hosp Tradit Chinese Med |
| 1 | 0 | 2023 | Chongqing Taiji Ind Grp Co Ltd |
| 1 | 0 | 2020 | Hong Zhi Tang Chinese Med Clin |
| 1 | 0 | 2021 | Bijie City First Peoples Hosp |
| 1 | 0.02 | 2023 | China Japan Friendship Hosp |
| 1 | 0 | 2024 | Fourth Hosp Inner Mongolia Autonomous Reg |
| 1 | 0 | 2021 | AJA Univ Med Sci |
| 1 | 0 | 2020 | Charite Univ Med Berlin |
| 1 | 0 | 2024 | Qingyuan Peoples Hosp |
| 1 | 0 | 2022 | CBSin Ctr BioSci Integrat Hlth |
| 1 | 0 | 2022 | Affiliated Hosp Hubei Univ Chinese Med |
| 1 | 0 | 2023 | Buddhist Tzu Chi Med Fdn |
| 1 | 0 | 2023 | Changzhou Inst Food Drug & Fiber Control |
| 1 | 0 | 2024 | Shandong Publ Hlth Clin Ctr |
| 1 | 0 | 2021 | Erciyes Univ |
| 1 | 0 | 2024 | Henan Chest Hosp |
| 1 | 0 | 2021 | Adamas Univ |
| 1 | 0 | 2024 | Jiangxi Univ Tradit Chinese Med |
| 1 | 0 | 2021 | Goa Univ |
| 1 | 0 | 2023 | Foshan Hosp Tradit Chinese Med |
| 1 | 0 | 2020 | Guangxi ASEAN Food Inspect & Testing Ctr |
| 1 | 0 | 2020 | Guizhou Univ Tradit Chinese Med |
| 1 | 0 | 2024 | First Hosp Nanchang |
| 1 | 0 | 2021 | Guangdong Med Univ |
| 1 | 0 | 2022 | Al MaMoon Univ Coll |
| 1 | 0 | 2023 | Chinese Acad Agr Sci |
| 1 | 0 | 2022 | Bengbu Med Coll |
| 1 | 0 | 2022 | BoYu Intelligent Hlth Innovat Lab |
| 1 | 0 | 2020 | Huangshi Hosp Youse |
| 1 | 0 | 2021 | Alagappa Univ |
| 1 | 0 | 2021 | Chungnam Natl Univ |
| 1 | 0 | 2022 | Biosource Tech |
| 1 | 0 | 2020 | Hebei Univ |
| 1 | 0 | 2024 | Heze Municipal Hosp |
| 1 | 0 | 2024 | Guangdong Second Prov Cent Hosp |
| 1 | 0 | 2021 | First Hosp Suihua City |
| 1 | 0 | 2024 | Guangzhou Lab |
| 1 | 0 | 2020 | Hebei Univ Chinese Med |
| 1 | 0 | 2023 | Agencia Nacl Vigilancia Sanit Anvisa |
| 1 | 0 | 2024 | Pingxiang Second Peoples Hosp |
| 1 | 0.01 | 2023 | Anhui Agr Univ |
| 1 | 0 | 2024 | ShangRao Peoples Hosp |
| 1 | 0 | 2020 | Amity Univ |
| 1 | 0 | 2021 | All India Inst Med Sci AIIMS |
| 1 | 0 | 2023 | Bharathiar Univ |

**Table S3.** The relationship parameters of authors that published literature on the TCM treatment of COVID-19.

| Count | Centrality | Year | Keywords |
| --- | --- | --- | --- |
| 11 | 0.1 | 2020 | Fang, Bangjiang |
| 11 | 0.02 | 2020 | Tong, Xiaolin |
| 11 | 0.07 | 2020 | Zhang, Ying |
| 9 | 0.14 | 2021 | Wang, Yu |
| 9 | 0.17 | 2020 | Liu, Qingquan |
| 9 | 0.1 | 2022 | Zhang, Han |
| 7 | 0.03 | 2021 | Zhang, Wen |
| 7 | 0.01 | 2021 | Li, Jing |
| 7 | 0.08 | 2020 | Lian, Fengmei |
| 7 | 0.21 | 2020 | Zhang, Lei |
| 6 | 0.08 | 2020 | Liu, Bin |
| 6 | 0.01 | 2021 | Zhou, Wei |
| 6 | 0.05 | 2020 | Yang, Zifeng |
| 5 | 0.01 | 2020 | Zhang, Wei |
| 5 | 0 | 2021 | Wang, Fei |
| 4 | 0.01 | 2022 | Gao, Yueqiu |
| 4 | 0 | 2020 | Wu, Hezhen |
| 4 | 0 | 2022 | Wang, Yan |
| 4 | 0 | 2023 | Ba, Yuanming |
| 4 | 0.02 | 2020 | Zhang, Qing |
| 4 | 0.01 | 2020 | Chen, Yaolong |
| 4 | 0.01 | 2020 | Che, Jinhua |
| 4 | 0.01 | 2022 | Chen, Bowu |
| 4 | 0 | 2021 | Li, Yan |
| 4 | 0.08 | 2020 | Zhang, Junhua |
| 4 | 0.03 | 2021 | Zhong, Nanshan |
| 4 | 0.01 | 2021 | Li, Lin |
| 4 | 0 | 2020 | Li, Qiang |
| 3 | 0 | 2021 | Ma, Qinhai |
| 3 | 0 | 2023 | Li, Wei |
| 3 | 0 | 2020 | Zheng, Yujiao |
| 3 | 0 | 2020 | Xu, Xi |
| 3 | 0 | 2020 | Wang, Zheyi |
| 3 | 0.01 | 2022 | Cao, Min |
| 3 | 0.01 | 2020 | Hong, Zongchao |
| 3 | 0 | 2020 | Li, Xiuyang |
| 3 | 0 | 2021 | Li, Ning |
| 3 | 0 | 2021 | Li, Bin |
| 3 | 0 | 2022 | Zhao, Ting |
| 3 | 0 | 2021 | Chen, Xuan |
| 3 | 0 | 2022 | Zhou, Shuang |
| 3 | 0 | 2022 | Wang, Yanping |
| 3 | 0 | 2021 | Qi, Wensheng |
| 3 | 0.02 | 2021 | Luo, Hui |
| 3 | 0 | 2021 | Li, Jun |
| 3 | 0 | 2023 | Zhang, Min |
| 3 | 0 | 2020 | Lu, Tao |
| 3 | 0.08 | 2021 | Wang, Jian |
| 2 | 0 | 2022 | Leung, Ka Wing |
| 2 | 0 | 2020 | Yang, Lu |
| 2 | 0.01 | 2021 | Chen, Yuqin |
| 2 | 0 | 2023 | Chang, Yanxu |
| 2 | 0 | 2020 | Liu, Han |
| 2 | 0 | 2023 | He, Shasha |
| 2 | 0 | 2022 | Gao, Xiumei |
| 2 | 0 | 2021 | Huang, Qinwan |
| 2 | 0 | 2022 | Ping, Yueh-Hsin |
| 2 | 0.01 | 2020 | Wang, Han |
| 2 | 0.01 | 2021 | He, Wenjun |
| 2 | 0 | 2021 | Liu, Yang |
| 2 | 0 | 2023 | Goncalves, Mario |
| 2 | 0 | 2021 | Lu, Yun-fei |
| 2 | 0 | 2022 | Lin, Shengying |
| 2 | 0.02 | 2021 | Lu, Cheng |
| 2 | 0 | 2022 | Lin, Chen-Shien |
| 2 | 0.01 | 2023 | Zhao, Jing |
| 2 | 0 | 2022 | Karimi, Mehrdad |
| 2 | 0.01 | 2022 | Li, Man |
| 2 | 0 | 2023 | Du, Kunze |
| 2 | 0 | 2021 | Li, Liang |
| 2 | 0.03 | 2023 | Yang, Jian |
| 2 | 0 | 2020 | Chen, Jing |
| 2 | 0 | 2023 | Fang, Jiansong |
| 2 | 0 | 2021 | Li, Tingna |
| 2 | 0 | 2023 | Zhang, Yuehong |
| 2 | 0 | 2020 | Li, Hong |
| 2 | 0.01 | 2020 | Li, Xiaodong |
| 2 | 0 | 2022 | Chu, Li-Wei |
| 2 | 0 | 2023 | Yuan, Weian |
| 2 | 0 | 2022 | Li, Xin |
| 2 | 0 | 2020 | Tian, Jinhui |
| 2 | 0 | 2022 | Hsueh, Chung-Chuan |
| 2 | 0 | 2022 | Lai, Keng Po |
| 2 | 0 | 2021 | Huang, Baoying |
| 2 | 0 | 2023 | Chen, Shujing |
| 2 | 0 | 2022 | Kazemi, Amir Hooman |
| 2 | 0 | 2023 | Fu, Lifeng |
| 2 | 0 | 2023 | Lv, Jia |
| 2 | 0 | 2022 | Liu, Yangyang |
| 2 | 0.01 | 2021 | Huang, Tingrong |
| 2 | 0.02 | 2021 | Liu, Zhigang |
| 2 | 0 | 2020 | Wu, Siying |
| 2 | 0 | 2020 | Li, Hui |
| 2 | 0 | 2021 | Chen, Rui |
| 2 | 0.02 | 2021 | Huang, Luqi |
| 2 | 0 | 2023 | Rodrigues, Jorge Magalhaes |
| 2 | 0.01 | 2021 | Fan, Tian |
| 2 | 0 | 2021 | Chen, Xiao-rong |
| 2 | 0.01 | 2020 | Min, Xiaojun |
| 2 | 0.01 | 2023 | Han, Lifeng |
| 2 | 0 | 2020 | Li, Shuang |
| 2 | 0 | 2022 | Lee, Hung Chun |
| 2 | 0 | 2020 | Yang, Fengwen |
| 2 | 0 | 2021 | Liu, Jia |
| 2 | 0 | 2022 | Lin, Jaung-Geng |
| 2 | 0 | 2023 | Wang, Yue |
| 2 | 0 | 2022 | Peng, Wei |
| 2 | 0 | 2020 | Chen, Fang |
| 2 | 0 | 2022 | Lu, Yang |
| 2 | 0 | 2023 | Wang, Ning |
| 2 | 0 | 2021 | Liu, Wei |
| 2 | 0 | 2023 | Li, Feng |
| 2 | 0 | 2022 | Alpert, Ruth |
| 2 | 0 | 2023 | Yang, Xiaohua |
| 2 | 0.02 | 2021 | Jiang, Yuyong |
| 2 | 0 | 2023 | Li, Jin |
| 2 | 0 | 2020 | Qu, Rendong |
| 2 | 0.01 | 2022 | Jiang, Haiming |
| 2 | 0 | 2020 | Yang, Ruocong |
| 2 | 0 | 2020 | Bai, Chen |
| 2 | 0 | 2022 | Huang, Yun |
| 2 | 0 | 2021 | Liu, Jing |
| 2 | 0 | 2020 | Pang, Bo |
| 2 | 0 | 2023 | Zhang, Yi |
| 2 | 0 | 2020 | Wei, Yu |
| 2 | 0 | 2020 | Ni, Li |
| 2 | 0.01 | 2021 | Liu, Chunli |
| 2 | 0 | 2023 | Shi, Wei |
| 2 | 0 | 2023 | Xu, Haiyu |
| 2 | 0.01 | 2022 | Lyu, Hua |
| 2 | 0 | 2021 | Liu, Ke |
| 2 | 0 | 2023 | Greten, Henry Johannes |
| 2 | 0.01 | 2020 | Li, Peng |
| 2 | 0 | 2020 | He, Tian |
| 2 | 0 | 2022 | Lai, Jennifer |
| 2 | 0 | 2022 | Fang, Min |
| 2 | 0 | 2022 | Li, Li |
| 2 | 0 | 2023 | Chen, Jie |
| 2 | 0 | 2022 | Chiou, Wen-Fei |
| 2 | 0 | 2023 | Shang, Ye |
| 2 | 0 | 2023 | Li, Xiaobing |
| 2 | 0 | 2023 | Wang, Xin |
| 2 | 0.01 | 2021 | Bai, Jianling |
| 2 | 0 | 2020 | Li, Min |
| 2 | 0 | 2023 | Zhuang, Qishuai |
| 2 | 0.02 | 2020 | Yang, Yanfang |
| 2 | 0.03 | 2020 | Wu, Haoran |
| 2 | 0.02 | 2021 | Bian, Yongjun |
| 2 | 0.01 | 2021 | Lu, Yun |
| 2 | 0 | 2022 | Gao, Zezheng |
| 2 | 0 | 2021 | Chen, Qi |
| 2 | 0 | 2020 | Li, Chang |
| 2 | 0 | 2023 | Gao, Hongwei |
| 2 | 0 | 2023 | Yu, Xueqing |
| 2 | 0 | 2023 | Wang, Xiaoying |
| 2 | 0 | 2023 | Tian, Yu |
| 2 | 0 | 2022 | An, Xuedong |
| 2 | 0.01 | 2021 | Lu, Wenju |
| 2 | 0 | 2023 | Tang, Qi |
| 2 | 0 | 2023 | Wang, Cheng |
| 2 | 0.02 | 2021 | Li, Hao |
| 2 | 0 | 2022 | Calloway, Teresa |
| 2 | 0.01 | 2022 | Huo, Xiaokui |
| 2 | 0.01 | 2021 | Cai, Jianxiong |
| 2 | 0.01 | 2020 | Liu, Baoyan |
| 2 | 0 | 2022 | Liaw, Chia-Ching |
| 2 | 0 | 2023 | Fang, Shiming |
| 2 | 0 | 2020 | Xu, Dan |
| 2 | 0 | 2021 | Feng, Jun |
| 2 | 0 | 2022 | Alipour, Reihane |
| 2 | 0 | 2021 | Lu, Li |
| 2 | 0 | 2021 | Chen, Yanyan |
| 2 | 0 | 2021 | He, Langchong |
| 2 | 0 | 2022 | Asadi, Asma |
| 2 | 0.03 | 2020 | Chen, Jianxin |
| 2 | 0 | 2020 | Wu, Xinxin |
| 2 | 0.03 | 2020 | Tian, Jiaxing |
| 2 | 0 | 2020 | Shao, Xiangming |
| 2 | 0 | 2020 | Huang, Yu-Feng |
| 2 | 0 | 2022 | Li, Qing |
| 2 | 0 | 2022 | Miller, Andrew L |
| 2 | 0 | 2022 | Choi, Sheyne S A |
| 2 | 0.05 | 2021 | Chen, Yang |
| 2 | 0 | 2022 | Hollifield, Michael |
| 2 | 0 | 2020 | Yang, Min |
| 2 | 0 | 2020 | Lin, Jiaran |
| 2 | 0 | 2020 | Chen, Ying |
| 2 | 0.01 | 2020 | Chen, Liang |
| 2 | 0.01 | 2022 | Ma, Xiaochi |
| 2 | 0 | 2021 | Lou, Guanhua |
| 2 | 0.05 | 2021 | Miao, Qing |
| 2 | 0 | 2021 | Ge, Guangbo |
| 2 | 0 | 2023 | Xu, Xiaolong |
| 2 | 0 | 2020 | Liu, Tiegang |
| 2 | 0 | 2021 | Liu, Lu-jiong |
| 2 | 0 | 2022 | Chen, Bor-Yann |
| 2 | 0 | 2021 | Fang, Shan |
| 1 | 0 | 2023 | Adel-mehraban, MohammadSadegh |
| 1 | 0 | 2023 | Agrawal, Apurva |
| 1 | 0 | 2024 | Lin, Feifei |
| 1 | 0 | 2024 | Pan, Lingyun |
| 1 | 0 | 2024 | Jiang, Mei |
| 1 | 0 | 2024 | Meng, Qingwei |
| 1 | 0 | 2024 | Ge, Fangqi |
| 1 | 0 | 2024 | Chen, Ruifeng |
| 1 | 0 | 2023 | Abduljaleel, Jinsha K |
| 1 | 0 | 2024 | Lin, Bingliang |
| 1 | 0 | 2023 | Abdolghaffari, Amir Hossein |
| 1 | 0 | 2023 | Adil, Muhammad |
| 1 | 0 | 2023 | Abbas, Zain |
| 1 | 0 | 2024 | Liang, Jingyi |
| 1 | 0 | 2024 | Li, Weiyang |
| 1 | 0 | 2024 | Huang, Chaolin |
| 1 | 0 | 2024 | Lu, Hongzhou |
| 1 | 0 | 2024 | Ou, Shuqiang |
| 1 | 0 | 2024 | Kang, Changyuan |
| 1 | 0 | 2024 | Li, Yueping |
| 1 | 0 | 2024 | Chen, Dexiong |
| 1 | 0 | 2024 | Li, Xiaoqian |
| 1 | 0 | 2024 | Lin, Zhengshi |
| 1 | 0 | 2024 | Guo, Xiaoyun |
| 1 | 0 | 2024 | Gao, Dan |
| 1 | 0 | 2024 | Li, Haijun |
| 1 | 0 | 2024 | Lin, Ling |
| 1 | 0 | 2024 | Luo, Jincan |
| 1 | 0 | 2024 | Fang, Zhonghao |
| 1 | 0 | 2024 | Pan, Hongqiu |
| 1 | 0 | 2024 | Liang, Shiwei |
| 1 | 0 | 2024 | Deng, Ying |

**Table S4.** The relationship parameters of the keywords that published literature on the TCM treatment of COVID-19.

| Count | Centrality | Year | Keywords |
| --- | --- | --- | --- |
| 29 | 0.27 | 2020 | traditional chinese medicine |
| 23 | 0.13 | 2020 | covid 19 |
| 19 | 0.15 | 2020 | molecular docking |
| 12 | 0.18 | 2020 | acute lung injury |
| 11 | 0.04 | 2020 | network pharmacology |
| 10 | 0.19 | 2020 | protein |
| 9 | 0.13 | 2021 | activation |
| 9 | 0.08 | 2021 | inflammation |
| 9 | 0.09 | 2020 | molecular dynamics |
| 9 | 0.1 | 2020 | coronavirus |
| 7 | 0.1 | 2020 | infection |
| 7 | 0.02 | 2022 | identification |
| 7 | 0.17 | 2020 | sar |
| 6 | 0.04 | 2021 | inhibition |
| 6 | 0.17 | 2020 | coronavirus disease 2019 |
| 6 | 0.02 | 2021 | decoction |
| 6 | 0.16 | 2021 | in vitro |
| 6 | 0.18 | 2020 | cytokine storm |
| 5 | 0.03 | 2021 | natural product |
| 5 | 0.08 | 2022 | metabolism |
| 5 | 0 | 2020 | pneumonia |
| 5 | 0.12 | 2021 | apoptosis |
| 5 | 0.09 | 2021 | docking |
| 5 | 0.04 | 2022 | sars cov 2 |
| 4 | 0.01 | 2020 | clinical characteristics |
| 4 | 0.04 | 2022 | inhibitor |
| 4 | 0.04 | 2022 | randomized controlled trial |
| 4 | 0.03 | 2022 | pharmacology |
| 4 | 0.04 | 2021 | molecular dynamics simulation |
| 4 | 0.09 | 2022 | component |
| 4 | 0.09 | 2022 | model |
| 3 | 0.11 | 2021 | antiviral activity |
| 3 | 0.04 | 2022 | ace2 |
| 3 | 0 | 2022 | cell |
| 3 | 0.08 | 2020 | acute respiratory syndrome |
| 3 | 0.01 | 2022 | response |
| 3 | 0.01 | 2022 | mechanism |
| 2 | 0 | 2022 | virus |
| 2 | 0 | 2023 | essential oil |
| 2 | 0.01 | 2022 | glycoside |
| 2 | 0.06 | 2023 | macrophage polarization |
| 2 | 0.01 | 2022 | spike |
| 2 | 0.1 | 2021 | main protease |
| 2 | 0 | 2020 | mechanism of action |
| 2 | 0 | 2022 | herbal medicine |
| 2 | 0.01 | 2023 | extract |
| 2 | 0 | 2021 | pharmacokinetics |
| 2 | 0 | 2023 | online teaching |
| 2 | 0.05 | 2023 | injury |
| 2 | 0 | 2020 | china |
| 2 | 0 | 2022 | intervention |
| 2 | 0.02 | 2022 | scutellaria baicalensis |
| 2 | 0 | 2022 | primary care |
| 2 | 0.01 | 2022 | mass spectrometry |
| 2 | 0.01 | 2023 | pathway |
| 2 | 0.01 | 2023 | marker |
| 2 | 0.01 | 2022 | fruit |
| 2 | 0.02 | 2023 | higher education |
| 2 | 0.01 | 2020 | nf kappa b |
| 2 | 0 | 2021 | sars cov |
| 2 | 0 | 2023 | treatment outcome |
| 2 | 0.01 | 2023 | ethanol extract |
| 2 | 0 | 2023 | clinical feature |
| 2 | 0.06 | 2021 | flavonoid |
| 2 | 0.02 | 2023 | linoleic acid |
| 2 | 0.03 | 2022 | simulation |
| 2 | 0.02 | 2022 | natural compound |
| 2 | 0.06 | 2022 | mental health |
| 2 | 0 | 2022 | oxidative stress |
| 2 | 0.06 | 2021 | strategy |
| 2 | 0 | 2023 | efficacy |
| 2 | 0.04 | 2022 | rat plasma |
| 2 | 0.03 | 2021 | glide |
| 2 | 0.12 | 2021 | medicinal plant |
| 2 | 0 | 2022 | medicine |
| 2 | 0.04 | 2023 | antiviral drug |
| 2 | 0 | 2023 | pulmonary fibrosis |
| 2 | 0 | 2020 | prediction |
| 2 | 0 | 2023 | radix |
| 2 | 0 | 2023 | chinese medicine |
| 2 | 0.01 | 2020 | mice |
| 2 | 0 | 2023 | induction |
| 2 | 0.08 | 2021 | papain-like protease |
| 2 | 0.01 | 2021 | gromac |
| 2 | 0.04 | 2022 | management |
| 1 | 0 | 2022 | adjustment |
| 1 | 0 | 2020 | coronavirus disease 2019 (covid-19) |
| 1 | 0.02 | 2020 | coronavirus pneumonia |
| 1 | 0 | 2023 | air distribution |
| 1 | 0.03 | 2020 | clinical classification |
| 1 | 0 | 2023 | allicin |
| 1 | 0 | 2024 | drug-drug interaction |
| 1 | 0 | 2020 | aflatoxin b1 |
| 1 | 0 | 2021 | anxiety |
| 1 | 0 | 2020 | autodock vina |
| 1 | 0 | 2023 | age |
| 1 | 0 | 2024 | care |
| 1 | 0 | 2021 | auricular acupuncture |
| 1 | 0 | 2024 | anti sars-cov-2 drug |
| 1 | 0 | 2022 | acetylcholine receptor (achr) |
| 1 | 0 | 2022 | acute cerebral infarction |
| 1 | 0.01 | 2022 | achievement |
| 1 | 0 | 2020 | critical illness |
| 1 | 0 | 2021 | antidepressant |
| 1 | 0 | 2022 | agaricus blazei murill |
| 1 | 0 | 2023 | agent |
| 1 | 0 | 2020 | 3c-like protease |
| 1 | 0 | 2021 | 3cl protease inhibitor |
| 1 | 0 | 2023 | adolescent |
| 1 | 0 | 2020 | collapse |
| 1 | 0 | 2024 | xuanbai chengqi decoction |
| 1 | 0 | 2020 | computational drug discovery |
| 1 | 0 | 2023 | adulteration |
| 1 | 0 | 2020 | chinese herbal medicine |
| 1 | 0.02 | 2021 | aqueous extract |
| 1 | 0 | 2023 | adhd |
| 1 | 0.02 | 2022 | aconitum carmichaelii |
| 1 | 0 | 2020 | conut scor |
| 1 | 0.01 | 2022 | acute bronchiti |
| 1 | 0 | 2020 | coix seed |
| 1 | 0 | 2021 | auricular massage |
| 1 | 0 | 2022 | acetylcholinesterase inhibitor |
| 1 | 0.01 | 2022 | 3-4-dihydroxybenzalacetone |
| 1 | 0 | 2023 | acteoside |
| 1 | 0.02 | 2020 | coronavirus disease |
| 1 | 0 | 2020 | bat coronavirus |
| 1 | 0 | 2020 | angiotensin converting enzyme |
| 1 | 0.03 | 2020 | chikungunya |
| 1 | 0 | 2021 | andrographis paniculata extract |
| 1 | 0 | 2021 | adverse reaction |
| 1 | 0 | 2024 | biology |
| 1 | 0.01 | 2020 | angiotensin-converting enzyme 2 |
| 1 | 0 | 2023 | allium sativum |
| 1 | 0 | 2021 | active constituent |
| 1 | 0 | 2022 | acute ischemic stroke |
| 1 | 0 | 2021 | antiproliferative activity |
| 1 | 0.02 | 2020 | blocking |
| 1 | 0 | 2021 | arbidol |
| 1 | 0.01 | 2022 | a(2a) |
| 1 | 0 | 2023 | 2' deoxy 2' beta fluoro 4' azidocytidine |
| 1 | 0 | 2021 | antibody |
| 1 | 0 | 2021 | assisted laser desorption/ionization |
| 1 | 0.01 | 2022 | activator |
| 1 | 0 | 2024 | amygdalin |
| 1 | 0 | 2021 | 3a4 |
| 1 | 0 | 2023 | american college |
| 1 | 0 | 2023 | acid |
| 1 | 0 | 2021 | ambient condition |
| 1 | 0.01 | 2022 | adenosine receptor |
| 1 | 0 | 2022 | adherence |
| 1 | 0 | 2023 | aerosol transportation |
| 1 | 0.04 | 2023 | anakinra |
| 1 | 0 | 2020 | cancer |
| 1 | 0.01 | 2020 | compound kushen injection |
| 1 | 0 | 2020 | core decompression |
| 1 | 0 | 2023 | adaptability |
| 1 | 0 | 2021 | accurate docking |
| 1 | 0 | 2021 | antioxidant |
| 1 | 0.02 | 2023 | active component |
| 1 | 0 | 2021 | alkaloid |
| 1 | 0 | 2021 | antibiotics |
| 1 | 0 | 2020 | cholesterol |
| 1 | 0.01 | 2022 | abiotic stresse |
| 1 | 0 | 2020 | blood lymphocyte count |
| 1 | 0 | 2021 | atf2 |
| 1 | 0 | 2020 | alendronate |
| 1 | 0 | 2024 | nlrp3 inflammasome |
| 1 | 0 | 2020 | case report |
| 1 | 0 | 2020 | clinical treatment |

**Table S5.** Annotation of signaling pathways related to the treatment of COVID-19 by Gancao─Gegen.

| Term | Description | LogP | Log(q-value) | Symbols |
| --- | --- | --- | --- | --- |
| hsa05205 | Proteoglycans in cancer | −14.35365858 | −10.00103234 | CTSL\|EGFR\|ESR1\|MTOR\|ITGB1\|PIK3CD\|PRKACA\|TNF\|VEGFA\|HPSE |
| hsa05165 | Human papillomavirus infection | −10.62248081 | −6.74697583 | EGFR\|MTOR\|HDAC2\|ITGB1\|PIK3CD\|PRKACA\|TNF\|VEGFA\|TBK1 |
| hsa05200 | Pathways in cancer | −8.811958026 | −5.362421776 | AGTR1\|EGFR\|ESR1\|MTOR\|HDAC2\|ITGB1\|PIK3CD\|PRKACA\|VEGFA |
| hsa05163 | Human cytomegalovirus infection | −8.691003747 | −5.338377511 | EGFR\|MTOR\|PIK3CD\|PRKACA\|TNF\|VEGFA\|TBK1 |
| hsa04140 | Autophagy - animal | −8.298635811 | −4.987402259 | CTSB\|CTSL\|MTOR\|PIK3CD\|PRKACA\|TBK1 |
| hsa01522 | Endocrine resistance | −7.363605544 | −4.289732908 | EGFR\|ESR1\|MTOR\|PIK3CD\|PRKACA |
| hsa05207 | Chemical carcinogenesis - receptor activation | −7.241178376 | −4.223529673 | EGFR\|ESR1\|MTOR\|PIK3CD\|PRKACA\|VEGFA |
| hsa04919 | Thyroid hormone signaling pathway | −6.904042371 | −4.014863412 | ESR1\|MTOR\|HDAC2\|PIK3CD\|PRKACA |
| hsa04611 | Platelet activation | −6.850870379 | −4.014863412 | BTK\|ITGB1\|PIK3CD\|PIK3CG\|PRKACA |
| hsa05131 | Shigellosis | −6.848975709 | −4.014863412 | EGFR\|MTOR\|ITGB1\|PIK3CD\|TNF\|TBK1 |
| hsa04371 | Apelin signaling pathway | −6.603535849 | −3.852969604 | AGTR1\|MTOR\|PIK3CG\|PLAT\|PRKACA |
| hsa05418 | Fluid shear stress and atherosclerosis | −6.603535849 | −3.852969604 | CTSL\|PIK3CD\|PLAT\|TNF\|VEGFA |
| hsa04072 | Phospholipase D signaling pathway | −6.468060457 | −3.778192052 | AGTR1\|EGFR\|MTOR\|PIK3CD\|PIK3CG |
| hsa04213 | Longevity regulating pathway - multiple species | −6.373369659 | −3.736746766 | MTOR\|HDAC2\|PIK3CD\|PRKACA |
| hsa05212 | Pancreatic cancer | −6.015852753 | −3.448534502 | EGFR\|MTOR\|PIK3CD\|VEGFA |
| hsa01521 | EGFR tyrosine kinase inhibitor resistance | −5.948153539 | −3.415071237 | EGFR\|MTOR\|PIK3CD\|VEGFA |
| hsa04151 | PI3K-Akt signaling pathway | −5.934857656 | −3.408306222 | EGFR\|MTOR\|ITGB1\|PIK3CD\|PIK3CG\|VEGFA |
| hsa05167 | Kaposi sarcoma-associated herpesvirus infection | −5.887078033 | −3.373300887 | MTOR\|PIK3CD\|PIK3CG\|VEGFA\|TBK1 |
| hsa05169 | Epstein-Barr virus infection | −5.800830512 | −3.325906671 | BTK\|HDAC2\|PIK3CD\|TNF\|TBK1 |
| hsa05235 | PD-L1 expression and PD-1 checkpoint pathway in cancer | −5.74028034 | −3.283442925 | EGFR\|MTOR\|PIK3CD\|TLR9 |

**Table S6.** Common feature pharmacophore generation.

| Model | Features | Rank | Direct Hit | Partial Hit | Max Fit |
| --- | --- | --- | --- | --- | --- |
| 01 | HAA | 39.917 | 111111 | 0 | 3 |
| 02 | HAA | 39.334 | 111111 | 0 | 3 |
| 03 | HAA | 38.872 | 111111 | 0 | 3 |
| 04 | HAA | 37.738 | 111111 | 0 | 3 |
| 05 | HAA | 37.291 | 111111 | 0 | 3 |
| 06 | HAA | 35.891 | 111111 | 0 | 3 |
| 07 | HAA | 35.743 | 111111 | 0 | 3 |
| 08 | HAA | 34.531 | 111111 | 0 | 3 |
| 09 | HAA | 34.263 | 111111 | 0 | 3 |
| 10 | HAA | 34.03 | 111111 | 0 | 3 |

**Table S7.** The key sites of GLA binding to different targets were investigated by alanine scanning mutation technique.

| **Targets** | **Mutation** | **Mutation energy** | **Effect** | **VDW** | **Electrostatic** | **Entropy** |
| --- | --- | --- | --- | --- | --- | --- |
| 3CL pro | GLY143>ALA | −0.07 | NEUTRAL | −0.19 | 0.03 | 0.01 |
|  | THR190>ALA | 0.04 | NEUTRAL | 0.11 | −0.03 | 0 |
|  | THR26>ALA | 0.06 | NEUTRAL | 0.13 | −0.02 | 0 |
|  | CYS145>ALA | 0.06 | NEUTRAL | 0.08 | 0.04 | 0 |
|  | LEU27>ALA | 0.2 | NEUTRAL | 0.46 | −0.07 | 0.01 |
|  | THR25>ALA | 0.31 | NEUTRAL | 0.85 | −0.11 | −0.08 |
|  | PRO168>ALA | 0.43 | NEUTRAL | 0.84 | 0.03 | −0.01 |
|  | MET165>ALA | 0.5 | NEUTRAL | 1.11 | −0.06 | −0.03 |
|  | ASN142>ALA | 0.68 | DESTABILIZING | 1.76 | −0.18 | −0.14 |
|  | GLN189>ALA | 0.7 | DESTABILIZING | 3.45 | −0.27 | −1.11 |
| ACE2 | LYS26>ALA | −0.33 | NEUTRAL | 0.28 | 0.02 | −0.6 |
|  | THR92>ALA | −0.1 | NEUTRAL | 0.25 | −0.07 | −0.24 |
|  | ASP30>ALA | 0.01 | NEUTRAL | 1.1 | −0.61 | −0.3 |
|  | PRO389>ALA | 0.3 | NEUTRAL | 0.57 | 0.02 | 0.01 |
|  | LEU29>ALA | 0.33 | NEUTRAL | 0.74 | −0.1 | 0.01 |
|  | VAL93>ALA | 0.41 | NEUTRAL | 0.58 | −0.04 | 0.17 |
|  | ASN33>ALA | 0.42 | NEUTRAL | 0.96 | −0.1 | −0.01 |
|  | GLN96>ALA | 0.79 | DESTABILIZING | 1.78 | −0.19 | −0.01 |
| Plp | GLN270>ALA | −0.13 | NEUTRAL | 1.26 | −0.03 | −0.93 |
|  | LEU163>ALA | 0.52 | DESTABILIZING | 1.85 | −0.04 | −0.48 |
|  | TYR269>ALA | 1.57 | DESTABILIZING | 3.8 | −0.22 | −0.27 |
| TNF | ALA38>ALA | −0.01 | NEUTRAL | −0.01 | 0 | 0 |
|  | ALA14>ALA | 0 | NEUTRAL | 0 | 0 | 0 |
|  | SER147>ALA | 0 | NEUTRAL | 0.05 | −0.02 | −0.02 |
|  | VAL13>ALA | 0.17 | NEUTRAL | 0.64 | −0.02 | −0.17 |
|  | GLY148>ALA | 0.85 | DESTABILIZING | 1.68 | 0.02 | 0 |
|  | HIS15>ALA | 0.91 | DESTABILIZING | 2.02 | −0.15 | −0.03 |
|  | LEU36>ALA | 1.03 | DESTABILIZING | 2.88 | −0.06 | −0.47 |

**Table S8.** The key sites of PUE binding to different targets were investigated by alanine scanning mutation technique.

| **Targets** | **Mutation** | **Mutation energy** | **Effect** | **VDW** | **Electrostatic** | **Entropy** |
| --- | --- | --- | --- | --- | --- | --- |
| 3CL pro | GLU166>ALA | −0.15 | NEUTRAL | 0.27 | −0.22 | −0.22 |
|  | GLY143>ALA | −0.03 | NEUTRAL | −0.09 | 0.03 | 0 |
|  | ASP187>ALA | −0.03 | NEUTRAL | 0.08 | −0.15 | 0 |
|  | CYS145>ALA | 0.05 | NEUTRAL | 0.17 | −0.08 | 0 |
|  | ARG188>ALA | 0.06 | NEUTRAL | 0.1 | 0.01 | 0 |
|  | LEU27>ALA | 0.15 | NEUTRAL | 0.39 | −0.1 | 0.01 |
|  | HIS164>ALA | 0.44 | NEUTRAL | 1.09 | −0.25 | 0.03 |
|  | PRO168>ALA | 0.49 | NEUTRAL | 0.92 | 0.05 | 0 |
|  | GLN189>ALA | 0.57 | DESTABILIZING | 2.37 | −0.07 | −0.72 |
|  | MET165>ALA | 0.87 | DESTABILIZING | 1.72 | 0.02 | 0 |
|  | MET49>ALA | 1.04 | DESTABILIZING | 1.53 | −0.09 | 0.4 |
|  | HIS41>ALA | 1.05 | DESTABILIZING | 2.28 | −0.21 | 0.02 |
| ACE2 | ALA387>ALA | −0.36 | NEUTRAL | −0.6 | −0.05 | −0.04 |
|  | ASP30>ALA | −0.13 | NEUTRAL | 0.81 | −0.51 | −0.35 |
|  | GLU37>ALA | −0.11 | NEUTRAL | 0.63 | −0.41 | −0.28 |
|  | GLN388>ALA | 0.03 | NEUTRAL | 0.08 | −0.02 | 0 |
|  | ASN33>ALA | 0.18 | NEUTRAL | 0.61 | −0.24 | −0.01 |
|  | PHE390>ALA | 0.18 | NEUTRAL | 1.23 | −1.03 | 0.1 |
|  | LYS353>ALA | 0.29 | NEUTRAL | 1.37 | −0.24 | −0.34 |
|  | ARG393>ALA | 0.59 | DESTABILIZING | 0.92 | 0.14 | 0.07 |
|  | HIS34>ALA | 0.64 | DESTABILIZING | 2.16 | −0.19 | −0.43 |
|  | PRO389>ALA | 0.69 | DESTABILIZING | 1.24 | 0.16 | −0.01 |
| Plp | GLN270>ALA | 0.21 | NEUTRAL | 1.35 | −0.34 | −0.37 |
|  | TYR274>ALA | 0.74 | DESTABILIZING | 1.87 | −0.33 | −0.04 |
|  | LEU163>ALA | 0.85 | DESTABILIZING | 2.54 | −0.14 | −0.44 |
|  | TYR269>ALA | 2.7 | DESTABILIZING | 6.42 | −0.37 | −0.41 |
| TNF | GLY121>ALA | −0.28 | NEUTRAL | −0.66 | 0.1 | 0 |
|  | LEU120>ALA | −0.03 | NEUTRAL | 0.05 | −0.12 | 0 |
|  | PRO117>ALA | 0.44 | NEUTRAL | 0.78 | −0.02 | 0.07 |
|  | LEU57>ALA | 0.48 | NEUTRAL | 1.2 | −0.18 | −0.04 |
|  | TYR119>ALA | 0.51 | DESTABILIZING | 1.33 | −0.5 | 0.12 |
|  | VAL123>ALA | 0.69 | DESTABILIZING | 1.52 | −0.22 | 0.05 |
|  | TYR59>ALA | 0.8 | DESTABILIZING | 2.07 | −0.46 | 0 |
|  | LEU55>ALA | 0.88 | DESTABILIZING | 1.91 | −0.37 | 0.14 |
|  | GLN61>ALA | 0.95 | DESTABILIZING | 1.81 | −0.05 | 0.09 |
|  | GLY121>ALA | 1.28 | DESTABILIZING | 2.47 | 0.12 | −0.02 |
|  | TYR119>ALA | 1.62 | DESTABILIZING | 3.96 | −0.68 | −0.03 |

**Table S9.** The binding ability of PUE─PUE.

| mode | affinity (kcal/mol) | dist from rmsd l.b. | best mode rmsd u.b. |
| --- | --- | --- | --- |
| 1 | −4.4 | 0.000 | 0.000 |
| 2 | −4.1 | 7.626 | 10.142 |
| 3 | −4.0 | 2.940 | 8.587 |
| 4 | −4.0 | 2.681 | 2.981 |
| 5 | −3.9 | 7.447 | 11.004 |
| 6 | −3.8 | 1.251 | 2.601 |
| 7 | −3.8 | 2.810 | 8.879 |
| 8 | −3.7 | 2.515 | 7.890 |
| 9 | −3.7 | 2.959 | 4.565 |

**Table S10.** The binding ability of GLA─PUE.

| mode | affinity (kcal/mol) | dist from rmsd l.b. | best mode rmsd u.b. |
| --- | --- | --- | --- |
| 1 | −4.0 | 0.000 | 0.000 |
| 2 | −4.0 | 1.441 | 2.588 |
| 3 | −3.9 | 7.052 | 8.330 |
| 4 | −3.8 | 6.979 | 8.154 |
| 5 | −3.7 | 6.598 | 8.200 |
| 6 | −3.7 | 2.022 | 8.039 |
| 7 | −3.6 | 2.120 | 8.238 |
| 8 | −3.5 | 6.809 | 10.851 |
| 9 | −3.5 | 3.302 | 8.330 |

**Table S11.** The binding ability of ACE2─PUE.

| mode | affinity (kcal/mol) | dist from rmsd l.b. | best mode rmsd u.b. |
| --- | --- | --- | --- |
| 1 | −6.7 | 0.000 | 0.000 |
| 2 | −6.3 | 1.786 | 8.826 |
| 3 | −6.3 | 2.424 | 3.213 |
| 4 | −6.2 | 17.684 | 21.609 |
| 5 | −6.0 | 2.699 | 8.380 |
| 6 | −6.0 | 4.020 | 10.178 |
| 7 | −6.0 | 7.796 | 10.332 |
| 8 | −6.0 | 1.634 | 2.114 |
| 9 | −6.0 | 5.675 | 9.176 |

**Table S12.** Pharmacokinetic and drug-likeness assessment of PUE.

| Category | Parameter | Value |
| --- | --- | --- |
| Physicochemical Properties | Formula | C21H20O9 |
|  | Molecular weight | 416.38 g/mol |
|  | Num. heavy atoms | 30 |
|  | Num. arom. heavy atoms | 16 |
|  | Fraction Csp3 | 0.29 |
|  | Num. rotatable bonds | 3 |
|  | Num. H-bond acceptors | 9 |
|  | Num. H-bond donors | 6 |
|  | Molar Refractivity | 104.59 |
|  | TPSA | 160.82 Å² |
| Lipophilicity | Log *P*_o/w_ (iLOGP) | 1.77 |
|  | Log *P*_o/w_ (XLOGP3) | 0.01 |
|  | Log *P*_o/w_ (WLOGP) | 0.06 |
|  | Log *P*_o/w_ (MLOGP) | −1.52 |
|  | Log *P*_o/w_ (SILICOS-IT) | 0.81 |
|  | Consensus Log *P*_o/w_ | 0.23 |
| Water Solubility | Log *S* (ESOL) | −2.62 |
|  | Solubility | 9.89e-01 mg/ml; 2.37e-03 mol/l |
|  | Class | Soluble |
|  | Log *S* (Ali) | −2.94 |
|  | Solubility | 4.79e-01 mg/ml; 1.15e-03 mol/l |
|  | Class | Soluble |
|  | Log *S* (SILICOS-IT) | −2.97 |
|  | Solubility | 4.49e-01 mg/ml; 1.08e-03 mol/l |
|  | Class | Soluble |
| Pharmacokinetics | GI absorption | Low |
|  | BBB permeant | No |
|  | P-gp substrate | No |
|  | CYP1A2 inhibitor | No |
|  | CYP2C19 inhibitor | No |
|  | CYP2C9 inhibitor | No |
|  | CYP2D6 inhibitor | No |
|  | CYP3A4 inhibitor | No |
|  | Log *K*_p_ (skin permeation) | −8.83 cm/s |
| Druglikeness | Lipinski | Yes; 1 violation: NHorOH>5 |
|  | Ghose | Yes |
|  | Veber | No; 1 violation: TPSA>140 |
|  | Egan | No; 1 violation: TPSA>131.6 |
|  | Muegge | No; 2 violations: TPSA>150, H-don>5 |
|  | Bioavailability Score | 0.55 |
| Medicinal Chemistry | PAINS | 0 alert |
|  | Brenk | 0 alert |
|  | Leadlikeness | No; 1 violation: MW>350 |
|  | Synthetic accessibility | 4.98 |

**Table S13.** Pharmacokinetic and drug-likeness assessment of GLA.

| Category | Parameter | Value |
| --- | --- | --- |
| Physicochemical Properties | Formula | C20H20O4 |
|  | Molecular weight | 324.37 g/mol |
|  | Num. heavy atoms | 24 |
|  | Num. arom. heavy atoms | 12 |
|  | Fraction Csp3 | 0.30 |
|  | Num. rotatable bonds | 1 |
|  | Num. H-bond acceptors | 4 |
|  | Num. H-bond donors | 2 |
|  | Molar Refractivity | 93.25 |
|  | TPSA | 58.92 Å² |
| Lipophilicity | Log *P*_o/w_ (iLOGP) | 2.97 |
|  | Log *P*_o/w_ (XLOGP3) | 3.89 |
|  | Log *P*_o/w_ (WLOGP) | 3.89 |
|  | Log *P*_o/w_ (MLOGP) | 2.73 |
|  | Log *P*_o/w_ (SILICOS-IT) | 3.76 |
|  | Consensus Log *P*_o/w_ | 3.45 |
| Water Solubility | Log *S* (ESOL) | −4.61 |
|  | Solubility | 8.04e-03 mg/ml; 2.48e-05 mol/l |
|  | Class | Moderately soluble |
|  | Log *S* (Ali) | −4.83 |
|  | Solubility | 4.85e-03 mg/ml; 1.50e-05 mol/l |
|  | Class | Moderately soluble |
|  | Log *S* (SILICOS-IT) | −4.95 |
|  | Solubility | 3.67e-03 mg/ml; 1.13e-05 mol/l |
|  | Class | Moderately soluble |
| Pharmacokinetics | GI absorption | High |
|  | BBB permeant | Yes |
|  | P-gp substrate | Yes |
|  | CYP1A2 inhibitor | Yes |
|  | CYP2C19 inhibitor | Yes |
|  | CYP2C9 inhibitor | Yes |
|  | CYP2D6 inhibitor | Yes |
|  | CYP3A4 inhibitor | Yes |
|  | Log *K*_p_ (skin permeation) | −5.52 cm/s |
| Druglikeness | Lipinski | Yes; 0 violation |
|  | Ghose | Yes |
|  | Veber | Yes |
|  | Egan | Yes |
|  | Muegge | Yes |
|  | Bioavailability Score | 0.55 |
| Medicinal Chemistry | PAINS | 0 alert |
|  | Brenk | 0 alert |
|  | Leadlikeness | No; 1 violation: XLOGP3>3.5 |
|  | Synthetic accessibility | 4.04 |
